# Enantioselective Self-Replicators

**DOI:** 10.1021/jacs.3c05472

**Published:** 2023-07-24

**Authors:** Shuo Yang, Yannick Geiger, Marc Geerts, Marcel J. Eleveld, Armin Kiani, Sijbren Otto

**Affiliations:** † State Key Laboratory of Metal Matrix Composites, School of Materials Science and Engineering, Shanghai Jiao Tong University, Shanghai 200240, P. R. China; ‡ Centre for Systems Chemistry, Stratingh Institute, University of Groningen, Groningen 9747 AG, The Netherlands; § Zhangjiang Institute for Advanced Study (ZIAS), Shanghai Jiao Tong University, Shanghai 201203, P. R. China

## Abstract

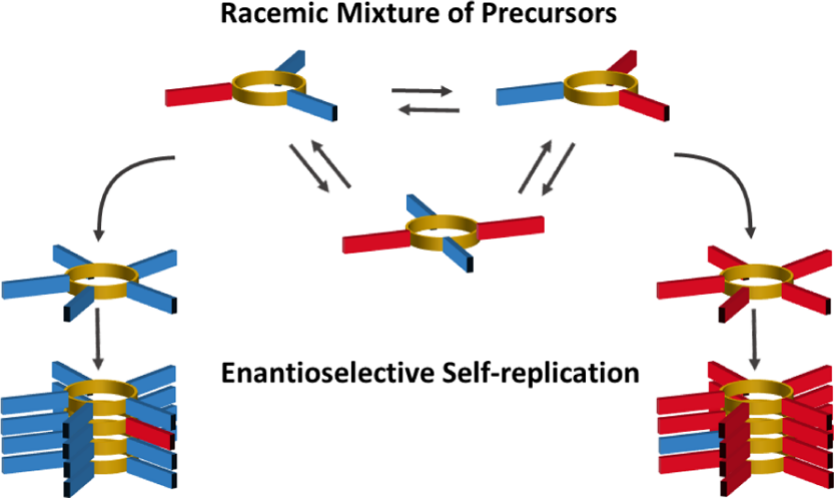

Self-replicating molecules provide a simple approach for investigating fundamental processes in scenarios of the emergence of life. Although homochirality is an important aspect of life and of how it emerged, the effects of chirality on self-replicators have received only little attention so far. Here, we report several self-assembled self-replicators with enantioselectivity that emerge spontaneously and grow only from enantiopure material. These require a relatively small number of chiral units in the replicators (down to eight) and in the precursors (down to a single chiral unit), compared to the only other enantioselective replicator reported previously. One replicator was found to incorporate material of its own handedness with high fidelity when provided with a racemic mixture of precursors, thus sorting (L)- and (D)-precursors into (L)- and (D)-replicators. Systematic studies reveal that the presence or absence of enantioselectivity depends on structural features (ring size of the replicator) that appear to impose constraints on its supramolecular organization. This work reveals new aspects of the little researched interplay between chirality and self-replication and represents another step toward the de novo synthesis of life.

## Introduction

How life can emerge from inanimate matter is one of the grand mysteries of science and has fascinated generations of scientists.^1−5^ An important aspect of life is its homochirality: in extant life, sugars and amino acids exist only as one of the two possible enantiomers (right- and left-handed, respectively), with only few exceptions.^6^ Much more than a simple curiosity of nature, it is essential for the exceptional efficiency in information transfer,^6−8^ catalysis,^9^ and electron transfer^10^ of today’s biomolecules and confers directionality to molecular and cellular motion.^11−13^ Hence, homochirality is likely to play an important role in the emergence of function in the transition from chemistry to biology.^8,14,15^ How biological homochirality arose from a (close to) racemic world is thus a question that intrigues. In the past decades, the scientific community has devoted much effort to study chiral amplification^16−19^ and symmetry breaking processes.^20,21^ Most of these efforts have been directed at the polymerization of peptides and oligonucleotides,^21−25^ crystallization,^26,27^ and asymmetric autocatalysis of small molecules,^28−32^ of which the Soai reaction is a famous example.^33−39^ An overview of these systems is provided in (20) and (21). In many instances, autocatalysis plays an important role in the emergence of homochirality. Surprisingly, the influence of chirality in self-replicating systems (a special class of autocatalytic systems, where autocatalysis is accompanied by transfer of information, beyond chirality) has received very little attention despite the central role of self-replication^40,41^ in the emergence of life. Enantioselectivity and persistence have been observed only in a system of self-replicating peptides developed by Ghadiri and co-workers.^42,43^ In this system, an α-helical peptide copies itself by binding to and thereby promoting the ligation of two subunits. Autocatalysis is observed only if the peptide and the subunits all have the same handedness; single stereochemical mutations are tolerated only in the template strand. However, as a model system for early evolution, it has some limitations: it consists of a rather long homochiral peptide (31 residues of identical chirality) with a specific sequence, which is unlikely to arise spontaneously in a racemic environment; and its replication requires homochiral oligomers (containing 14, respectively, 17 residues of identical chirality) as precursors, which are also unlikely to arise spontaneously, and the parabolic (as opposed to exponential) growth kinetics diminish its potential to undergo Darwinian evolution.^44^

**Figure 1:**
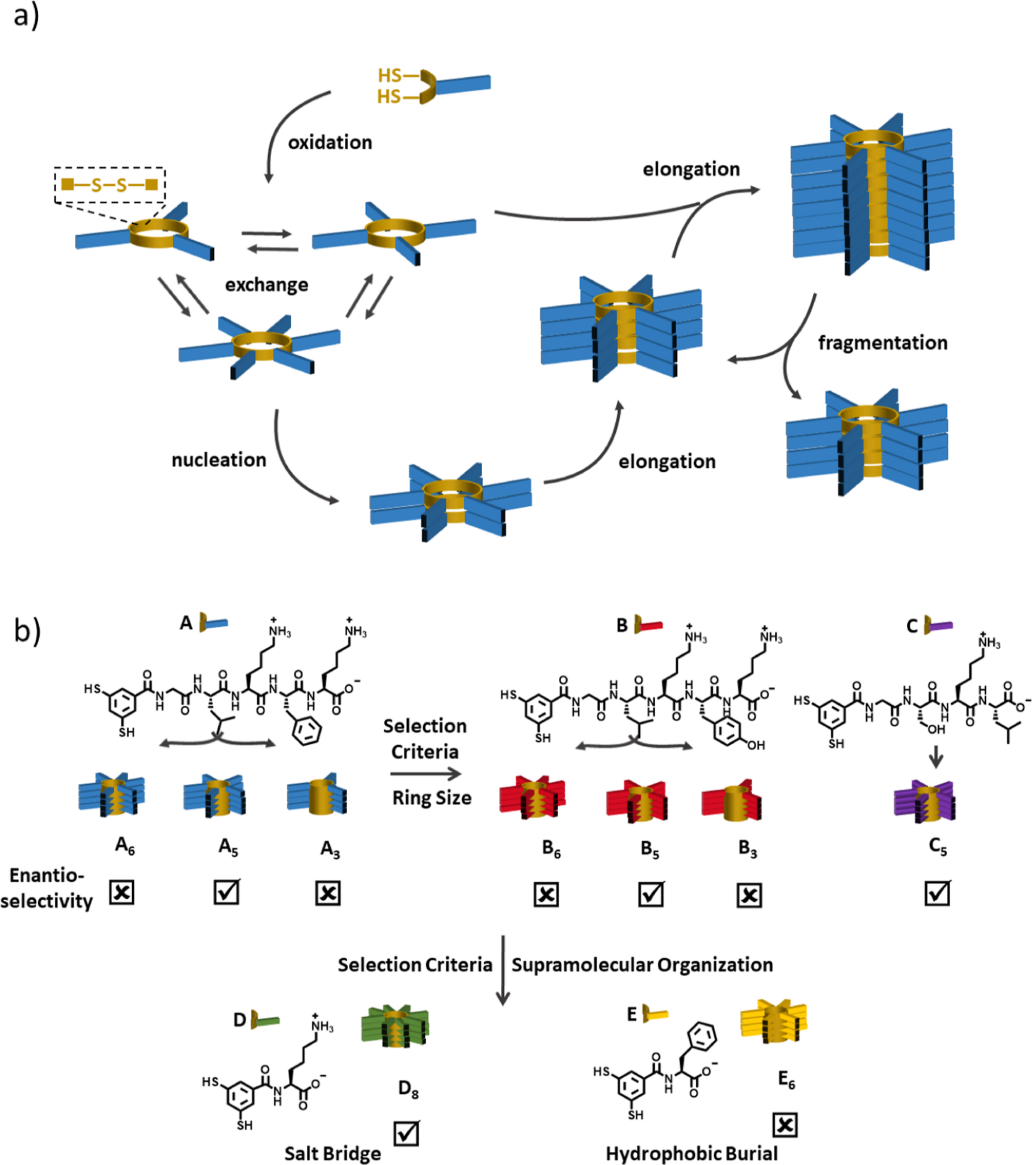
(a) Mechanism of replication. Building blocks consisting of a peptide strand (blue) attached to a dithiol aromatic group (yellow) oxidize in aqueous medium to form cyclic oligomers linked through disulfide bonds. The macrocycles constantly exchange in the presence of an unreacted monomer, giving a dynamic combinatorial library (DCL). One macrocycle of a specific size—here, a hexamer—is capable of stacking and forming a nucleus, which then grows into a fiber by incorporating material from smaller macrocycles (elongation). The fiber is held together by π–π-stacking of the disulfide-linked aromatic cores and β-sheets that form along the fiber axis. Mechanical stress, such as agitation with a magnetic stirrer bar, breaks the fibers into smaller fragments that continue to grow by elongation. (b) Replicators made from building block **A**, **B**, **C**, **D**, or **E** show enantioselectivity (i.e., incorporation of only one enantiomer into a single fiber) depending on their ring size and the possible interactions conferred by their respective peptide strand.

In previous work,^5^ using a dynamic combinatorial approach,^45^ we developed systems of self-replicators that form from building block **A** (Figure 1a) equipped with a relatively short (5-amino-acid) peptide sequence. The building block also contains an aromatic ring with two thiol groups for reversible thiol-disulfide chemistry. Oxidation of the thiols to disulfides in aqueous solution leads to the formation of interconverting macrocycles of different sizes. One of these macrocycles then stacks into fibers, driven by π–π-stacking of the cores and assembly of the peptides into β-sheets, and grows by feeding on the other macrocycles.^46^ Fiber breakage through mechanical stress (i.e., stirring) leads to an increase in the number of individual fibers and thus to exponential growth.^47^ In general, the size of the self-replicating macrocycle depends on the polarity of the side chains of the building blocks,^48^ solvent composition,^49^ and salt concentration.^50^ We previously found that hexamer replicators made from building block **A** (Figure 1b) show no enantioselectivity. When homochiral fibers are introduced in a solution containing racemic precursors, they grow incorporating the building blocks without any preference, albeit at a reduced rate compared to the rate at which homochiral fibers growth from precursors of the same chirality.^51^

We now report that a pentamer self-replicator made from the same building block **A** does exhibit enantioselectivity. Analysis of the mode of assembly led us to self-replicators made of other building blocks that are also enantioselective (Figure 1b). These homochiral fibers emerge through spontaneous self-assembly of simple building blocks, each bearing a side chain of five, four, or even only a single amino-acid residue. Precursors of the same handedness get incorporated preferentially: replicator **A**_**5**_ was observed to grow from a racemic pool of material with a high chiral fidelity, sorting (L)- and (D)-peptides into (L)- and (D)-replicators. A comparison of these systems with others that are not enantioselective points at the importance of directional interactions conferred by salt bridges, as opposed to non-directional hydrophobic burial, in enabling enantioselectivity.

## Results and Discussion

### **A**_**5**_: An Enantioselective Replicator

**Figure 2:**
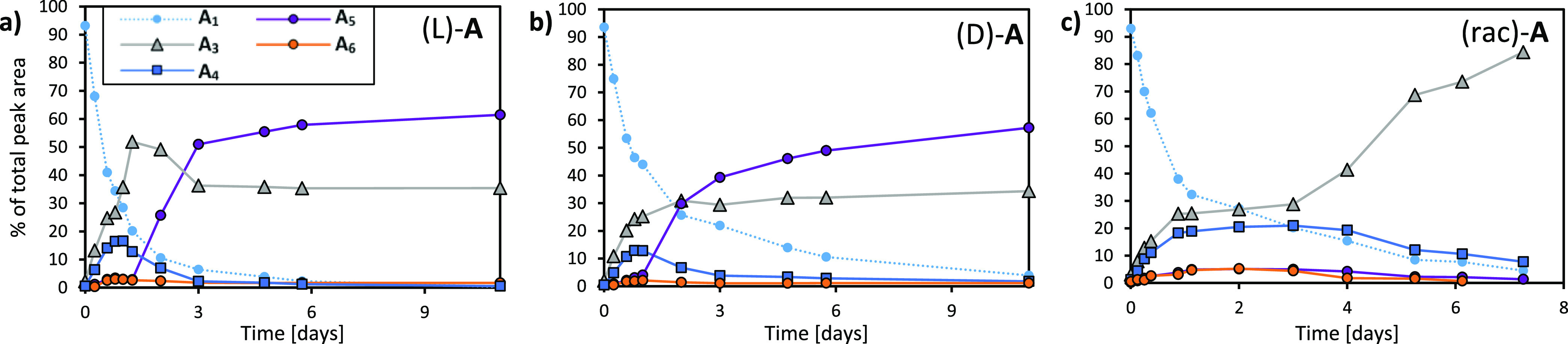
Emergence of **A**_**5**_/**A**_**3**_ replicators from (a) (L)-**A** and (b) (D)-**A** and emergence of racemic **A**_**3**_ replicator from (c) (rac)-**A**. The samples were prepared from 3.8 mM **A** in borate buffer (50 mM in B atoms), 4 M GuHCl, shaken at 1200 rpm at 20 °C, and their compositions were monitored by UPLC. Full experimental procedures can be found in the Methods section in the Supporting Information.

In previous work, we reported that building block (L)-**A** gives rise to a cyclic hexamer [(L)-**A**_**6**_] replicator in aqueous borate buffer, whereas the addition of guanidinium chloride (GuHCl) gives rise to a cyclic trimer [(L)-**A**_**3**_] replicator instead.^50^ Since then, we found that (L)-**A** can also form a pentamer replicator (L)-**A**_**5**_ (Figure 2a,b) under particular conditions: high **A** concentration (3.8 mM), >2 M GuHCl, 20 °C and agitation by shaking instead of stirring (a detailed discussion of the conditions for (L)-**A**_**5**_ emergence is provided in Supporting Information, Section 1.1.1). The composition of DCLs made from **A** was monitored by ultra-performance liquid chromatography (UPLC) at a wavelength at which the macrocycles have a similar molar absorptivity per building block unit^50,52,53^ (Supporting Information, Section 2.4), allowing for the direct comparison of UPLC peak areas. (L)-**A**_**5**_ was invariably accompanied by (L)-**A**_**3**_, usually in a ca. 60:35 **A**_**5**_/**A**_**3**_-ratio. This mixture was found to form laterally aggregated fibers as seen in transition electron microscopy (TEM; Supporting Information, Figure 18) images. Circular dichroism (CD; Supporting Information, Figure 24a) confirmed the presence of chiral, supramolecular aggregates, and a thioflavin T assay (ThT; Supporting Information, Figure 25a) gave results that are consistent with the presence of β-sheets. Interestingly, using the same conditions where (L)- and (D)-**A**_**5**_/**A**_**3**_ emerge, a library made from (rac)-**A** gives instead rise to **A**_**3**_ only, with some delay in its emergence (Figure 2c). The diastereomeric distribution of (rac)-**A**_**3**_ was investigated by growing it from a racemic (L)-A*/(D)-**A** library, where A* is isotopically labeled (containing a ^13^C_6_^15^N_1_-isotope labeled leucine residue). Use of this building block mixture allows to distinguish both enantiomers in mass spectrometry (MS), showing that **A**_**3**_, grown from racemic material, has a statistical distribution of diastereoisomers (Supporting Information, Figure 4) and thus does not chirally self-sort, similar to its hexameric counterpart **A**_**6**_.^51^ TEM micrographs of (rac)-**A**_**3**_ show laterally aggregated fibers that are similar in appearance to those of (L)- or (D)-**A**_**3**_ (Supporting Information, Figure 19).

**Figure 3:**
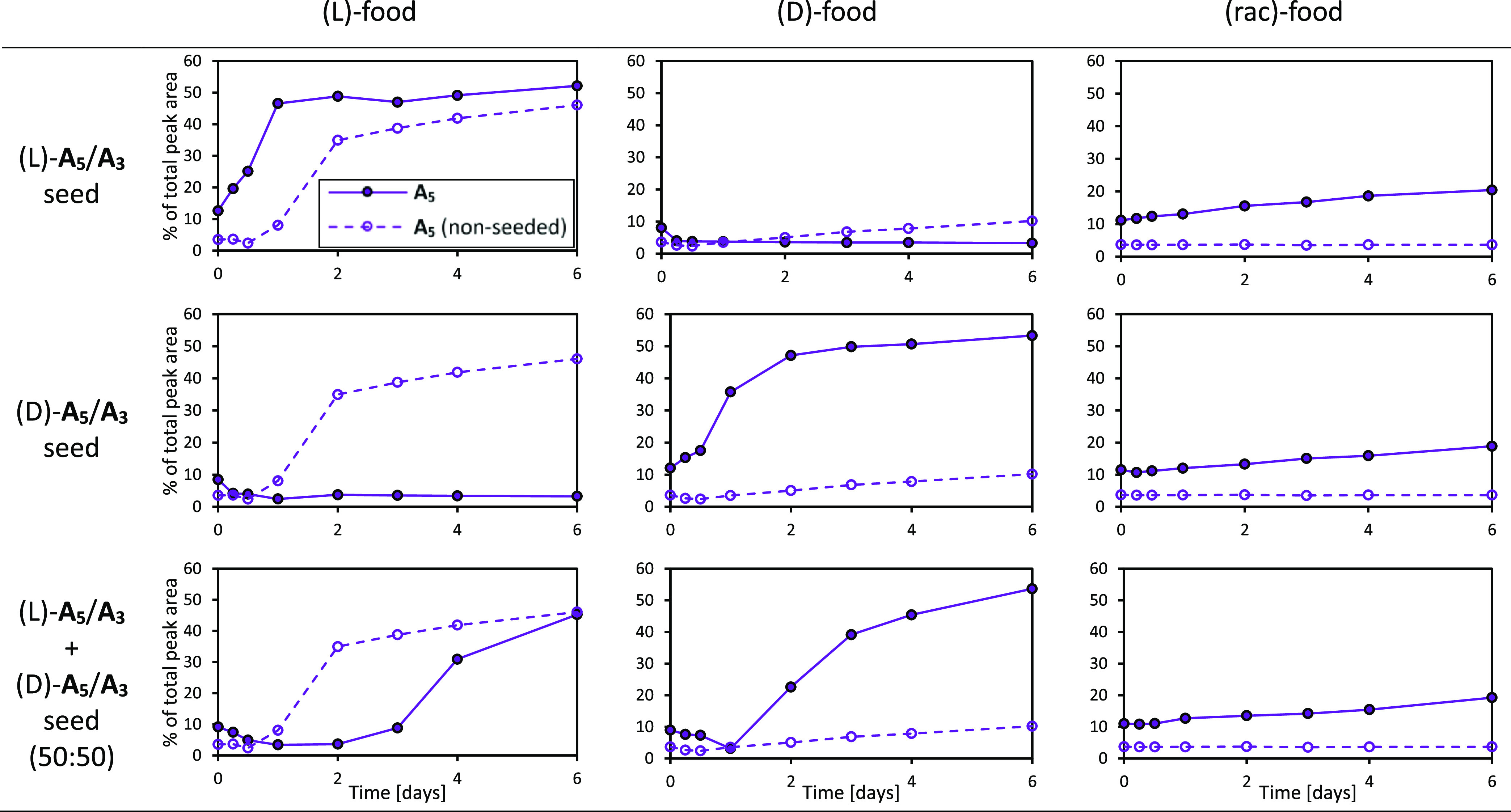
Change in the composition of DCLs containing preoxidized **A** (“food”; consisting mostly of unassembled **A**_**1**_, **A**_**3**_, and **A**_**4**_) to which an aliquot of a “seed” library (consisting of >90% **A**_**5**_/**A**_**3**_) was added at the beginning of the experiment (filled circles/full line). Open circles/dashed line: **A**_**5**_ evolution of control experiments with no seed added. Food and seed chiralities of the individual experiments are as indicated. Note that batch variabilities (cf. Supporting Information, Section 1.1.1) cause differences in **A**_**5**_ nucleation and growth, hence the differences between (L)- and (D)-control experiments and between (L)- and (D)-food in (L) + (D)-seeded reactions. Reaction conditions: 1.9 mM **A**, 80 mol % NaBO_3_, 15 mol % seed (resulting in ca. 10 mol % **A**_**5**_ and ca. 5 mol % **A**_**3**_), 4 M GuHCl, 1200 rpm shaking at 20 °C.

To verify that **A**_**5**_ is a replicator and that the absence of its emergence from (rac)-**A** is not due to mere nucleation issues, we performed seeding experiments by adding a small amount of assembled **A**_**5**_/**A**_**3**_ to a library of “food” (consisting mostly of unassembled **A**_**1**_, **A**_**3**_, and **A**_**4**_) and probed all possible combinations of chiral configurations of food and **A**_**5**_/**A**_**3**_ seed. The growth of **A**_**5**_ (Figure 3, filled circles) was monitored and compared to control libraries with no seed added (open circles). The results show an immediate, seed-induced acceleration of **A**_**5**_ growth when food and seed chirality match (L-food/L-seed; D-food/D-seed) and thus confirm that **A**_**5**_ is a self-replicator. In contrast, chirality-mismatched experiments show no seeding-induced **A**_**5**_ growth at all. Note that in the unseeded control experiments, we observed spontaneous nucleation of replicators from L-food, but not from D-food, which is attributed to batch-to-batch variation (the rate of nucleation of this general family of replicators often shows difficult-to-rationalize batch dependence, cf. Supporting Information, Section 1.1.1). Seeding a 50:50-mixture of (L)- and (D)-**A**_**5**_/**A**_**3**_ in either (L)- or (D)-food leads to a delayed **A**_**5**_ growth compared to the use of only the chirality-matched seed. In racemic food, all experiments show a slight rate enhancement regardless of the seed used, i.e., **A**_**5**_ slowly grows from racemic material even though it does not spontaneously nucleate under these conditions. Similar experiments were performed with assembled **A**_**3**_ as seed (Supporting Information, Figure 11) which showed a behavior that resembles that of **A**_**6**_:^51^ replicator growth was efficient with matched seed/food chirality. **A**_**3**_ also grows in racemic food, where growth accelerates upon addition of seed, which confirms that **A**_**3**_ self-replicates under these conditions. When food and seed chirality are mismatched, **A**_**3**_ growth is hampered.

**Figure 4:**
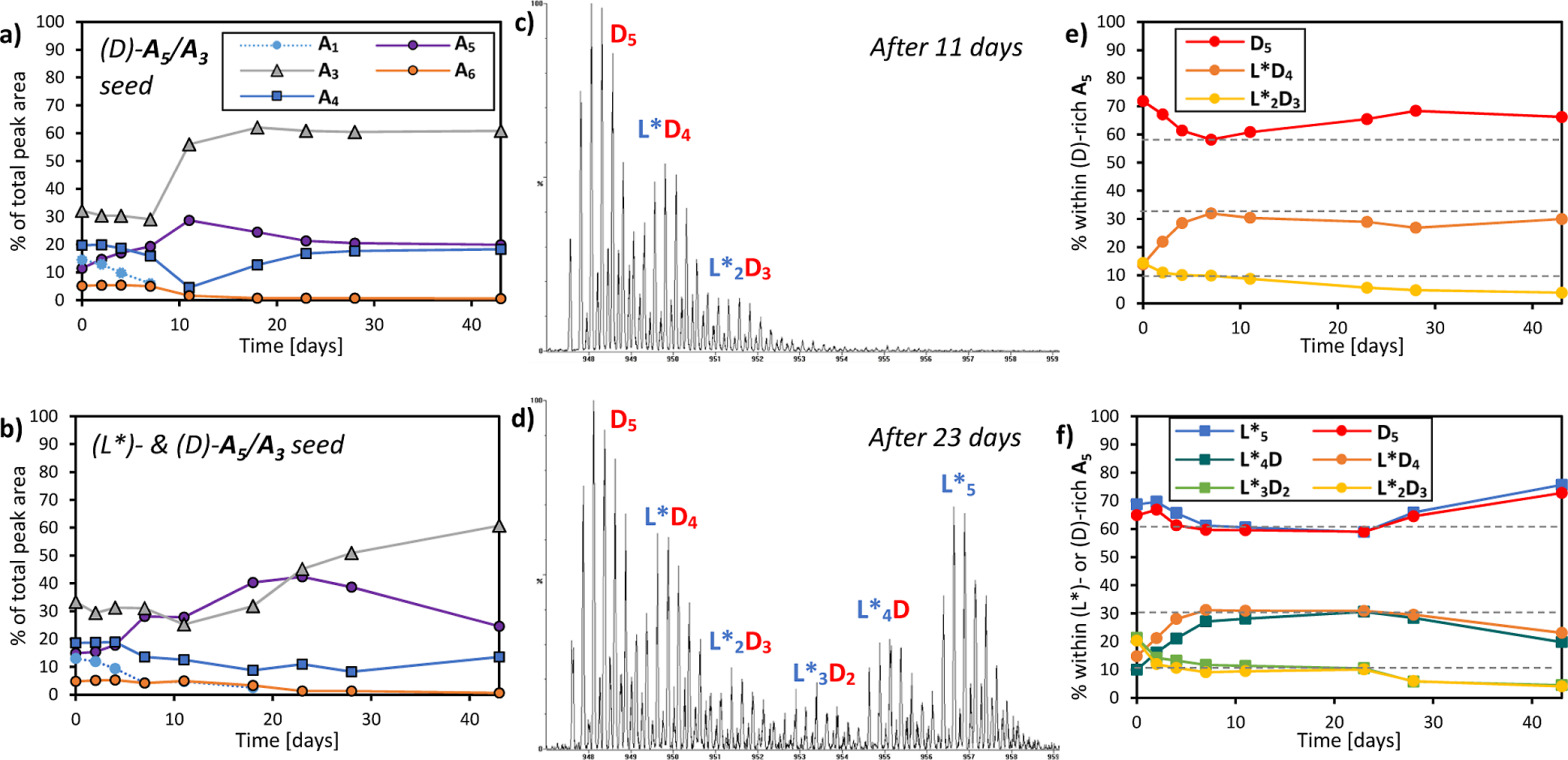
Change in product distribution of a DCL made from racemic (L)-A*/(D)-**A** building blocks, seeded with (a) (D)-**A**_**5**_/**A**_**3**_ or (b) both (L*)- and (D)-**A**_**5**_/**A**_**3**_ (3.8 mM total A*/**A**, 4 M GuHCl, 15 mol % per seed, borate buffer 50 mM in B atoms, shaking 1200 rpm at 20 °C); mass spectrum of **A**_**5**_ from the (c) (D)-seeded and (d) (L*)/(D)-seeded experiment (947–959 *m*/*z*, M + 4H^+^, recorded at the indicated time); and change in the relative amounts of diastereomers within **A**_**5**_ over time for the (e) (D)-seeded and (f) (L*)/(D)-seeded experiment.

Next, we probed whether **A**_**5**_ growing in (rac)-food incorporates both or mostly one of the available enantiomers. An aliquot of (D)-**A**_**5**_/**A**_**3**_ replicator was seeded in (L)-A*/(D)-**A** racemic food containing 4 M GuHCl and the change in library composition was monitored via UPLC and UPLC-MS. **A**_**5**_ grew from 11 to 29% of the total library material within 11 days (Figure 4a) after which the emergence of (rac)-**A**_**3**_ occurred (vide supra). The pentamer replicator incorporated only limited amounts of its mirror image building block (L)-A*, resulting in some (L*D_4_)- and (L_2_*D_3_)-**A**_**5**_. No pentamers with more than two units of (L)-A* were detected (Figure 4c). Using a fitting procedure (cf. Supporting Information, Section 2.5) with simulated mass spectra, we calculated the relative amounts of diastereomeric **A**_**5**_ macrocycles over time (Figure 4e). These converge to a ca. 60:30:10-ratio (D_5_:L*D_4_:L_2_*D_3_) within 11 days. Similar results were obtained when seeding both (L)-**A**_5_*/**A**_3_* and (D)-**A**_**5**_/**A**_**3**_ in (L)-A*/(D)-**A** racemic food: the total **A**_**5**_ grows to 42% of the library over 23 days (Figure 4b), until (rac)-**A**_**3**_ emerged. This mixture maintains a bimodal pattern in MS, indicative of chiral self-sorting (Figure 4d). Note that the L_2_D_3_ and L_3_D_2_ diastereomers, which would be the most abundant ones if building blocks would mix statistically, are the least populated. The diastereomeric distribution within each enantiomer replicator converges to the same ca. 60:30:10-ratio as seen before (Figure 4f). This results in up to 10% of the building blocks within (L)-**A**_5_* or (D)-**A**_**5**_ being of the “wrong” handedness. Altogether, this shows **A**_**5**_ to be an enantioselective replicator: it emerges only from enantiopure **A** and grows by incorporating material of its own handedness with relatively high fidelity. The same conclusion was reached upon repeating the experiment of Figure 4 at a lower GuHCl concentration (3 M instead of 4 M), which gave similar enantioselectivity, yet a lower extent of pentamer growth due to earlier emergence of the competing trimer replicator (Supporting Information, Figure 6).

We also monitored the extent of enantioselectivity in the growth of **A**_**3**_ during the experiment shown in Figure 4. Prior to seeding, **A**_**3**_ is mostly unassembled and exhibits a statistical distribution of stereoisomers (Supporting Information, Figure 5a). After seeding, it became progressively enriched in homochiral diastereomers (Supporting Information, Figure 5b,c,e,f). At this stage, the total amount of trimer hardly changes, most likely because the rate at which the amount of unassembled trimer in the “food” diminishes essentially equals the rate at which homochiral trimer fibers grow. Once trimer growth shows a sudden acceleration (day 7 in Figure 4a and day 11 in Figure 4b, which we attribute to the nucleation of racemic trimer replicator fibers) its distribution of stereoisomers reverted back to statistical (Supporting Information Figure 5d,g). (rac)-**A**_**3**_ growth leads to a partial consumption of **A**_**5**_, which reduces the extent to which the “wrong” enantiomer of **A** is incorporated from 10 to 7% (Figure 4c) or even 6% (Figure 4f) at day 43. This reduction is most likely a result of the fibers being consumed from their ends, which contain the largest fraction of “wrong” enantiomers as this fraction increased as the fibers grew.

Finally, we also analyzed the stereoisomers of **A**_**4**_. After (rac)-**A**_**3**_ emergence, the proportion of **A**_**4**_ remained remarkably stable (Figure 4b) or even increased after an initial drop (Figure 4a), while a decrease of **A**_**4**_ would be expected if it would only serve as “food” for the other replicating macrocycles. In the seeding experiments in Figure 4, **A**_**4**_ initially shows a statistical distribution of diastereomers (as expected from a non-assembled macrocycle) but later, after (rac)-**A**_**3**_ emergence, the racemic (L_2_*D_2_)-**A**_**4**_ is essentially the only diastereomer observed out of the five stereoisomers that **A**_**4**_ formed initially. MALDI-MS fragmentation indicates that this diastereomer is enriched in the alternating L–D–L–D isomer (Supporting Information, Figure 7). These observations suggest that **A**_**4**_ self-assembles in a highly diastereoselective manner. Further characterization and rationalization of this system is beyond the scope of this study but is underway in our group.

### B_5_ and C_5_ Are Also Enantioselective Self-Replicators

The results presented above raise the question what makes **A**_**5**_ enantioselective, as it differs only in ring size from the non-enantioselective **A**_**3**_ and **A**_**6**_—all these replicators consist of the same building blocks and bear the same functional groups. An investigation of other systems, selected for their ability to yield pentamer replicators, revealed that such a behavior is not restricted to DCLs made from building block **A**. Pentamer replicators **B**_**5**_ and **C**_**5**_ were also found to be enantioselective.

**Figure 5:**
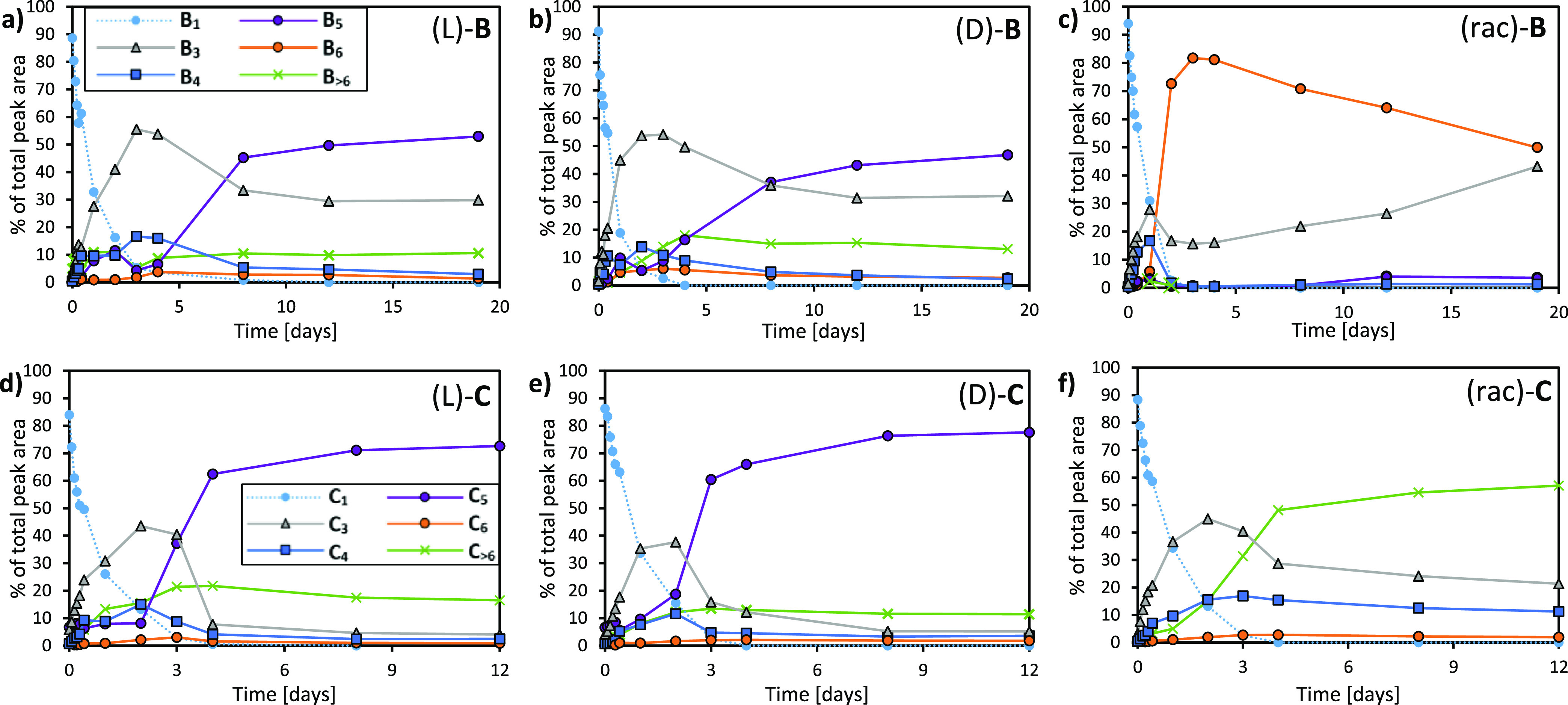
Change in compositions of DCLs obtained from building blocks (L)-, (D)-, and (rac)-**B** (panels a–c, respectively) and (L)-, (D)-, and (rac)-**C** (panels d–f, respectively). The libraries were prepared from 2.0 mM **B** or **C** in borate buffer (50 mM in B atoms), stirred at 1200 rpm at 45 °C and their compositions were monitored by UPLC.

Building block **B** is an analogue of **A** in which a single amino acid changed from phenylalanine to tyrosine (Figure 1b). It forms trimer (this work), octamer^54^ as well as pentamer^55^ replicators, depending on the reaction conditions. Here, we obtained **B**_**5**_ and **B**_**3**_ replicators from both **B** enantiomers in a similar fashion as described above for **A**_**5**_/**A**_**3**_ (Figure 5a,b), but without the need of adding GuHCl. However, a library of a (rac)-**B** subjected to the same reaction conditions gave rise to hexamer **B**_**6**_ instead, whose abundance decreases over time in favor of **B**_**3**_ (Figure 5c). A screening of seeding experiments similar to the experiments in Figure 3 (Supporting Information, Figure 12) gave comparable results: **B**_**5**_ shows a seeding effect only when seeded in same chirality food; seeding in opposite enantiomer food and racemic food does not induce any **B**_**5**_ growth.

Building block **C** bears a tetrapeptide (one unit shorter than **A** and **B**, and with a different peptide sequence; cf. Figure 1b) and was not found to give rise to any other replicator than pentamer **C**_**5**_. In conditions where **C**_**5**_ is obtained from either (L)-**C** or (D)**-C** (Figure 5d,e), a library of (rac)-**C** does not lead to the emergence of any specific macrocycle: a range of macrocycles ranging from 3- to 19mers is obtained instead (Figure 5c) and TEM shows amorphous aggregates instead of the fibers obtained for the pentamer-dominated samples (Supporting Information, Figure 21). Seeding experiments give results that match those obtained with the corresponding experiments conducted with replicator **B**_**5**_ (Supporting Information, Figure 13). In addition, seeding with the macrocycle mixture obtained from (rac)-**C** does not induce any change in the libraries. Thus, in DCLs made from (rac)-**C** replicator emergence does not take place under the conditions probed. Taken together, these results suggest a correlation between a ring size of five and enantioselectivity in self-replicators.

### Enantioselectivity is Likely to be Linked to Supramolecular Organization

**Figure 6:**
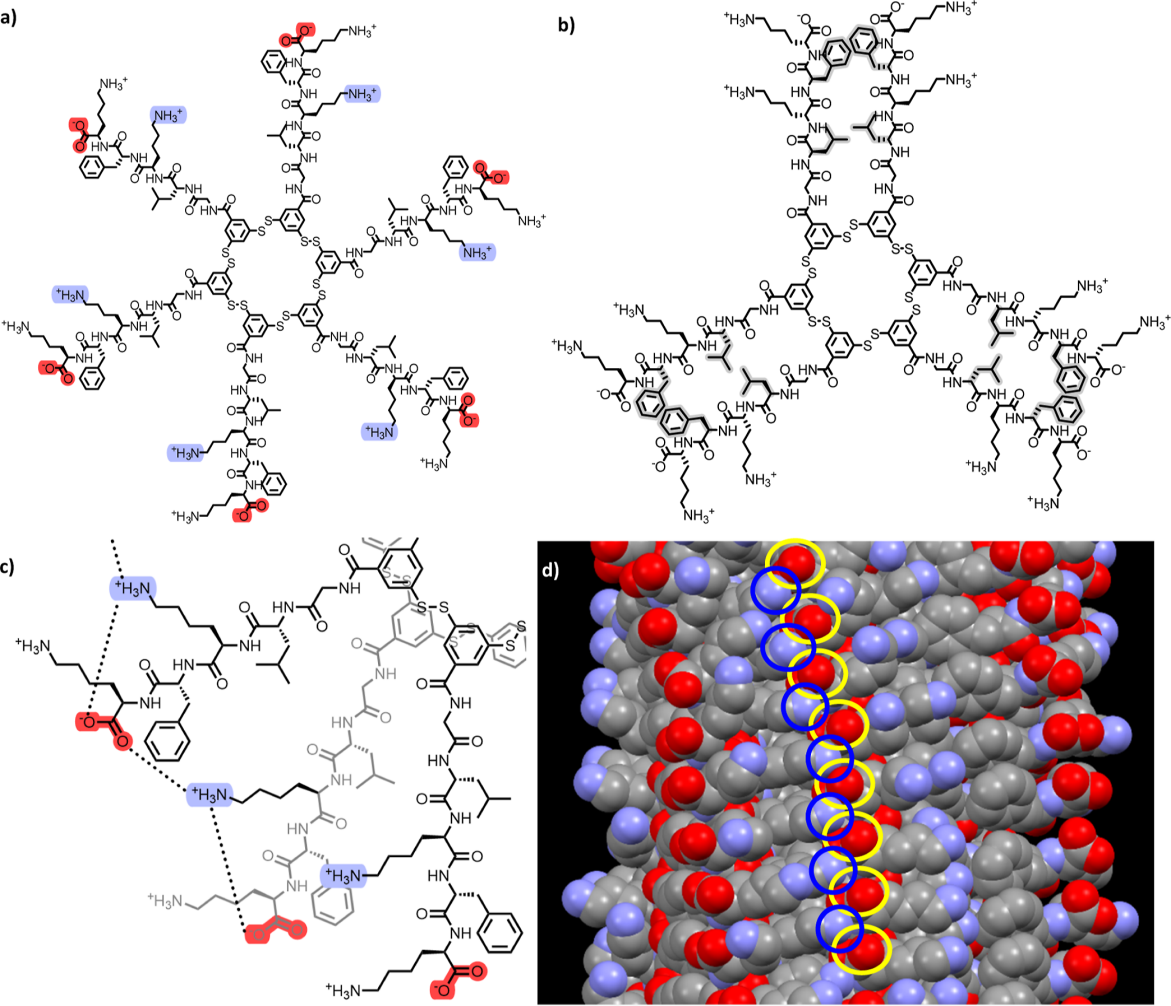
Schematic representations of **A**_**6**_ with a (a) cartwheel and (b) pairwise arrangement; (c) top-down view of an **A**_**6**_ fiber in cartwheel conformation, with focus on the salt bridges formed between inner lysine and carboxylate residues, (d) side-view on the MD simulation^56^ of an **A**_**6**_ fiber in cartwheel conformation with highlighted salt bridge-involved inner lysine residues (blue) and carboxylates (yellow); hydrogen atoms are omitted. For a clearer representation, the macrocycle core in (a,b) was drawn in an extended (approximately circular) conformation, suggesting space in the core; the core is actually collapsed in the MD simulations.

What could make the ring size of five special may be rationalized by revisiting structural studies we reported previously for **A**_**6**_ replicators. For **A**_**6**_ fibers, two possible configurations were identified using molecular dynamics (MD) simulations: “cartwheel” (Figure 6a) and “pairwise” (Figure 6b).^48^ In the cartwheel configurations, all peptides of a macrocycle are oriented in the same way (showing approximate C6 symmetry), whereas in the pairwise arrangement, the hydrophobic faces of the β-sheets pair up (yielding approximate C3 symmetry), which allows hydrophobic interactions within pairs of adjacent β-sheets. A peculiarity found in the cartwheel configuration is that the ammonium moieties of the inner lysine groups form salt bridges with two terminal carboxylate groups (Figure 6c). An important feature that was not emphasized in the original study is that these salt bridges lead to a specific three-dimensional structure which is feasible only if the assembly consists of homochiral building blocks. Each ammonium group interacts with two carboxylates (and vice-versa): one carboxylate at the end of its own peptide strand and another one belonging to the next macrocycle in the fiber (Figure 6c,d). Carboxylate and ammonium groups thus form a salt-bridge “chain” along the fiber axis. This chain of salt bridges requires a regular arrangement where all lysine residues point to the same direction along the fiber axis. Replacing a lysine by its enantiomer would cause the residue to point in the wrong direction, hampering salt bridge formation.

In contrast, no salt bridges or similar directional interactions were found in the “pairwise” configuration: the lysine residues simply point into water. Instead, the “pairwise” configuration is stabilized by hydrophobic interactions. We speculate that these hydrophobic interactions are more forgiving when it comes to the chirality of the peptides. Thus, the “pairwise” arrangement might have not the same constraints as the “cartwheel” form with respect to the homochirality of its constituents.

**Figure 7:**
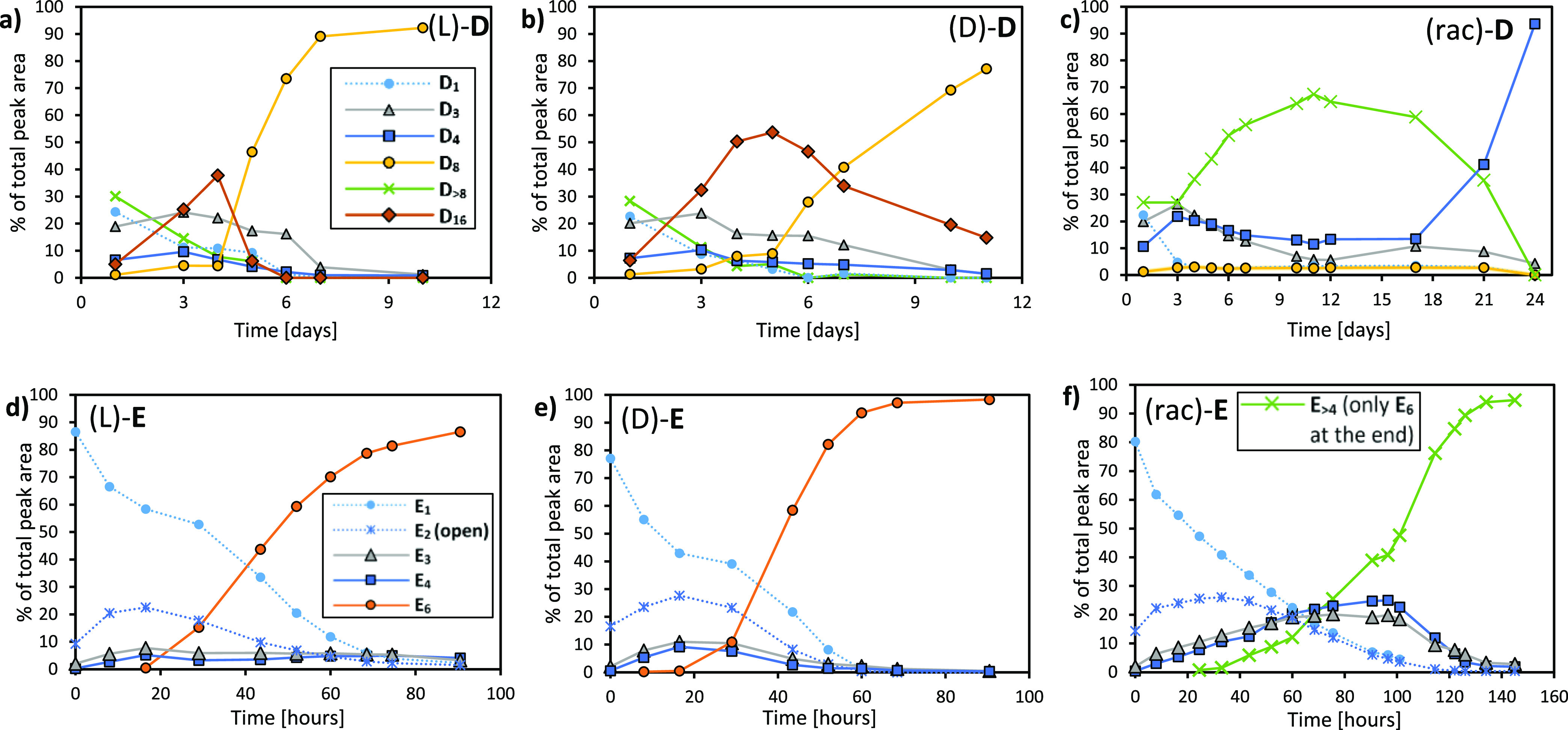
Changes in product distributions in DCLs obtained from building blocks (L)-, (D)-, and (rac)-**D** (panels a–c) and (L)-, (D)-, and (rac)-**E** (panels d–f). The libraries were prepared from 0.45 mM **D** in borate buffer (200 mM in B atoms) and stirred at 1200 rpm at rt or from 1.0 mM **E** in borate buffer (50 mM in B atoms) in the presence of 0.1 M GuHCl, stirred at 500 rpm at 40 °C in an automatic stirring device.^58^ Note that in panel c, the green trace for **D**_**>8**_ includes **D**_**16**_ which could not be quantified separately as it co-elutes with other large macrocycles. In panel f, **E**_**>4**_ was plotted instead of **E**_**6**_ because of overlap of mixed-chirality **E**_**6**_ macrocycles with other large macrocycles in the chromatograms (up to nonamers). The final composition consists of **E**_**6**_ only as verified by mass spectrometry.

It is difficult to verify these hypotheses via, e.g., spectroscopic analyses; spectra computed based on the MD trajectories of **A**_**6**_ fibers in the cartwheel and pairwise configurations showed only minor differences.^56^ However, some support for the above hypothesis is obtained from the behavior of DCLs made from the minimal building blocks **D** and **E** (Figure 1b). Their side chains consist of a single amino acid (lysine and phenylalanine, respectively), which constrains the way how they can interact within a fiber: apart from π–π stacking of the core and a single hydrogen bond, **D** can make a salt bridge while **E** can form hydrophobic interactions. Despite their minimal design, both building blocks were found to allow for fiber emergence: (L)- or (D)-**D** makes an octamer replicator **D**_**8**_ after having transiently formed the large macrocycle **D**_**16**_; **E** gives rise to hexamer replicator **E**_**6**_ in the presence of GuHCl (see Supporting Information, Section 1.1.4; in previous work we showed that polyamines can also promote the formation of this replicator^57^). We probed both systems for enantioselectivity and found replicator **D**_**8**_ to be enantioselective: we could only obtain **D**_**8**_ when starting from homochiral (L) or (D) building block (Figure 7a,b). When using (rac)-**D**, rather than producing **D**_**8**_ tetramer **D**_**4**_ formed eventually, after a long period where a variety of large macrocycles up to 20mers prevailed (Figure 7c). In contrast to **D**_**8**_, no fibers were observed in TEM for racemic **D**_**4**_ (Supporting Information, Figure 22). Seeding experiments (Supporting Information, Figure 14) show **D**_**8**_ to exhibit the same behavior as the pentamer replicators **B**_**5**_ and **C**_**5**_ and thus to be enantioselective. Growth of (rac)-**D**_**4**_ was also promoted by seeding, albeit only in racemic food.

In contrast, **E**_**6**_ fibers emerged from DCLs made from enantiopure as well as from racemic **E** (Figure 7a–c), which was confirmed by TEM, CD, and ThT assays (Supporting Information, Figures 23–25, respectively). Seeding effects confirmed (rac)-**E**_**6**_ to be a replicator (Supporting Information, Figure 15); moreover, seeding effects were observed for all possible seed/food chirality combinations (with some delay in replicator emergence for mismatched seed/food chiralities): **E**_**6**_ of any chirality promotes its own growth in food of any chirality. It is thus a non-enantioselective replicator, much like **A**_**6**_ and **A**_**3**_.

The fact that the octamer replicators made from **D**, where salt-bridge interactions can form, showed enantioselectivity, while the analogous system made from **E**, where hydrophobic interactions dominate but no salt bridges can form, shows no enantioselectivity, supports our hypothesis regarding the importance of salt-bridges in enantioselection.

These results are consistent with the notion that the cartwheel configuration is likely to be an inherently chiral sensitive structure, while the pairwise arrangement largely lacks such chiral sensitivity. This theory is in agreement with the results presented above with the pentamer systems made from building blocks **A**, **B**, and **C**. Pentamer replicators are unlikely to adopt a “pairwise” configuration since they have an odd-numbered ring size: one peptide strand would be left unpaired, with its hydrophobic residues not being shielded from water, which is likely to be an unfavorable arrangement. Therefore, the fact that **A**_**5**_, **B**_**5**_, and **C**_**5**_ are enantioselective would be consistent with these replicators forming a cartwheel assembly. On the other hand, **A**_**6**_, **B**_**6**_, and **E**_**6**_ can adopt the pairwise conformation (for **E**_**6**_, which lacks cationic side-chains forming a chain of salt bridges, the cartwheel configuration is even less favorable) and thus have the possibility to adopt a non chiral-sensitive structure, which allows them to incorporate both enantiomers and thus to emerge even from a racemic mixture of building blocks. The same reasoning may hold also for the tetrameric (rac)-**D**_**4**_ replicator.

However, ring size parity is not the only selection criteria determining which conformation can be adopted, as seen with **D**_**8**_. Eight is an even number; therefore, eight-membered macrocycles might, in principle, adopt either a cartwheel or a pairwise configuration. Since the pairwise configuration relies mostly on hydrophobic burial of peptide side-chains and building block **D** does not feature any hydrophobic amino acid, it is likely that **D**_**8**_ adopts the enantioselective cartwheel configuration. Indeed, **D**_**8**_ was found to be enantioselective. As for the trimeric **A**_**3**_ and **B**_**3**_ replicators: these are chirality-insensitive despite being odd-numbered, but consist also of relatively small macrocycles with a compact core. With a (supposedly) 120° angle between each peptide strand in the trimers, the distance between β-sheets may well be too large to form inter-strand interactions through a chain of salt bridges, thus preventing enantioselection from being manifested.

## Conclusions

In summary, we have found several self-replicators that are enantioselective, i.e., they do not readily emerge from racemic DCLs and do not grow efficiently in opposite-chirality food, but replicate fast when the chirality of the food matches their own chirality. Pentamer replicator **A**_**5**_ was even found to grow in racemic food, feeding mostly on material with a chirality that matches that of the replicator, with only 10% of incorporated building blocks being of opposite chirality. This behavior is in stark contrast to previous observations on the same class of replicators, which were relatively insensitive to the chirality of the precursors.^51^ This marked difference in enantioselectivity was found to correlate with the ring size of the replicator. While in all hexamer replicators we investigated so far no enantioselectivity is found, all pentamer replicators do show enantioselectivity, even when made from the same building block as the corresponding hexamer. This ability to be enantioselective is likely to be related to the supramolecular organization of the macrocycles within the fibers. Previous MD simulations indicated that self-replicators may assemble into fibers in two possible configurations: a “cartwheel” configuration, which features a chain of ammonium-carboxylate salt bridges, and a “pairwise” configuration, in which hydrophobic interactions drive adjacent β-sheets to pair up. It seems plausible that the salt-bridge chain imposes more severe demands on the chirality of the constituent building blocks than the hydrophobic interaction; i.e., the cartwheel configuration may well necessitate homochirality of its constituents. The pentamer replicators are likely to adopt the cartwheel conformation because, being odd-numbered, adopting a completely pairwise configuration is not possible. On the other hand, fibers of hexamer macrocycles can adopt the “pairwise” conformation which depends less on building block chirality. Therefore, these replicators can also emerge from racemic DCLs, giving rise to macrocycles with a statistical diastereomer distribution.

These results show that enantioselectivity in self-replication is not only possible for α-helical peptides^43^ but also extends to replicators featuring β-sheets. While enantioselectivity in previous work was observed for systems in which replicators have 31 chiral centers of identical chirality to start with,^43^ the present systems show signs of enantioselectivity starting from only eight chiral centers of matching chirality. Another difference with the α-helix-based replicators is the chiral bias in the precursors to the replicators. For the former system, even these precursors needed to be diastereomerically strongly enriched (having 14 and 17 stereocenters, all of the same chirality). The present system brings this number down substantially: enantioselection is seen with precursors that have four stereocenters of identical chirality (as for **A**), and signs of enantioselectivity have even been observed for **D**_**8**_, where building block **D** has only a single stereocenter.

These results show that enantioselective self-replicators are a means for achieving chiral amplification. It adds yet another life-like feature to our series of assembly-based self-replicators, for which we previously already demonstrated catalysis and proto-metabolism,^59,60^ parasitic/predatory behavior,^61^ diversification,^62^ stochasticity,^52^ and chemically fueled complexification.^50^

## Supporting Information Available

Conditions for **A**_**5**_ emergence; (L*D)-rac **A**_**3**_ and **A**_**4**_ in seeding experiments; **E**_**6**_ emergence and diastereomeric distribution; seeding experiments; DCL and sample preparation; UPLC, UPLC-MS, and MALDI-TOF analysis; UPLC chromatograms; mass spectra; ThT fluorescence assay; CD spectra; and TEM micrographs (PDF)
