# Supplementary material for: Enantioselective Self-Replicators
Source: J Am Chem Soc. 2023 Jul 24;145(30):16889–98. doi: 10.1021/jacs.3c05472 (PMC10401722; doi:10.1021/jacs.3c05472)
Supplement: Supplementary file 1 — ja3c05472_si_001.pdf [file ja3c05472_si_001.pdf]

Enantioselective Self-Replicators

Shuo Yang[a,b,c]†, Yannick Geiger[b]†, Marc Geerts[b], Marcel J. Eleveld[b], Armin Kiani[b] and Sijbren Otto[b*]

[a]State Key Laboratory of Metal Matrix Composites, School of Materials Science and Engineering, Shanghai Jiao Tong University, Shanghai 200240, P. R. China

[b]Centre for Systems Chemistry, Stratingh Institute, University of Groningen, 9747 AG Groningen, The Netherlands

[c]Zhangjiang Institute for Advanced Study (ZIAS), Shanghai Jiao Tong University, Shanghai 201203, P. R. China.

Supplementary Information

1 Supplementary Figures & Discussion 3

1.1 Replicator emergence & properties 3

1.1.1 **A_5** emergence: **A** & GuHCl concentration, temperature 3

1.1.2 (L*D)-rac **A_3** 5

1.1.3 Seeding experiments in (L*)-/(D)-**A** food 5

1.1.4 **E_6** emergence: GuHCl conc., statistical distribution of diastereoisomers 8

1.2 Seeding experiments 10

2 Materials and methods 15

2.1 General materials & methods 15

2.2 Library preparation 15

2.3 Seeding experiments 15

2.4 UPLC analysis 15

2.5 UPLC-MS analysis & data treatment 18

2.6 MALDI-TOF analysis 19

2.7 Transmission Electron Microscopy (TEM) 20

2.8 Circular Dichroism (CD) 20

2.9 Thioflavine T (ThT) fluorescence 20

3 Characterization data 21

3.1 TEM 21

3.2 Circular Dichroism 27

3.3 Thioflavin T assays 28

3.4 UPLC and UPLC-MS Analyses 30

3.4.1 Building block **A** 30

3.4.2 Building block **B** 35

3.4.3 Building block **C** 39

3.4.4 Building block **D** 45

3.4.5 Building block **E** 49
3.5 MALDI-TOF 55
4 References 55

1 Supplementary Figures & Discussion

1.1 Replicator emergence & properties

1.1.1 A_5 emergence: A & GuHCl concentration, temperature

We have noticed that a careful control of the reaction conditions is needed to obtain a reproducible emergence of $\mathbf{A_5}$/$\mathbf{A_3}$. Shaking instead of stirring is important, as well as a concentration in **A** of 1.9 - 3.8 mM. Lower concentrations lead to a lower $\mathbf{A_5}$/$\mathbf{A_3}$-ratio (ca. 50/50 instead of 60/35 at [**A**] = 1.0 mM, cf. Supplementary Figure 1a). Maintaining the reaction temperature at 20 °C was found to be crucial to obtain a consistent emergence of $\mathbf{A_5}$/$\mathbf{A_3}$ at GuHCl concentrations of 2-5 M (Supplementary Figure 2). Even at only 5 °C higher we started to observe $\mathbf{A_5}$/$\mathbf{A_3}$-ratios that varied from experiment to experiment. At 45 °C we observed the (co-)emergence of hexamer $\mathbf{A_6}$ up to a concentration of 4 M GuHCl - which leads to the coexistence of three different replicators, $\mathbf{A_6}$, $\mathbf{A_5}$ and $\mathbf{A_3}$ (Supplementary Figure 3). $\mathbf{A_5}$/$\mathbf{A_3}$ is obtained unaccompanied by $\mathbf{A_6}$ at 5 M GuHCl, but at a low $\mathbf{A_5}$/$\mathbf{A_3}$ ratio of ca. 50/50.

In addition, we have experienced that batch variabilities can have a large impact on $\mathbf{A_5}$ emergence. All results shown in this study were obtained from a single batch of (L)-**A** and (D)-**A** purchased both from Cambridge peptides. However, earlier studies with different batches (from the same but also from other vendors) found no $\mathbf{A_5}$ emergence under any conditions, or only at 2.0-2.5 M GuHCl ([**A**] = 3.0 mM), giving $\mathbf{A_3}$ only, while higher GuHCl concentrations yielded only unassembled $\mathbf{A_4}$ and $\mathbf{A_3}$ macrocycles. The isotope labeled (L)-$\mathbf{A^*_5}$/$\mathbf{A^*_3}$ replicator was grown from 3.8 mM (L)-**A*** and 3 M GuHCl at 20 °C; 4 M GuHCl lead to the emergence of (L)-$\mathbf{A^*_3}$ only.

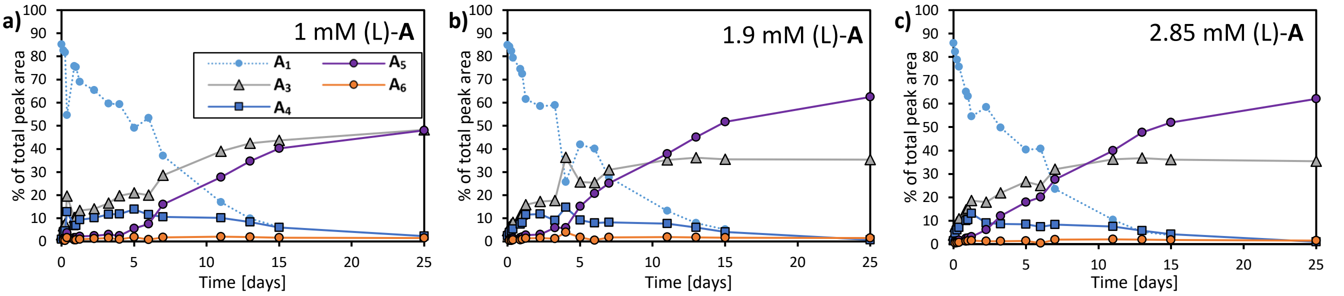

Supplementary Figure 1. *Evolution of DCLs at different (L)-**A** concentrations. Reaction conditions: **A** in borate buffer (50 mM in B atoms), 4 M GuHCl, shaken at 1200 rpm at 20 °C.*

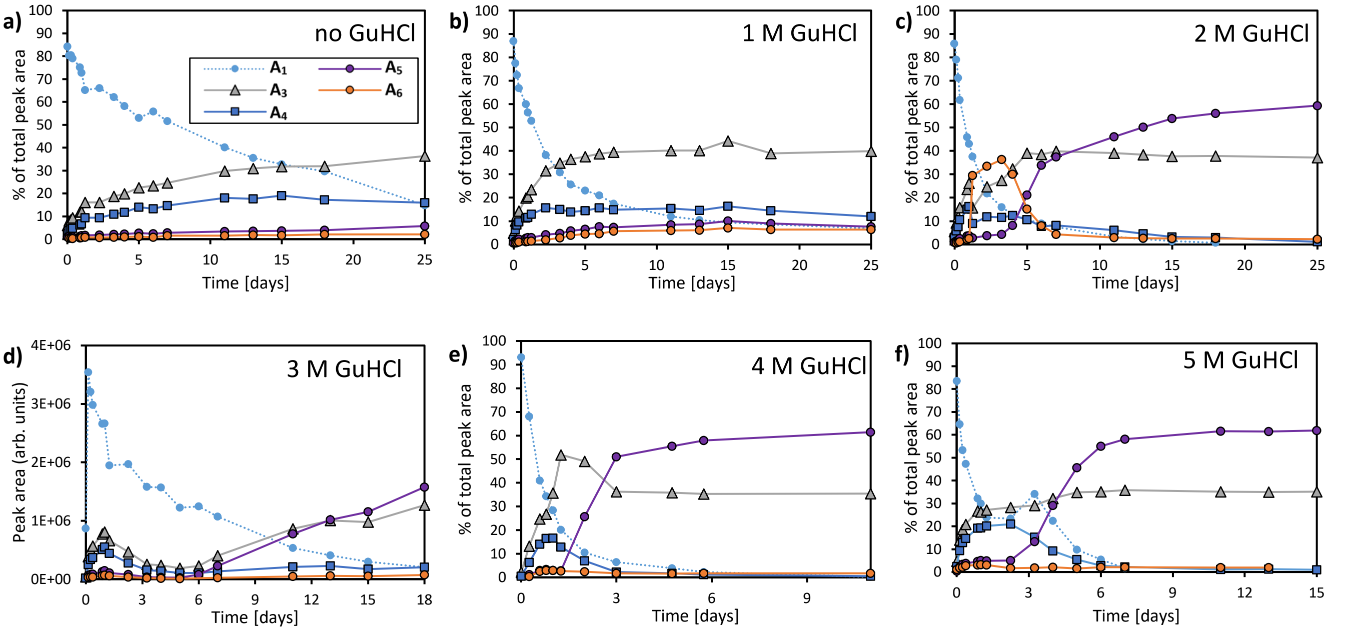

Supplementary Figure 2. *DCLs of (L)-**A** at 20 °C and various GuHCl concentrations. Reaction conditions: 3.8 mM **A** in borate buffer (50 mM in B atoms), shaken at 1200 rpm at 20 °C (same conditions as used in Figure 2 in the main text; panel (e) corresponds to Figure 2a). Note: in panel (d) we observed some loss of total peak area loss due to precipitation of unassembled $\mathbf{A_3}$ and $\mathbf{A_4}$ between day 1 and day 5, therefore the macrocycle abundance is plotted as absolute peak area instead as percentage of total peak area to prevent curve distortion of $\mathbf{A_1}$. On day 18, $\mathbf{A_5}$ represents 46% and $\mathbf{A_3}$ 37% of the total peak area.*

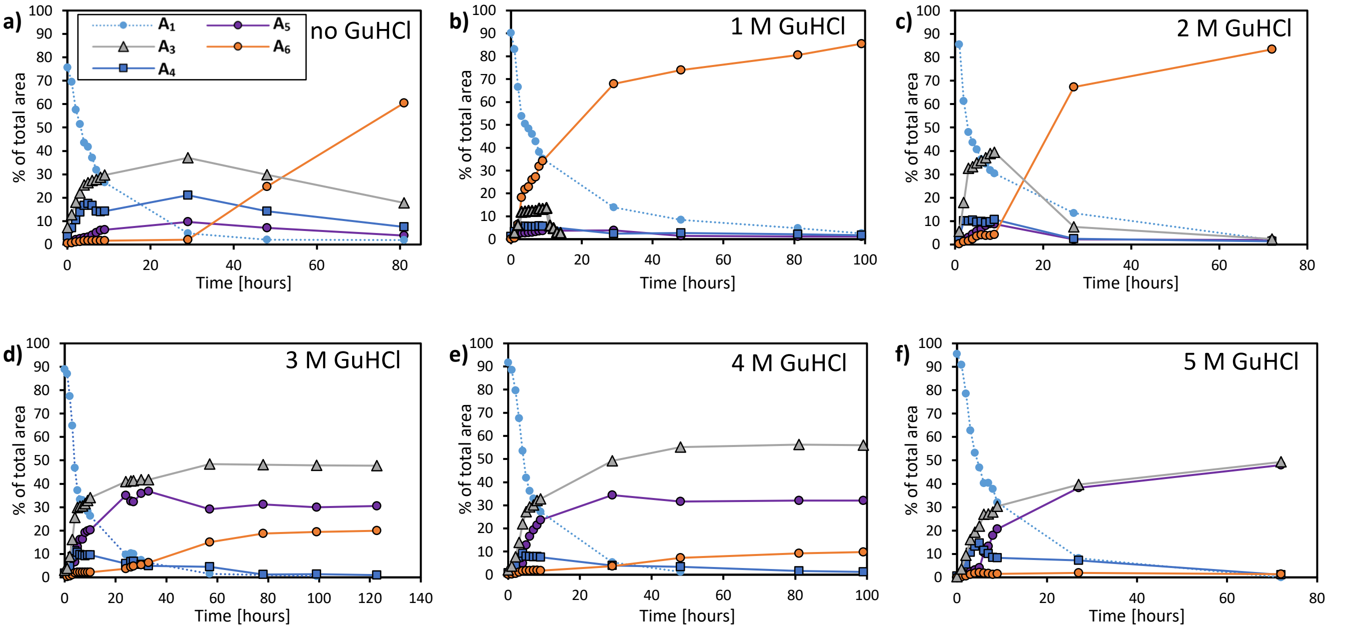

***Supplementary Figure 3**. DCLs of (L)-**A** at 45 °C and various GuHCl concentrations. Reaction conditions: 3.8 mM **A** in borate buffer (50 mM in B atoms), shaken at 1200 rpm.*

1.1.2 (L*D)-rac A_3

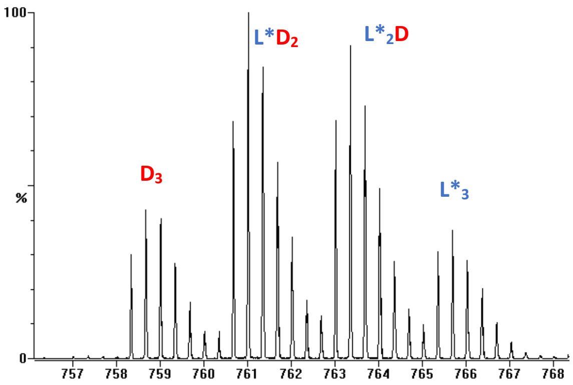

Supplementary Figure 4. *Mass spectrum (M + 3H⁺) of (rac)-**A₃** after 3 days (when it corresponds to 95% of total peak area) grown from (L*D)-rac **A** (3. 8 mM, 3 M GuHCl, 1200 rpm stirring at 45 °C). (rac)-**A₃** exhibits the 1:3:3:1 diastereomeric ratio expected for a statistical distribution.*

1.1.3 Seeding experiments in (L*)-/(D)-A food

Before seeding, the $\mathbf{A_3}$ macrocycles are unassembled and exhibit a statistical distribution (Supplementary Figure 5a). In the (D)-seeded experiment (cf. Figure 4a, main text) the total amount of $\mathbf{A_3}$ remains constant through the first seven days but the proportion of homochiral species (D_3)-$\mathbf{A_3}$ increases (compare Supplementary Figures 5b+c), indicating that the (D_3)-$\mathbf{A_3}$ replicator seed grows. The proportion in (L^*_3)-$\mathbf{A_3}$ also increases somewhat, probably due to a statistical effect – as (D)-$\mathbf{A_5}$/$\mathbf{A_3}$ consumes mostly (D)-food, unassembled macrocycles get enriched in the L*-enantiomer and the proportion of (L^*_3)-$\mathbf{A_3}$ increases. The sudden increase of overall $\mathbf{A_3}$ at day 11 is accompanied by an increased proportion of heterochiral (L^*D_2)- and (L^*_2D)-$\mathbf{A_3}$, indicating the emergence of (rac)-$\mathbf{A_3}$ (Supplementary Figure 5d). Similar behavior was observed in the (L*)-/(D)-seeded experiment (Supplementary Figure 5e-g; cf. also main text, Figure 4b).

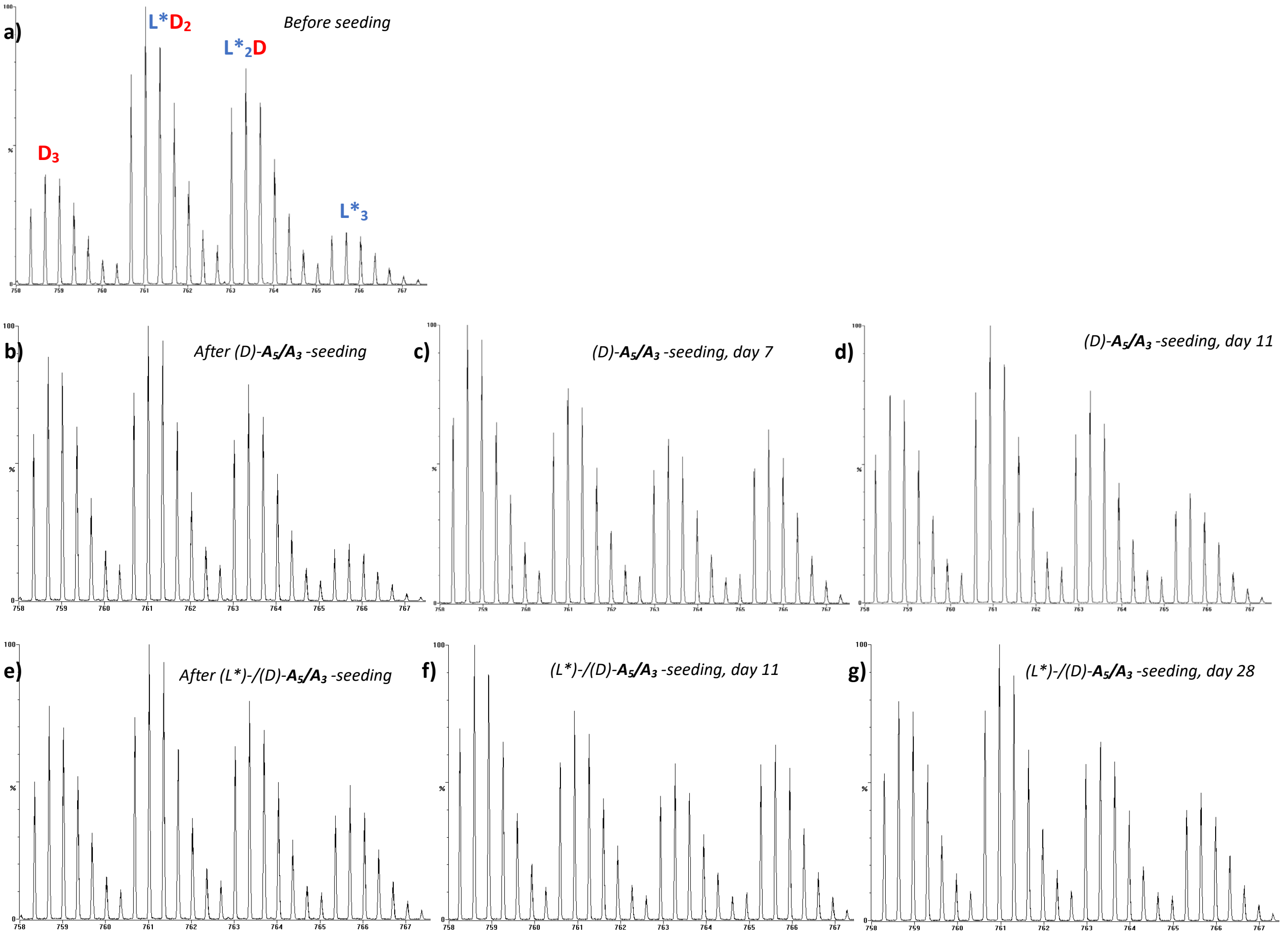

Supplementary Figure 5. *Mass spectra of* ***A*****$_3$** *(758 to 767 m/z, M + 3H$^+$) in the (D)-**A**$_5$/**A**$_3$-seeded experiment (cf. main text, Figure 4a) a) before seeding, b) right after (D)-**A**$_5$/**A**$_3$ seeding, c) after 7 days, d) after 11 days; in the (L*)-/(D)-**A**$_5$/**A**$_3$-seeded experiment (cf. main text, Figure 4b) e) right after (L*)-/(D)-**A**$_5$/**A**$_3$ seeding, c) after 1 day, d) after 28 days.*

In (D)- and (L*)-/(D)-**A**$_5$/**A**$_3$ seeding experiments with 3 M GuHCl, (rac)-**A**$_3$ emerged within the first 2 days and limited further **A**$_5$ growth (Supplementary Figure 6a and b) as compared to the 4 M GuHCl-experiments in the main text (Figure 4). The diastereomeric ratios also evolved only marginally after two days (Supplementary Figures 6c and d). The error (percentage of wrong enantiomer within an **A**$_5$ stack) was found to be ca. 5% in the (D)- (after 7 days) and ca. 6% (after 11 days) in the (L*)-/(D)-**A**$_5$/**A**$_3$ seeded experiments.

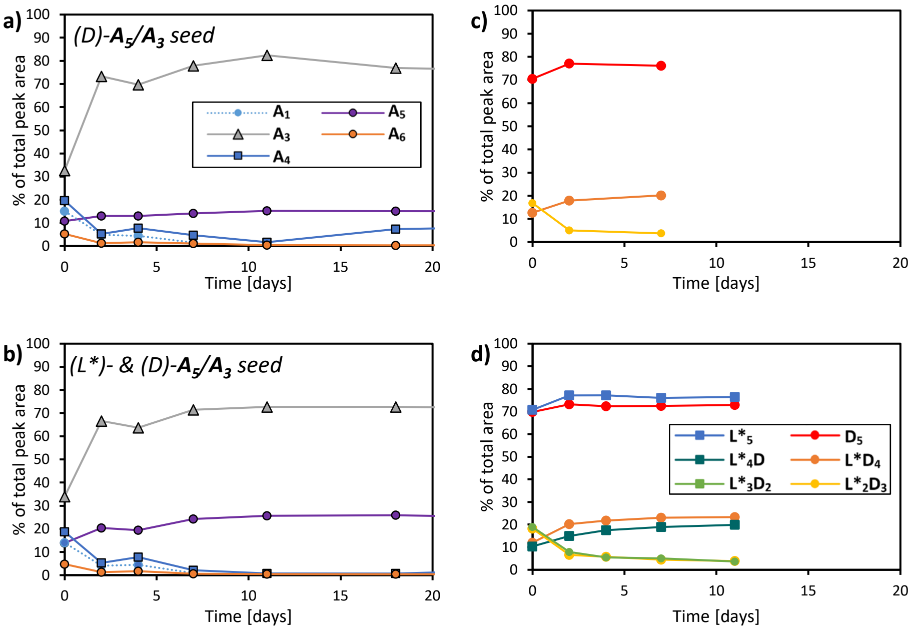

Supplementary Figure 6. *Change in composition of DCLs made from racemic (L)-**A***/(D)-**A** seeded with a) (D)-**A_5/A_3**, b) both (L*)- and (D)-**A_5/A_3** (3.8 mM total **A*/A**, 15 mol% per seed, 3 M GuHCl, borate buffer 50 mM in B atoms, shaken 1200 rpm at 20 °C); change of the relative amounts of diastereomers within (D)- and/or (L*)-**A_5** over time for the c) (D)-seeded, d) (L*)-/(D)-seeded experiment. Note that this experiment differs from that in Figure 4 in the main text in that a lower GuHCl concentration was used (3 M instead of 4M).*

Initially, the unassembled **A_4** macrocycle also shows a statistical distribution of diastereoisomers (Supplementary Figure 7a), but at a later stage only the *meso* ($L^*_2D_2$)-diastereoisomer is observed (Supplementary Figure 7b). This tetramer macrocycle can exist as two constitutional isomers, blockwise (L*L*DD) and alternating (L*DL*D), which occur in a 2:1 ratio when statistical (Supplementary Figure 7c). To determine the blockwise/alternating ratio we isolated an aliquot of ($L^*_2D_2$)-**A_4** via UPLC and analyzed it using Matrix Assisted Laser Desorption Ionization-Time of Flight (MALDI-TOF) Mass Spectroscopy. This technique partly fragments the analyte by homolytic scission of the disulfide bonds, giving rise also to trimers and dimers (cf. Supplementary Figure 7d; the full MALDI-TOF spectrum can be found in Supplementary Figure 72). The alternating isomer should only yield L*D dimer fragments, whereas the blockwise isomer should give rise to (L^*_2):(L*D):(D_2) in a 1:2:1 ratio. Taking into account the 2:1 blockwise/alternating-ratio in a statistical distribution (no preference for alternating or blockwise) the overall (L^*_2):(L*D):(D_2)-ratio should be 17:66:17 (Supplementary Figure 7e, blue bars). The ratio found using MALDI for ($L^*_2D_2$)-**A_4** (Supplementary Figure 7e, orange bars) is 6:84:10. The distribution is clearly non-statistical and enriched in the alternating isomer.

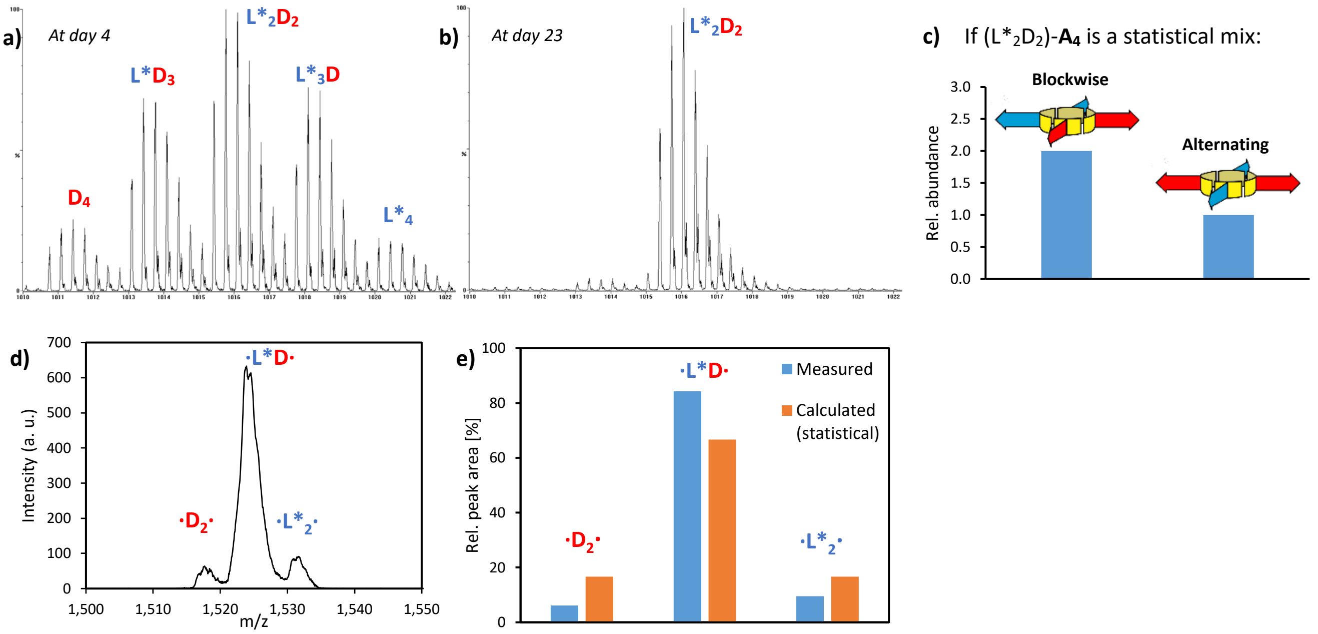

Supplementary Figure 7. *Mass spectra of* ***A***$_4$ *(1010 to 1022 m/z, M + 3H$^+$) in the (D)-**A**$_5$/**A**$_3$-seeded experiment (cf. main text, Figure 4a) a) after 4 days, b) after 23 days, c) relative abundance of blockwise and alternating isomers of (L*$_2$D$_2$)-**A**$_4$, d) MALDI-TOF mass spectrum of the **A**$_2$ fragment of (L*$_2$D$_2$)-**A**$_4$ (M + H$^+$), e) calculated (orange, statistical ratio assumed) and measured (blue, from MALDI-TOF peak integration) relative abundances of the three isomeric ·**A**$_2$· fragments in (L*$_2$D$_2$)-**A**$_4$.*

1.1.4 E$_6$ emergence: GuHCl conc., statistical distribution of diastereoisomers

In the original study of DCLs made from **E**, polyamines such as spermine and 1,6-hexamethylenediamine were found to be necessary for **E**$_6$ emergence; a diamine such as DABCO did not give rise to **E**$_6$ under otherwise similar conditions.[1] We found that GuHCl also promotes **E**$_6$ emergence and growth, even though only at higher concentrations. Whereas a substoichiometric amount of polyamine was sufficient (20 mol% for 1.0 or 3.0 mM **E**), we observe **E**$_6$ emergence only at GuHCl concentrations higher than 3.0 mM (Supplementary Figure 8). Concentrations in the molar range are also tolerated (Supplementary Figure 9), 2.0 M leading to a faster **E**$_6$ growth than 1.0 M GuHCl.
In general, (rac)-**E**$_6$ grows slower than (L)- or (D)-**E**$_6$ and its emergence is more demanding concerning the reaction conditions. We observed (rac)-**E**$_6$ to emerge at 45 °C within 24h but not at rt over a week when using 0.10 mM GuHCl, 1.0 mM (rac)-**E**, 80% preoxidized with $NaBO_3$, borate buffer (50 mM in B atoms) and 1200 rpm stirring. With enantiopure building block, **E**$_6$ is readily obtained under both conditions.

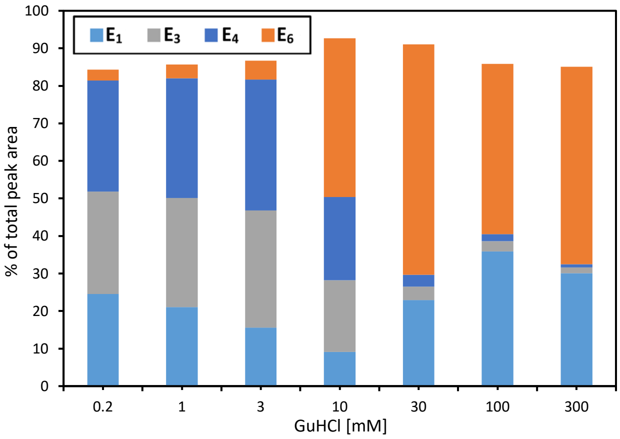

***Supplementary Figure 8**. Macrocycle distribution of DCLs of (L)-**E** (1.0 mM) after 3 days stirring at 45 °C and 1200 rpm, depending on the concentration of GuHCl. $\mathbf{E_6}$ emerges only at GuHCl concentrations above 3 mM, otherwise unassembled $\mathbf{E_3}$ and $\mathbf{E_4}$ prevail.*

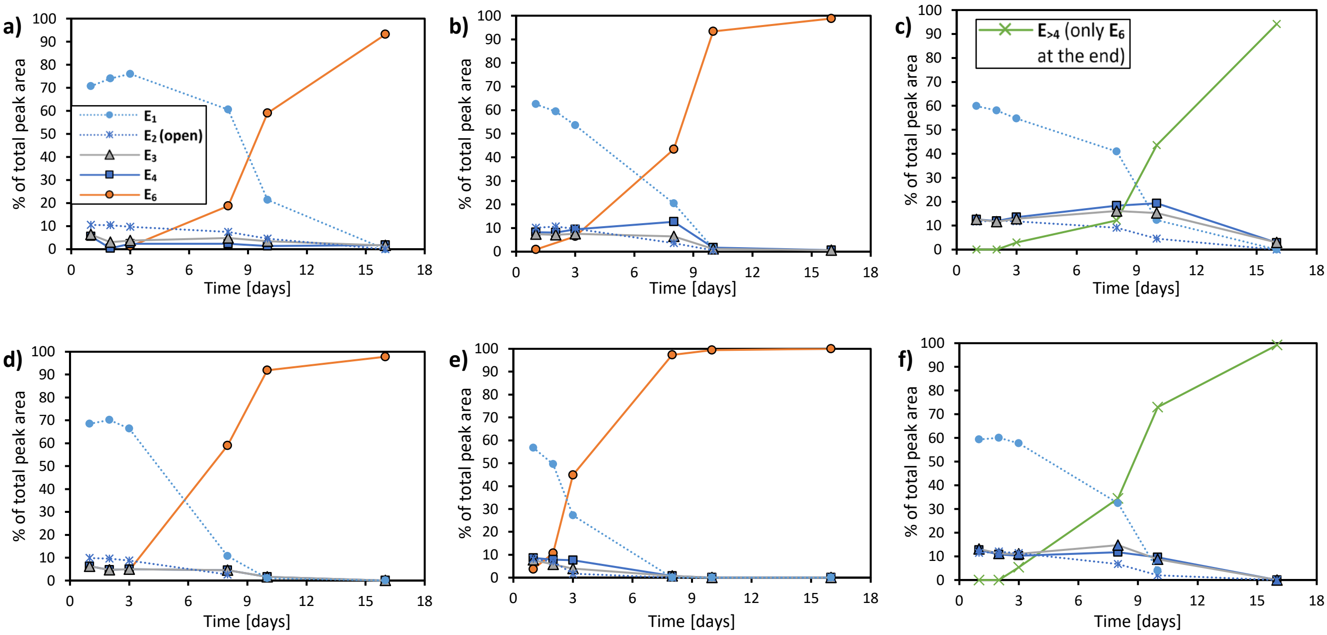

***Supplementary Figure 9.** Change in composition of DCLs made from (L)-, (D)- and (rac)-**E** at rt with 1.0 M (a, b and c, respectively) or 2.0 M GuHCl (d, e and f, respectively). Reaction conditions: 1.0 mM **E** in borate buffer (50 mM in B atoms), stirred at 1200 rpm at rt.*

The different diastereomers of (rac)-**E** macrocycles separate well by UPLC and allow analysis of their distribution up to $\mathbf{E_4}$ (larger macrocycles overlap and do not allow quantification). We expect two diastereomers for $\mathbf{E_3}$(homo- and heterochiral in a 1:3 ratio, Supplementary Figure 10a) and four for $\mathbf{E_4}$ (homo- and heterochiral, meso blockwise and meso alternating, in a 1:3:2:1 ratio). Indeed, we observe a ca. 1:3 ratio for the $\mathbf{E_3}$ species (Supplementary Figure 10b) and a ca. 1:3:2:1-ratio for the $\mathbf{E_4}$ species (Supplementary Figure 10c) in an experiment from (rac)-**E** without replicator emergence. We can thus reasonably assume that unassembled **E** macrocycles exist as a statistical diastereomer distribution. This further allows us also to assign the different $\mathbf{E_4}$ traces to the different isomers, from which otherwise only the homochiral isomer would be known from comparison with (L)- or (D)-**E** experiments. For assembled (rac)-$\mathbf{E_6}$ we could not assess the diastereomer distribution due to extensive peak tailing and overlap of the non-homochiral hexamers (cf. Supplementary Figure 62). However, the overall shape and number of distinguishable peaks (7 of 8 possible isomers) allow to exclude selectivity for one or few particular $\mathbf{E_6}$ diastereomers.

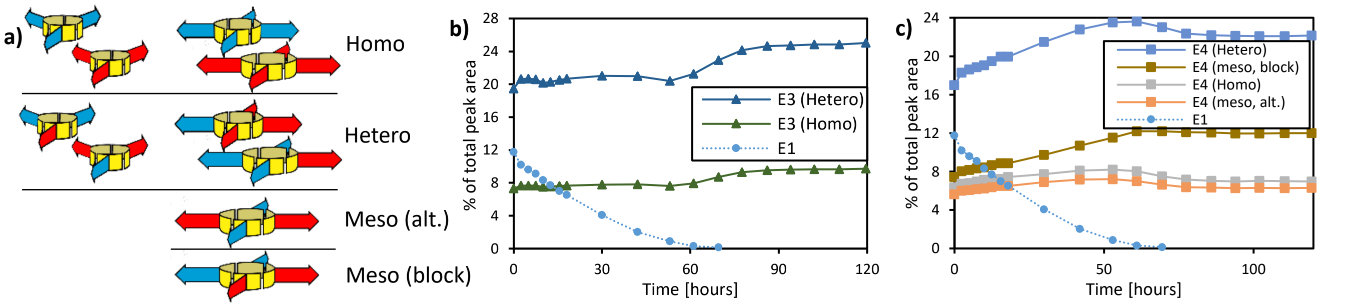

Supplementary Figure 10. *Change in the ratio of individual diastereo- and configurational isomers of a) (rac)-**E₃** and b) (rac)-**E₄** from the unseeded control experiment with (rac)-**E** food (cf. seeding experiments with **E** in the following section).*

1.2 Seeding experiments

This section shows further seeding experiments similar to the ones in Figure 3 in the main text. Please note that batch variabilities (cf. section 1.1.1) can cause differences in replicator nucleation and growth, hence the differences between (L)- and (D)-seeded or control experiments in some cases. In systems containing an additional replicator (i.e the trimer in **A**$_5$/**A**$_3$ and **B**$_5$/**B**$_3$) its growth affects the food availability and thus also pentamer growth to some degree, which can also cause differences between experiments.

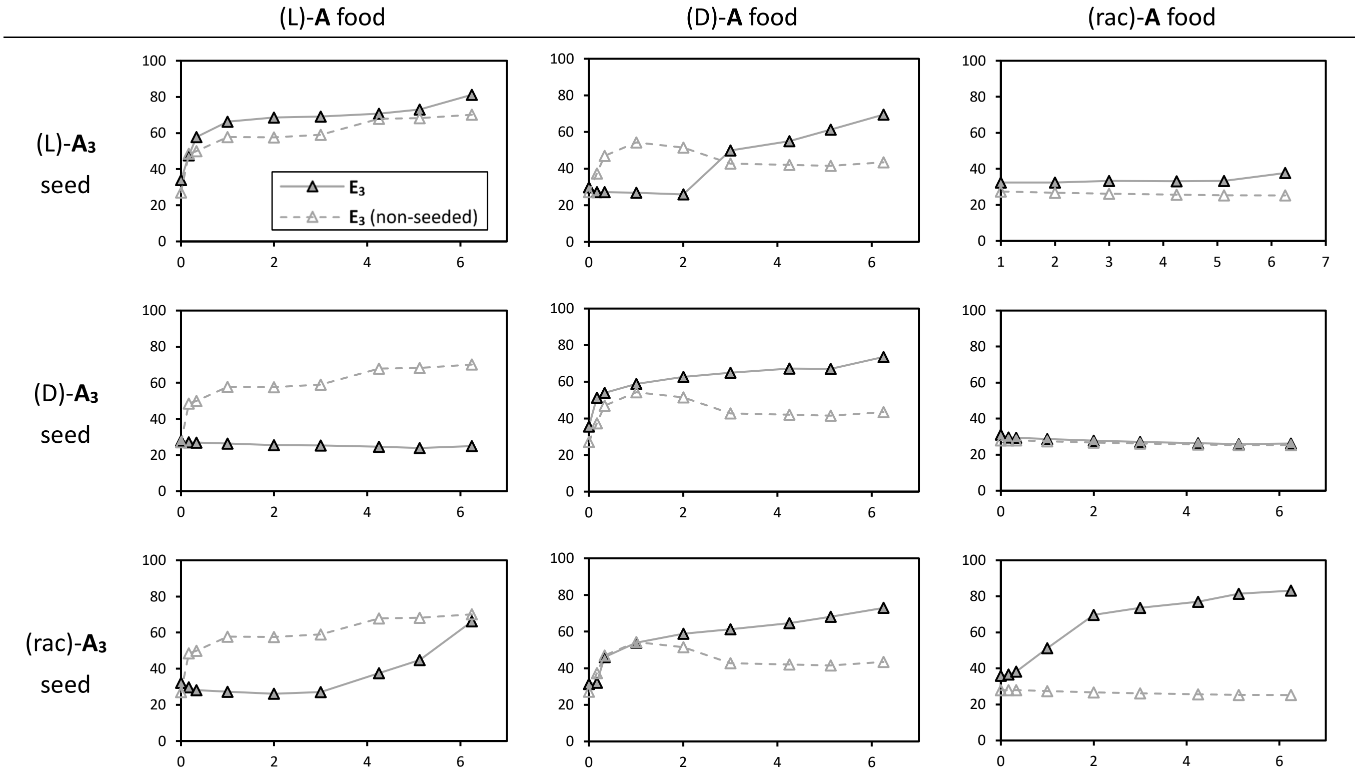

Supplementary Figure 11. *Change in product distributions of DCLs containing preoxidized **A** ("food"; consisting mostly of unassembled **A**$_1$, **A**$_3$ and **A**$_4$) to which an aliquot of a "seed" library (consisting of >90% **A**$_3$) was added at the beginning of the experiment. Food and seed chiralities of the individual experiments are as indicated. Vertical axes: % of total peak area; Horizontal axes: time [days]; Filled triangles/line: **A**$_3$ evolution in seeded experiments; Open triangles/dashed line: growth of **A**$_3$ in control experiments without seed added. Reaction conditions: 1.9 mM **A**, 80 mol% $NaBO_3$, 10 mol% **A**$_3$ seed, 4 M GuHCl, 1200 rpm shaking at 20 °C.*

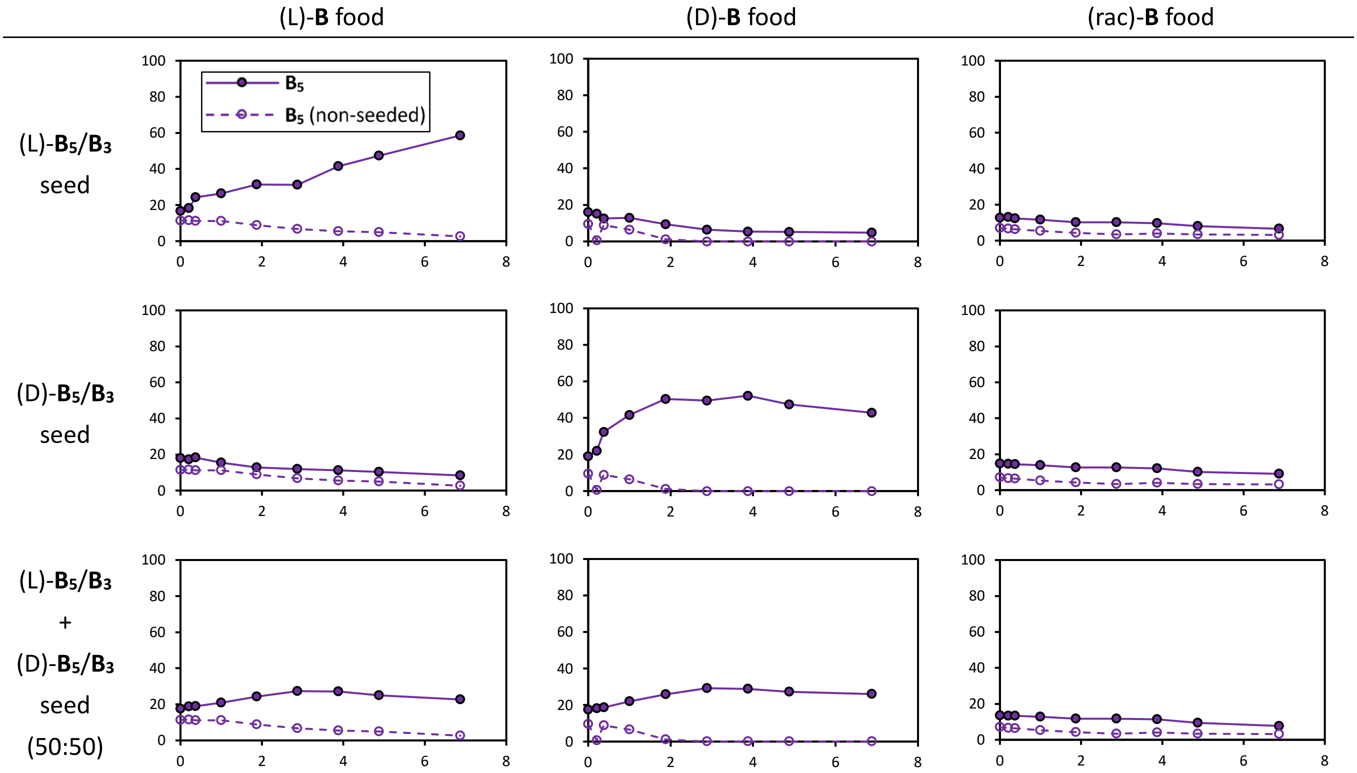

Supplementary Figure 12. *Change in product distributions of DCLs containing preoxidized **B** ("food"; consisting mostly of unassembled **B**$_1$**,** **B**$_3$ and **B**$_4$) to which an aliquot of a "seed" library (consisting of >80% **B**$_5$/**B**$_3$) was added at the beginning of the reaction. Food and seed chiralities of the individual experiments are as indicated. Vertical axes: % of total peak area; Horizontal axes: time [days]; Filled circles/solid line: **B**$_5$ evolution in seeded experiments; Open circles/dashed line: **B**$_5$ evolution of control experiments without seed added. Reaction conditions: 1.0 mM **B**, 50 mol% $NaBO_3$, 15 mol% seed (resulting in ca. 10 mol% **B**$_5$ and ca. 5 mol% **B**$_3$), 1200 rpm stirring at 45 °C.*

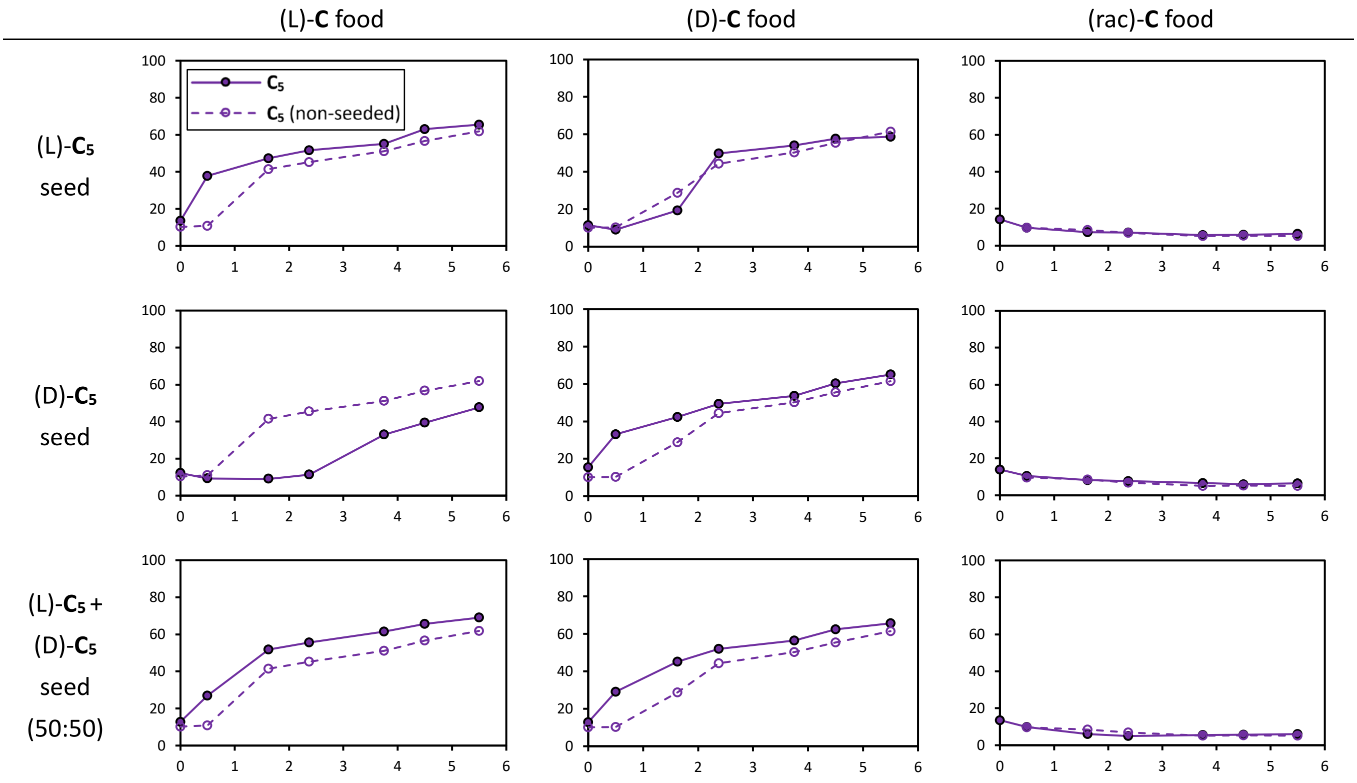

Supplementary Figure 13. *Change in product distributions of DCLs containing preoxidized **C** ("food"; consisting mostly of unassembled $\mathbf{C_1}$, $\mathbf{C_3}$ and $\mathbf{C_4}$) to which an aliquot of a "seed" library (consisting of >80% $\mathbf{C_5}$) was added at the beginning of the reaction. Food and seed chiralities of the individual experiments are as indicated. Vertical axes: % of total peak area; Horizontal axes: time [days]; Filled circles/solid line: $\mathbf{C_5}$ evolution in seeded experiments; Open circles/dashed line: $\mathbf{C_5}$ evolution of control experiments without seed added. Reaction conditions: 2.0 mM **C**, 50 mol% $NaBO_3$, 10 mol% $\mathbf{C_5}$ seed, 1200 rpm stirring at 45 °C.*

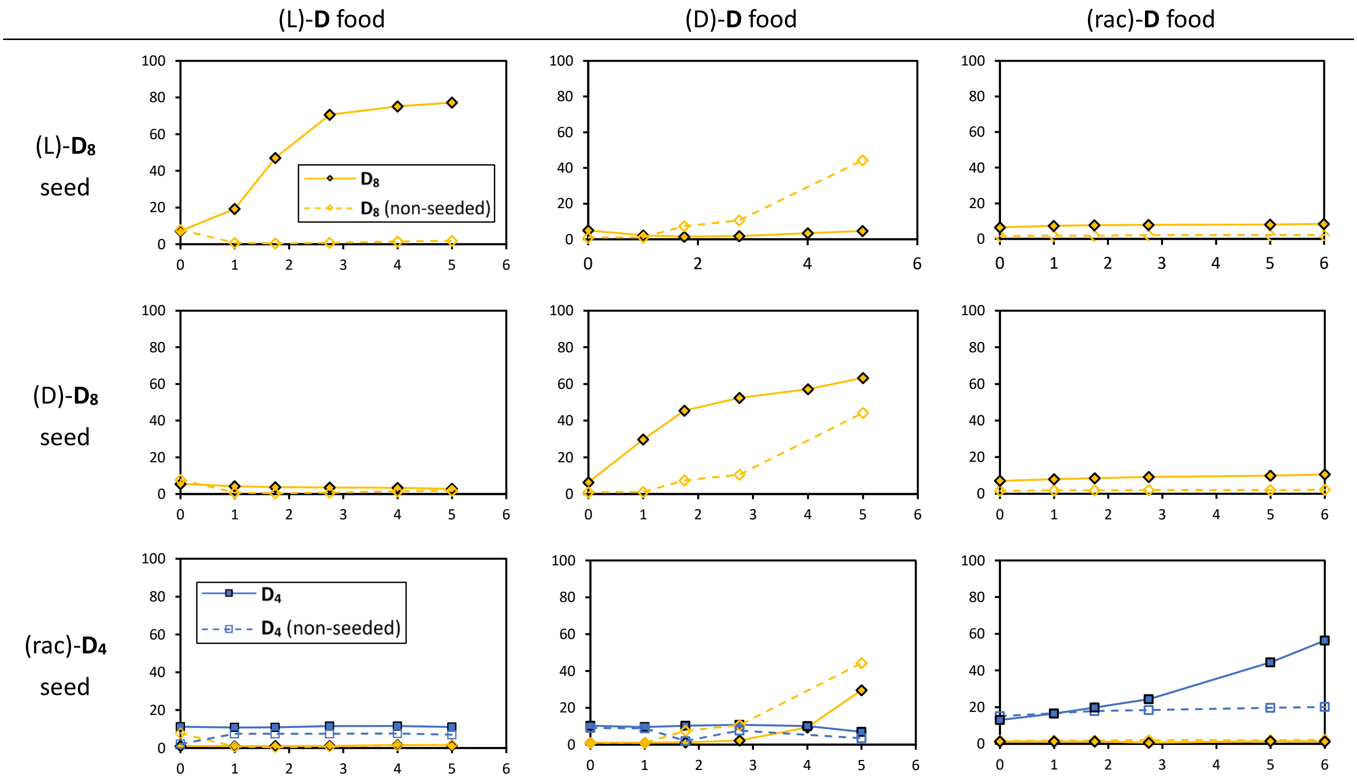

Supplementary Figure 14. *Change in product distributions of DCLs containing preoxidized **D** ("food"; consisting mostly of unassembled **D_1**, **D_3** and **D_4**) to which an aliquot of a "seed" library (consisting of >90% enantiopure **D_8** or (rac)-**D_4**) was added at the beginning of the reaction. Food and seed chiralities of the individual experiments are as indicated. Vertical axes: % of total peak area; Horizontal axes: time [days]; Solid lines: evolution of **D_8** (yellow diamonds) or **D_4** (blue square) in seeded experiments; Dashed line: growth of **D_8** (yellow open diamonds) or **D_4** (blue open square) in control experiments without seed added. Reaction conditions: 0.45 mM **D**, 50 mol% $NaBO_3$, 10 mol% **D_8** or (rac)-**D_4** seed, 1200 rpm stirring at room temperature.*

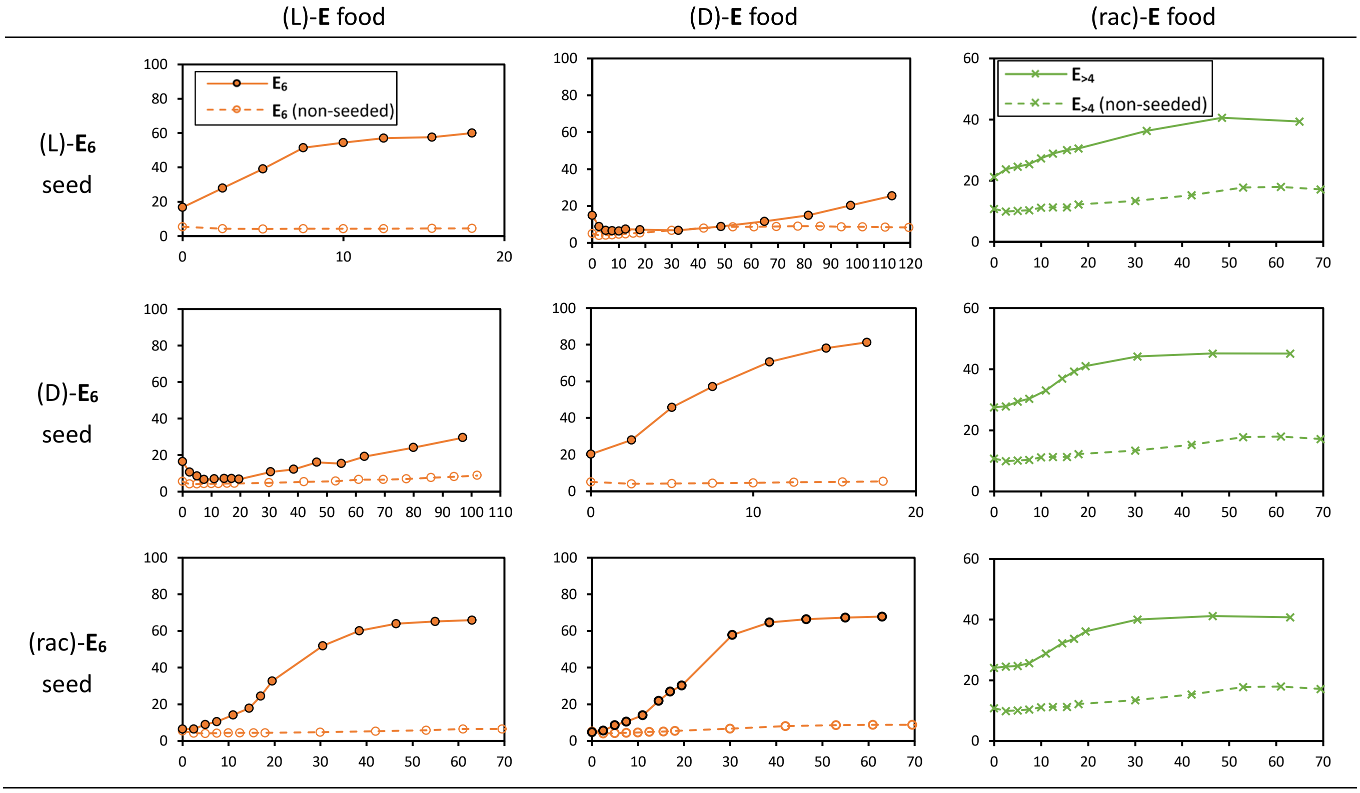

Supplementary Figure 15. *Change in product distributions of DCLs containing preoxidized* ***E*** *("food"; consisting mostly of unassembled* ***E_1, E_3*** *and* ***E_4****) to which an aliquot of a "seed" library (consisting of >90%* ***E_6****) was added at the beginning of the reaction. Food and seed chiralities of the individual experiments are as indicated. Vertical axes: % of total peak area; Horizontal axes: time [hours]; Filled circles /solid line: homochiral* ***E_6*** *evolution in seeded experiments; Open circles/dashed line: growth of homochiral* ***E_6*** *in control experiments without seed added. In experiments with racemic food the total abundance of all large macrocycles (with a ring size larger than 4) is shown instead as green crosses (solid line: seeded experiments, dashed line: non-seeded control experiments) since the heterochiral* ***E_6*** *species overlap with most large macrocycles in the UPLC chromatograms. Reaction conditions: 0.5 mM* ***E****, 80 mol% $NaBO_3$, 20 mol%* ***E_6*** *seed, 500 rpm stirring at 40 °C in an automated stirring device[2] with direct injection from the reaction medium into the UPLC (injection volume: 4 µL). The seed aliquots contained 0.5 mM* ***E_6*** *and 100 mM GuHCl, leading to an overall GuHCl concentration of 17 mM in the DCLs; in the control experiments an aliquot of a 100 mM GuHCl solution (in borate buffer; 50 mM in B atoms), without any* ***E_6****) of the same volume was added instead, leading to the same overall GuHCl concentration of 17 mM.*

Note: for the seed/food chiralities L/D, D/L, rac/L and rac/D only the evolution of homochiral ***E_6*** *is plotted although we observe also some heterochiral* ***E_6*** *species (probably due to the seeds leaking some* ***E*** *of opposite chirality into the otherwise enantiopure food). These are recognizable in UPLC at a late stage of the reaction, through their distinctive broad peak shape, but cannot be quantified properly due to peak overlap with other large macrocycles.*

2 Materials and methods

2.1 General materials & methods

Peptide building blocks were purchased from Cambridge Peptides Ltd (Birmingham, UK) ((L)-/(D)-**A,** (L)-**B**) and from Genscript ((D)-**B**, (L)-/(D)-**C**, (L)-/(D)-**D**). (L)-/(D)-**E** was made following a previously reported solution-phase procedure.[1] Doubly distilled water was used in all experiments. The buffer ingredients boric anhydride and sodium hydroxide were obtained from Merck Chemicals. Guanidinium chloride was purchased from Roth and sodium perborate (oxidant) from Sigma Aldrich. UPLC analysis: water (ULC/MS grade), acetonitrile (ULC/MS grade) and trifluoroacetic acid (TFA) were obtained from Biosolve BV. Replicator experiments were conducted in UPLC vials (12 × 32 mm) with a Teflon-lined snap cap. Samples were either placed in an Eppendorf Thermomixer C and shaken at 1200 rpm or stirred using Teflon-coated magnetic stirring bars (2 × 2 × 5 mm) obtained from VWR.

2.2 Library preparation

Building blocks were dissolved to the desired concentration in borate buffer (50 mM in boron atoms, pH 8.2); guanidinium chloride was added if required. Each library had a volume of 0.50 mL and was shaken or stirred at 1200 rpm in the presence of oxygen from the air at the indicated temperature.

2.3 Seeding experiments

Stock solutions of the desired building blocks and chiral configurations were oxidized by adding a freshly prepared sodium perborate solution (100 mM in 50 mM boron atom buffer) followed by shaking for 10 min to give a library containing mostly monomer and unassembled trimer and tetramer. The stock solutions were then split into 0.30 mL samples; racemic libraries were obtained by mixing 0.15 mL of each (L)- and (D)-solutions. Then, aliquots of libraries containing the desired replicators were added as seeds. One sample of each (L)-, (D)- and (rac)-libraries were left unseeded as control experiments. The samples were shaken or stirred at 1200 rpm at and monitored by UPLC and UPLC-MS over time.

2.4 UPLC analysis

UPLC analyses were performed using a Waters Acquity UPLC H-class system with a reversed-phase UPLC column (Phenomenex Aeris Peptide, 2.1 × 150 mm; 1.7 μm) and a PDA detector. The column temperature was set to 35 °C, the eluent flow rate to 0.3 mL/min and UV absorbance was monitored at 254 nm. Analytic samples were prepared by diluting aliquots of the studied libraries to ca. 200 µmol/L in UPLC-grade water; the injection volume was set to 10 μL. Chromatograms were recorded and treated with Waters Empower 3 software, changes in DCL composition over time were plotted with Microsoft Excel.

Eluent A: UPLC grade with 0.1% trifluoroacetic acid added

Eluent B: UPLC grade acetonitrile with 0.1% trifluoroacetic acid added

The eluent gradients used for UPLC analysis are shown in the tables below.

*Table 1. UPLC method for the analysis of DCLs made from **A**.*

Time (min)	% water + 0.1% TFA	% MeCN + 0.1% TFA
0	90	10
1	90	10
1.3	75	25
3	72	28
11	60	40
11.5	5	95
12	5	95
12.5	90	10
17	90	10

*Table 2. UPLC method for the analysis of DCLs made from **B**.*

Time (min)	% water + 0.1% TFA	% MeCN + 0.1% TFA
0	90	10
1	90	10
1.3	75	25
3	73	27
11	70	30
11.5	5	95
12	5	95
12.5	90	10
17	90	10

*Table 3. UPLC method for the analysis of DCLs made from **C**.*

Time (min)	% water + 0.1% TFA	% MeCN + 0.1% TFA
0	90	10
1	90	10
1.3	75	25
3	72	28
9	60	40
11	45	55
11.5	5	95
12	5	95
12.5	90	10
17	90	10

*Table 4. UPLC method for the analysis of DCLs made from **D**.*

Time (min)	% water + 0.1% TFA	% MeCN + 0.1% TFA
0	90	10
1	85	15
5	65	35
5.5	30	70
13	5	95
13.5	5	95
14	90	10
17	90	10

*Table 5. UPLC method for the analysis of DCLs made from **E**.*

Time (min)	% water + 0.1% TFA	% MeCN + 0.1% TFA
0	60	40
1	60	40
3	35	65
17	10	90
19	5	95
21	5	95
22	60	40
24	60	40

We reported earlier for systems made of building blocks **A**[3] and **B**[4] that the different macrocycles of a system have a similar molar absorptivity per building block unit at 254 nm, which allows to directly compare their UPLC peak areas; thus, the percentages shown in the graphs tell how much of the total building blocks resides within a specific macrocycle size. This is true for macrocycles of a same building block as well as in mixed systems, since the absorptivity depends mostly on the 3,5-mercaptobenzoyl core which is identical in all building blocks. In addition, we show here, as representative examples, the complete kinetic traces of DCLs made of **C**, **D** and **E** as well as their associated total peak area over time (Supplementary Figure 16). The total peak area stays constant over time in all three cases, although the proportions of different macrocycles change significantly over the course of the experiments, indicating that the molar absorptivity is approximately the same for all species.

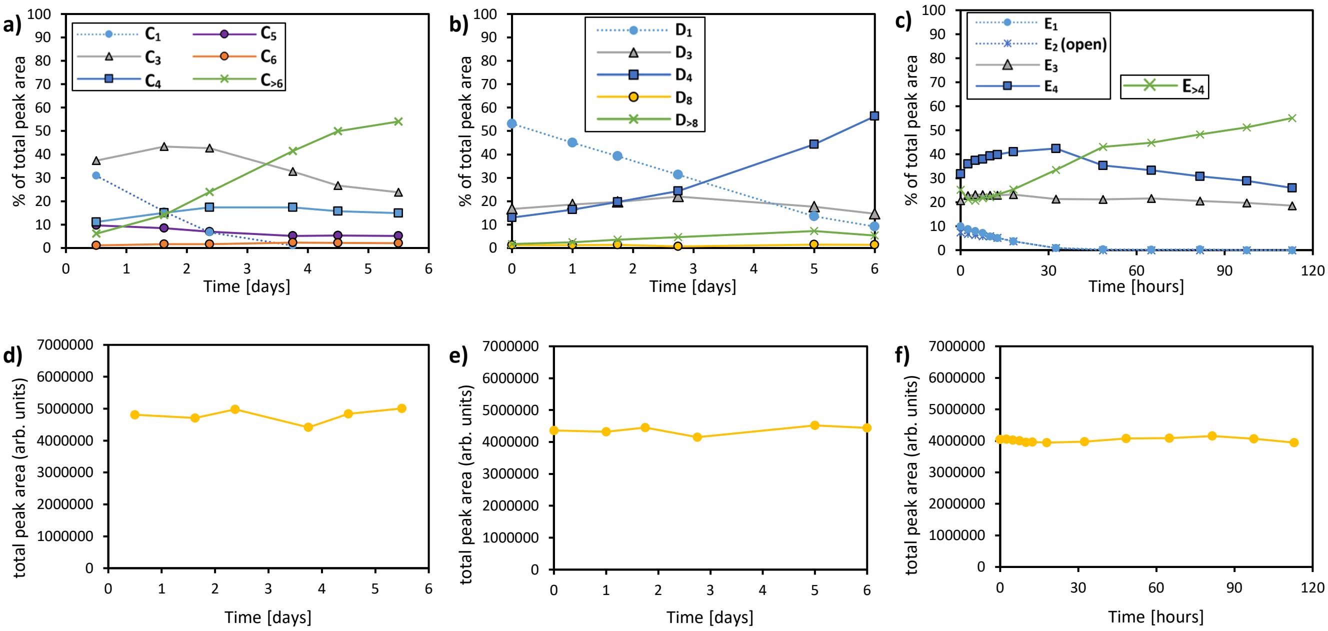

Supplementary Figure 16. *Changes in product distributions with time in DCLs obtained from building blocks (a)* ***C****, (b)* ***D*** *and (c)* ***E****; changes of the absolute total peak area with time associated to the respective libraries (d:* ***C****, e:* ***D*** *and f:* ***E****). Reaction conditions: 2 mM (rac)-****C****, 45°C, 50 mol% $NaBO_3$, 1200 rpm stirring (a & d), 0.45 mM (rac)-****D****, rt, 10 mol% (rac)-$\mathbf{D_4}$ seed, 50 mol% $NaBO_3$, 1200 rpm stirring (b & e), 0.50 mM* ***E****, 40 °C, 20 mol% (D)-$\mathbf{E_6}$ seed, 80% $NaBO_3$, 500 rpm stirring in automated stirring device (c & f); all libraries were prepared in borate buffer (50 mM in B atoms).*

2.5 UPLC-MS analysis & data treatment

UPLC-MS analyses were performed on a Waters Xevo G2 UPLC/TOF. Electro-spray ionization (ESI) was used to acquire positive-ion mass spectra. The capillary, sampling cone and extraction cone voltages were set at 2.5 kV, 30 kV and 4 V, respectively. Nitrogen was used as cone and desolvation gas with flow rates of 5 L/h and 500 L/h, respectively. The temperatures of source and desolvation were 150°C and 500°C, respectively. Eluent gradients and sample preparation were identical to UPLC analyses, with the sample concentration being ca. 400 µmol/L and the injection volume 5 µL. Mass spectra were usually recorded as peak centroids, except in (L*)/(D)-experiments where quantification of diastereoisomers was required. There, continuum mode was used to allow for peak integration. UPLC-MS spectra were recorded with MassLynx software.

For the estimation of $\mathbf{A_5}$ diastereoisomeric ratios in (L*)/(D)-experiments (main text Figure 4 and Supplementary Figure 6) the mass spectrum of the (D_5)-$\mathbf{A_5}$ isomer was simulated using mMass software (Supplementary Figure 17a) and the resulting 13 peaks were integrated using Origin Pro 2018 64bit (Supplementary Figure 17b). The area values of the 13-peakset were then multiplied with a factor to fit the integrated MS data from the corresponding experiment (Supplementary Figure 17c). The right factor was found by calculating the absolute differences (as percentages of the corresponding experimental value) of the first seven simulated and experimental areas, averaging these differences and then minimizing the average value by letting Excel's solver module (not included in Microsoft Excel standard package, needs to be installed separately) optimize the factor. The procedure was repeated successively for the (L*D_4), (L*$_2D_3$), (L*$_3D_2$), (L*$_4$D) and (L*$_5$) isomers of $\mathbf{A_5}$ (Supplementary Figure 17d), taking into account that the first six peaks of each isomer overlap with the last six of the previous one; factor optimization thus fits the sum of overlapping peak

areas to the target experimental peak area, except for D_5 which is not preceded by any isomer. The diastereomeric ratios within (L*)- or (D)-rich $\mathbf{A_5}$ species were then determined from the found factors for each diastereomer (Supplementary Figure 17e).

The minimal mean absolute difference is mostly within 6% for the major peaks (D_5 and L^*_5) but can go up to 22% for the low intensity peaks ($L^*_2D_3$ and $L^*_3D_2$) since these are proportionally more affected by noise and artifacts in MS. To assess the percentage of a particular e. g. (D)-rich diastereomer within a (D)-rich replicator, its factor is divided by the sum of the factors of all three (D)-rich diastereomers. The error within the (D)-rich replicator is then calculated by summing up the errors in each (D)-rich diastereomer (0, 1/5 and 2/5), weighed each with its respective proportion (64, 30 and 6% in Supplementary Figure 17e, giving an error of 8.4% in (D)-rich replicator).

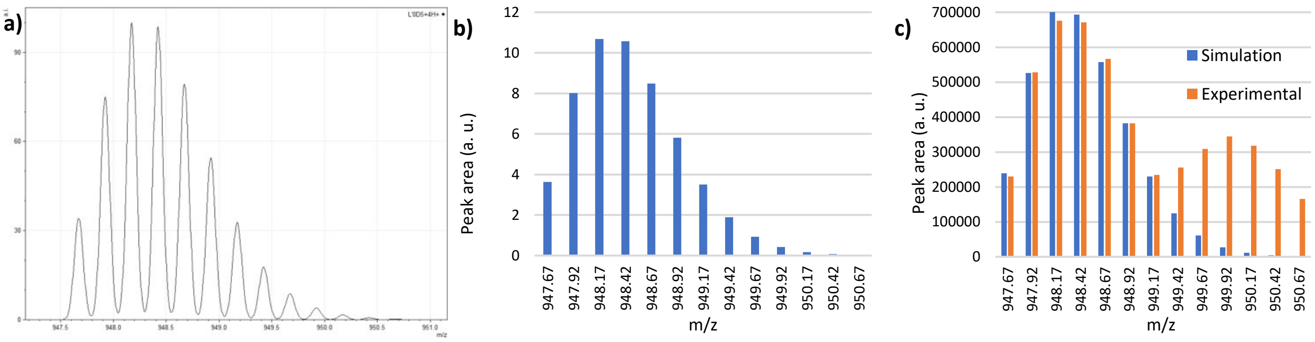

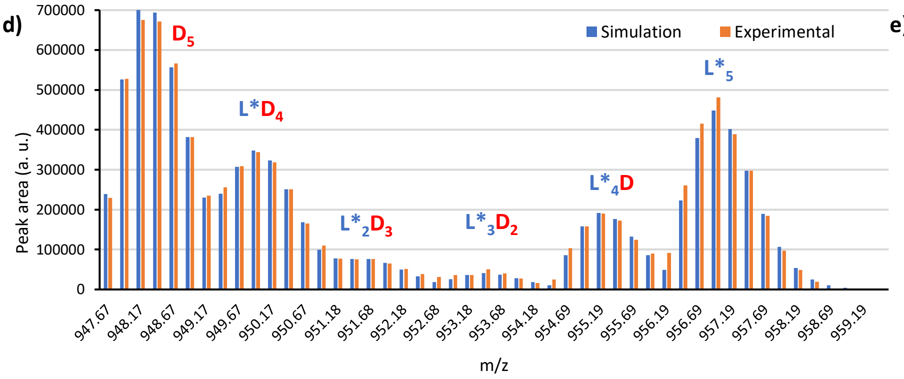

e)

Diastereo-isomer	Mean abs. diff. [%]	Factor	% of (D)- or (L*)-rich isom.
D_5	2	65665	64
L^*D_4	3	30091	30
$L^*_2D_3$	9	6086	6
$L^*_3D_2$	19	3657	6
L^*_4D	11	17896	28
L^*_5	6	41537	66
Error in (D)-repl.: 8.4%		Error in (L*)-repl.: 8.0%	

Supplementary Figure 17. *Representative examples for a) a simulation of a 13 peak-set (here (D_5)-$\mathbf{A_5}$, M + 4 H^+), b) integral values of the 13 peak-set, c) simulated peak area set (blue) fitted to experimental data (orange), d) full D_5 to L^*_5-simulated peak area sets (blue) fitted to experimental peak areas (orange), e) minimized mean absolute difference between simulated and experimental peak areas (as percentage of the experimental value), the factors found from these minimized differences and the resulting percentage of each diastereoisomer within (L*)- or (D)-rich* $\mathbf{A_5}$*. Experimental data corresponds to the (L*)/(D)-seeding experiment, main text Figure 4f, day 28.*

2.6 MALDI-TOF analysis

($L^*_2D_2$)-$\mathbf{A_4}$ was isolated from an (L)-**A***/(D)-**A** library (containing $\mathbf{A_4}$ in ca. 15% of total **A** units, cf. Figure 4 and Supplementary Figure 7) by separating the mixture on UPLC with the eluent method for **A** described above, from a more concentrated sample than usual (1.52 mM total **A*/A**), and collecting the UPLC outflow right after ($L^*_2D_2$)-$\mathbf{A_4}$ passed the PDA detector. UPLC analysis of the collected material (direct injection, no further dilution) confirmed that

it contained only $\mathbf{A_4}$.

A solution of α-cyano-4-hydroxycinnamic acid (CHCA) was prepared by dissolving 10 mg of CHCA in 1.0 mL of an acidified water/acetonitrile mixture (50:50 + 0.10 vol% TFA). The isolated (L*$_2$D$_2$)-$\mathbf{A_4}$-solution was mixed with the CHCA solution in ratios of 1:5, 1:10 and 1:20. Each sample:CHCA ratio was spotted three times on a MALDI plate (384 Opti-TOF 123 x 81 mm; 1.0 µL per spot). The spots were left to dry for one hour before analysis. Spectra were recorded using an Applied Biosystems 4800 MALDI TOF/TOF spectrometer operated in linear positive mass mode. Various mass ranges were analyzed with 20 subspectra (containing 50 shots) each. The laser was moved to a different position of the spotted sample before every sub-spectrum. Obtained mass spectra were visualized using mMass (open source mass spectrometry software) and peak integration was performed with Origin 2018 64bit.

2.7 Transmission Electron Microscopy (TEM)

A droplet (5 µL) of sample was deposited on a copper grid (400 mesh) covered with a carbon film (Van Loenen Instruments). The droplet was blotted on filter paper after 30 s and the sample was stained twice (5 µL each time) with a 2% uranyl acetate solution (deposited on the grid and blotted on filter paper after 30 s each time). The grids were observed in a cryo-electron microscope at 120 keV (Philips CM120). Images were recorded by CCD camera and analyzed with ImageJ software.

2.8 Circular Dichroism (CD)

Spectra were obtained at 20 °C using a JASCO J715 spectrophotometer (range = 210–450 nm, pitch = 0.1 nm, bandwidth = 2 nm, D.I.T = 0.5 s, speed = 200 nm/min, continuous scanning, standard sensitivity, averaging over 3 consecutive scans). A sample containing only borate buffer was used as a blank and subtracted from all spectra. All spectra were measured using samples diluted to 0.4 mM with borate buffer (50 mM in B atoms) in HELMA quartz cuvettes (1 mm path length). $\mathbf{A_3}$ samples were diluted in a 4 M GuHCl/50 mM borate buffer solution, which gave more reproducible signals; for $\mathbf{A_5}$/$\mathbf{A_3}$ diluting with or without 4 M GuHCl did not make a difference.

2.9 Thioflavine T (ThT) fluorescence

A ThT stock solution (2.2 mM) was prepared in 10 mL borate buffer (100 mM B_2O_3, pH 8.2) and filtered through a 0.2 µm syringe filter. On the day of analysis, 50 µL of the stock solution was diluted into 5 mL phosphate buffer (50 mM phosphate, pH 8.2) to generate the working solution of 22 µM. The fluorescence intensity of 450 µL ThT solution was measured by excitation at 440 nm (slit width 5 nm) and emission between 480-700 nm (slit width 5 nm), averaging 3 accumulations, in a HELMA 10*2 mm quartz cuvette (2 mm large side facing the excitation beam). An aliquot of 80 µL of a DCL (diluted with phosphate buffer to 80 µM in building block concentration) was then added to the ThT solution, the mixture was shaken thoroughly, and the emission intensity was measured over 3 accumulations. All fluorescence measurements were performed on a JASCO FP6200 fluorimeter equipped with a 480 nm high pass cut-off filter on the emission channel to avoid high order diffractions coming from the excitation. Samples with **B** were measured using half concentration (80 µL of a DCL diluted to 40 µM).

3 Characterization data

3.1 TEM

Note: the scale bars are all 100 nm wide, except in Supplementary Figure 21b (200 nm).

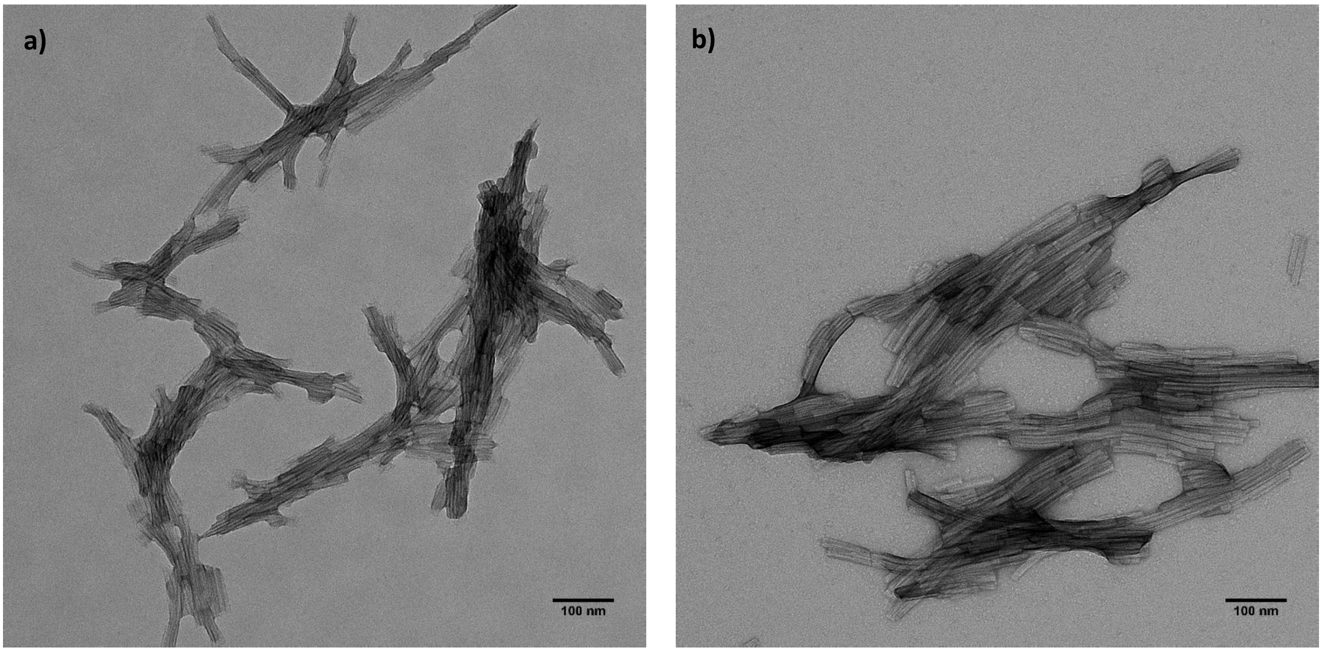

Supplementary Figure 18. *TEM micrographs of samples dominated by (L)- and (D)-**A_5/A_3** (panels a and b, respectively; both in a 60:35 ratio) in a DCL made from 3.8 mM **A** in borate buffer (50 mM in B atoms), 4 M GuHCl and shaken at 1200 rpm at 20 °C.*

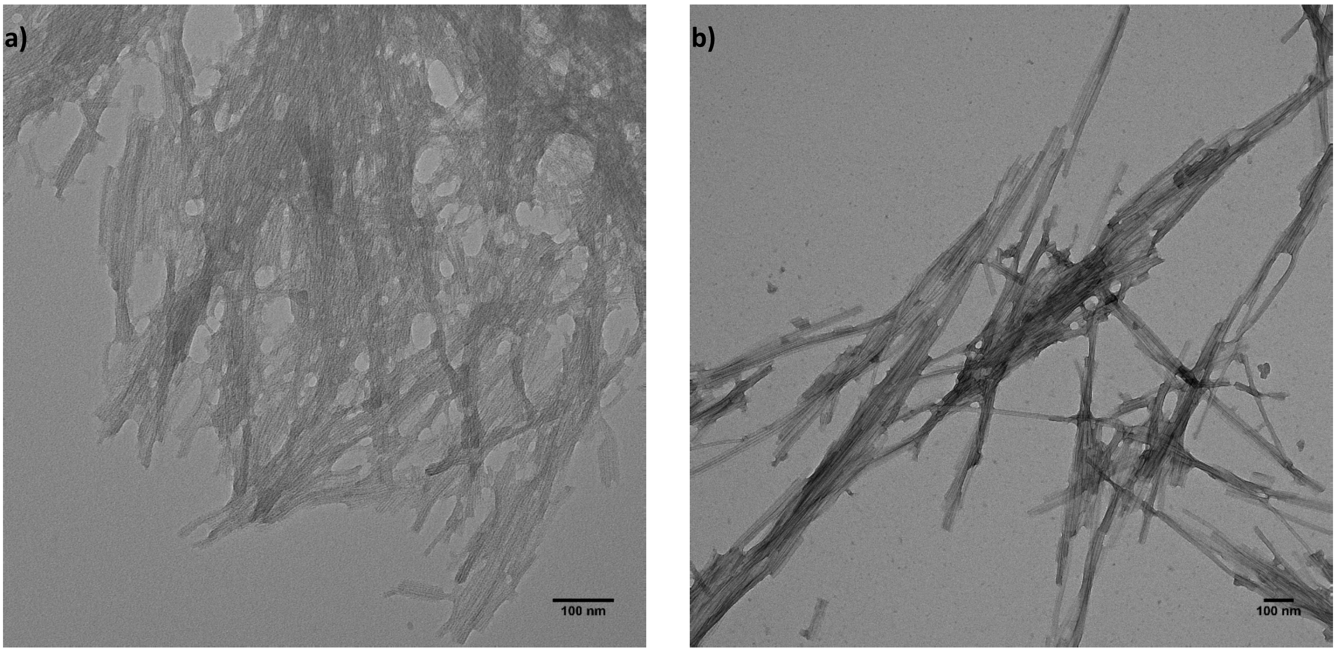

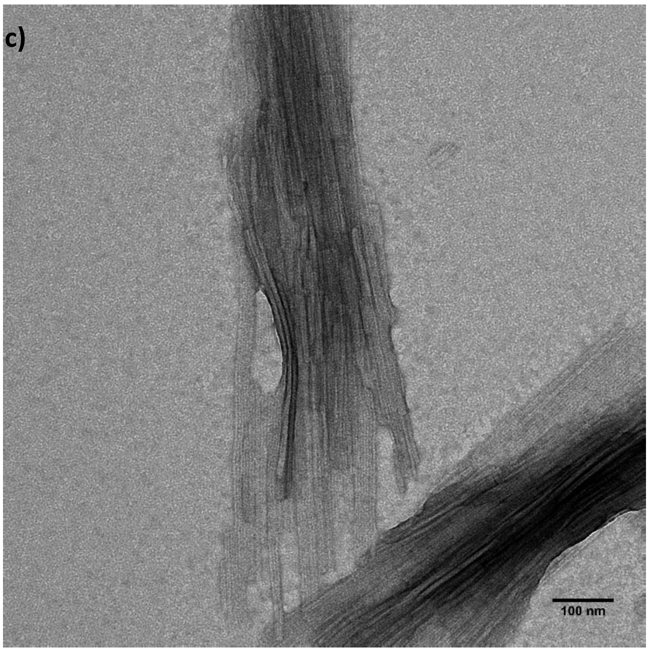

Supplementary Figure 19. *TEM micrographs of a sample dominated by (L)-, (D)- and (rac)-**A_3** (panels a, b and c, respectively) in a DCL made from 3.8 mM **A** in borate buffer (50 mM in B atoms), GuHCl (a: 4.5 M, b: 2.5 M, c: 4 M) and shaken at 1200 rpm at 20 °C.*

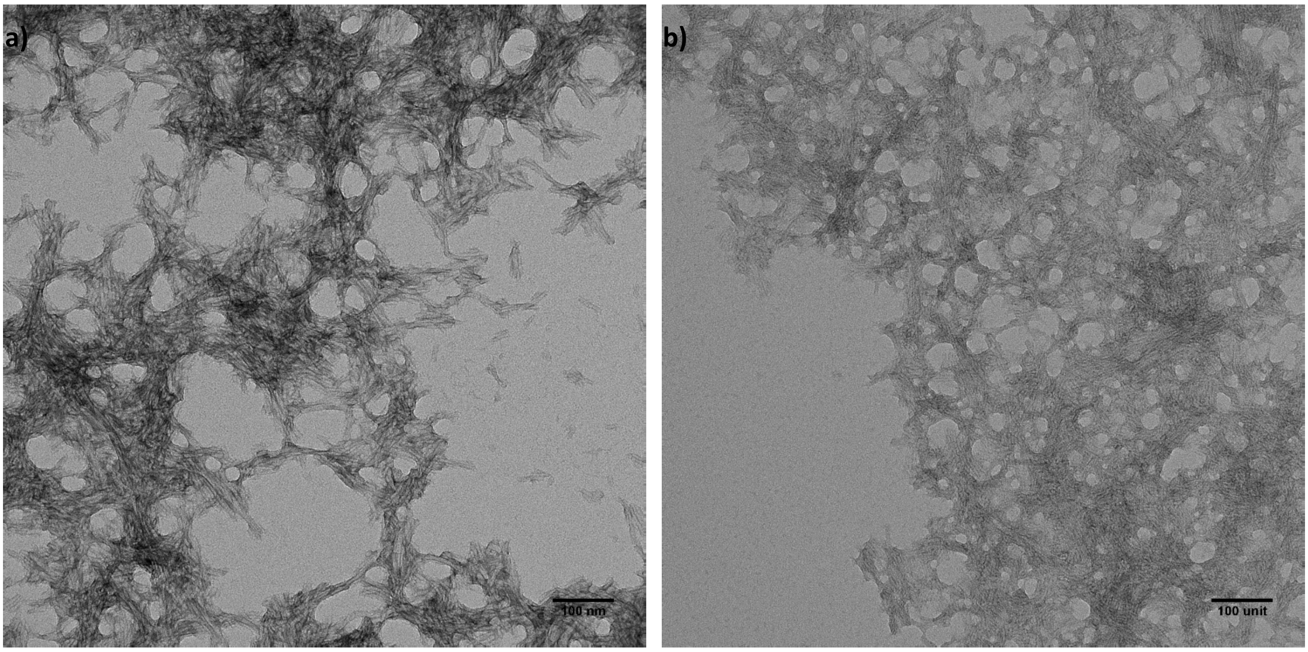

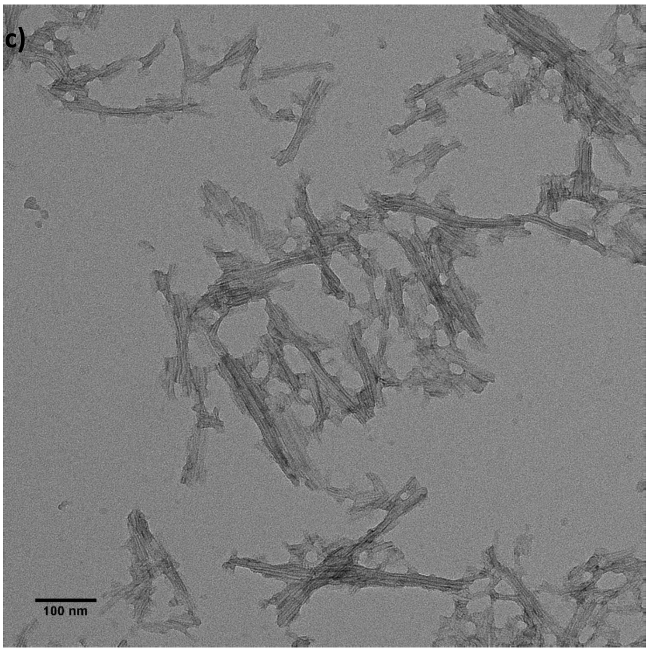

Supplementary Figure 20. *TEM micrographs of a sample dominated by (L)-, (D)- and (rac)-* $\mathbf{B_5}$/$\mathbf{B_3}$ *(panels a, b and c, respectively) in a DCL made from 2.0 mM* ***B*** *in borate buffer (50 mM in B atoms) and stirred at 1200 rpm at 45 °C.*

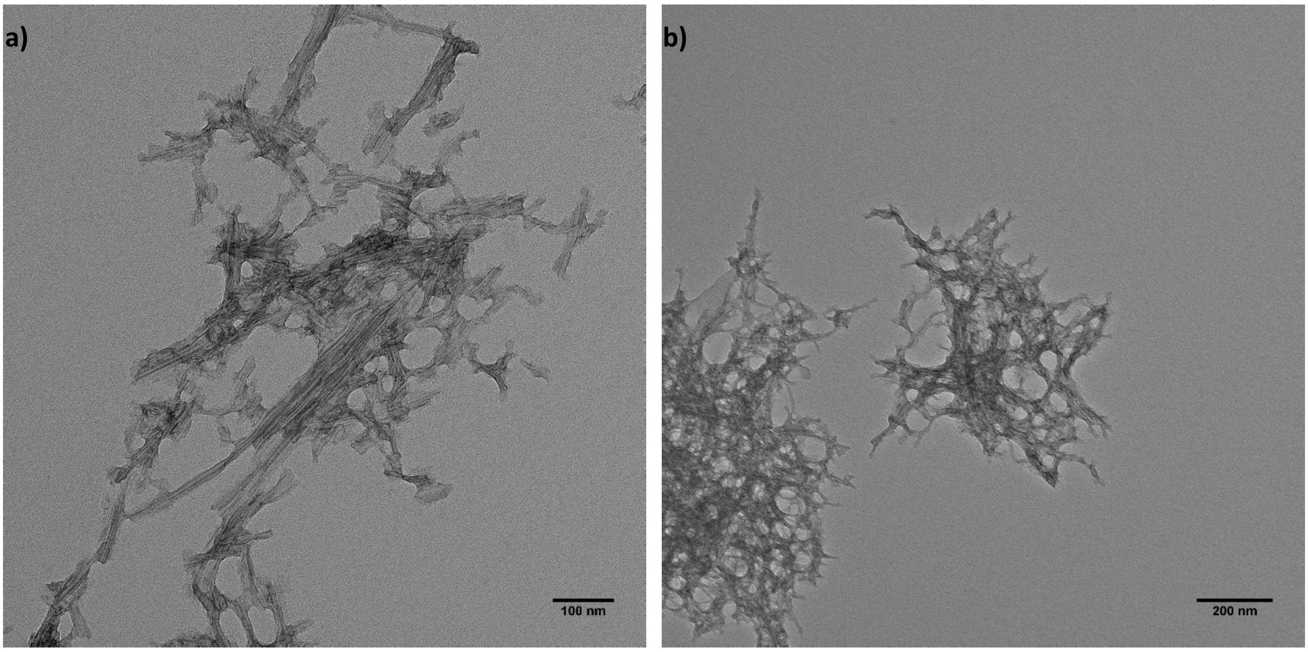

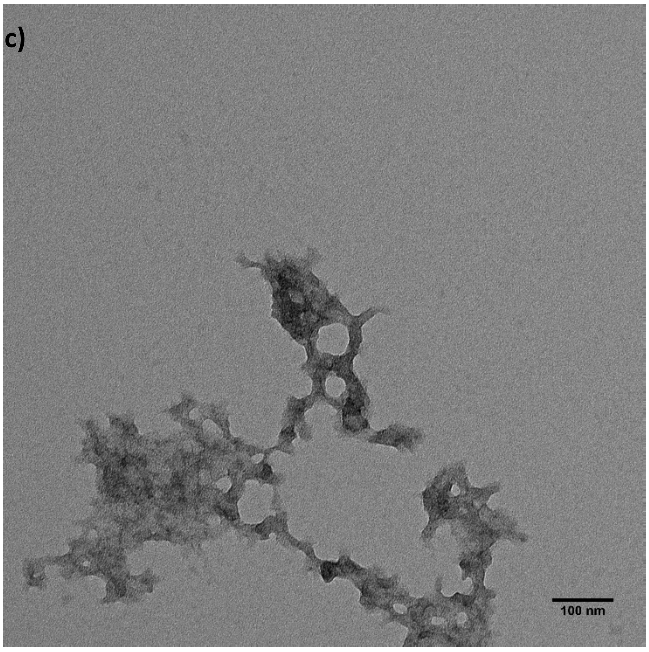

Supplementary Figure 21. *TEM micrographs of a sample dominated by (L) and (D)-**C_5** (panels a and b, respectively) and of the mixture of macrocycles obtained from (rac)-**C** (panel c) in DCLs made from 2.0 mM **C** in borate buffer (50 mM in B atoms) and stirred at 1200 rpm at 45 °C.*

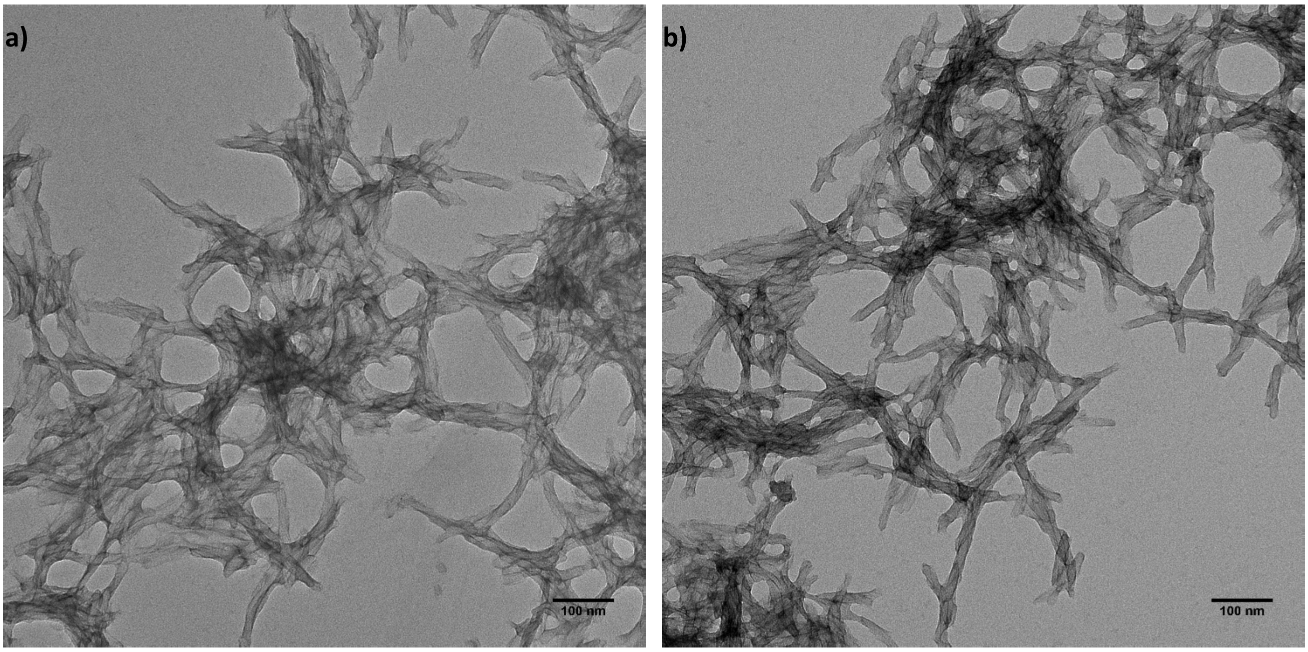

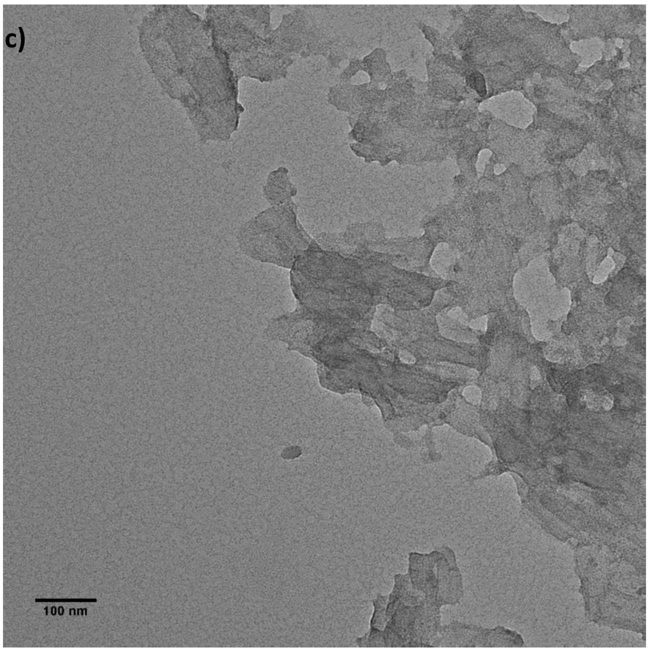

Supplementary Figure 22. *TEM micrographs of samples dominated by (L)- and (D)-**D_8** (panels a and b, respectively) and (rac)-**D_4** (panel c) in DCLs made from 0.45 mM **D** in borate buffer (50 mM in B atoms), stirred at 1200 rpm at rt.*

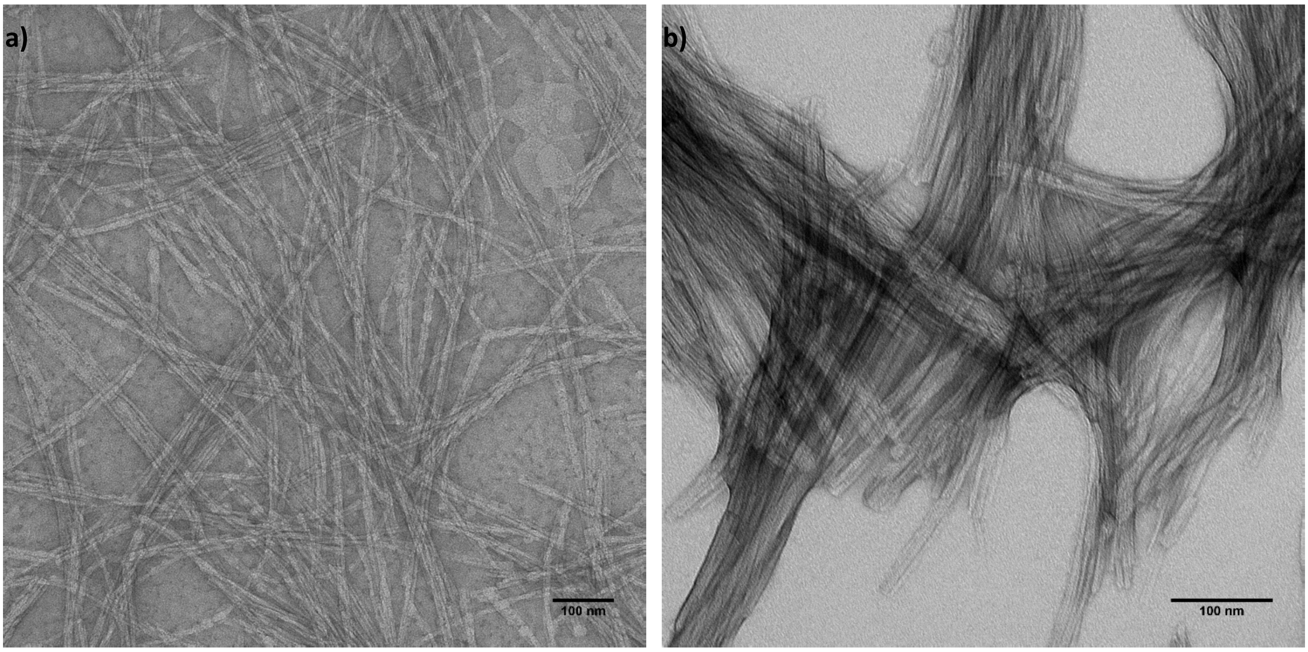

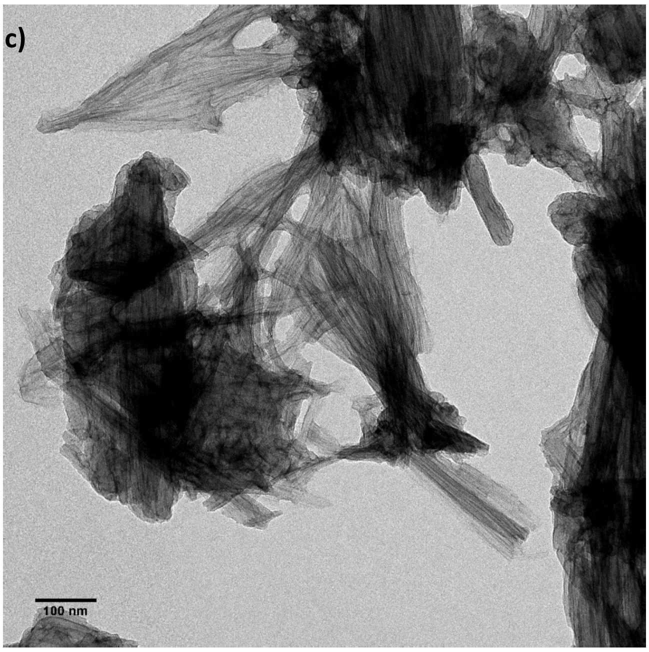

Supplementary Figure 23. *TEM micrographs of samples dominated by (L)-, (D)- and (rac)-**E_6** (panels a, b and c, respectively) obtained in a DLC made from 1.0 mM **E** in borate buffer (50 mM in B atoms), GuHCl (a: 10 mM, b and c: 1 M), stirred at 1200 rpm at 45 °C (a) or rt (b and c).*

3.2 Circular Dichroism

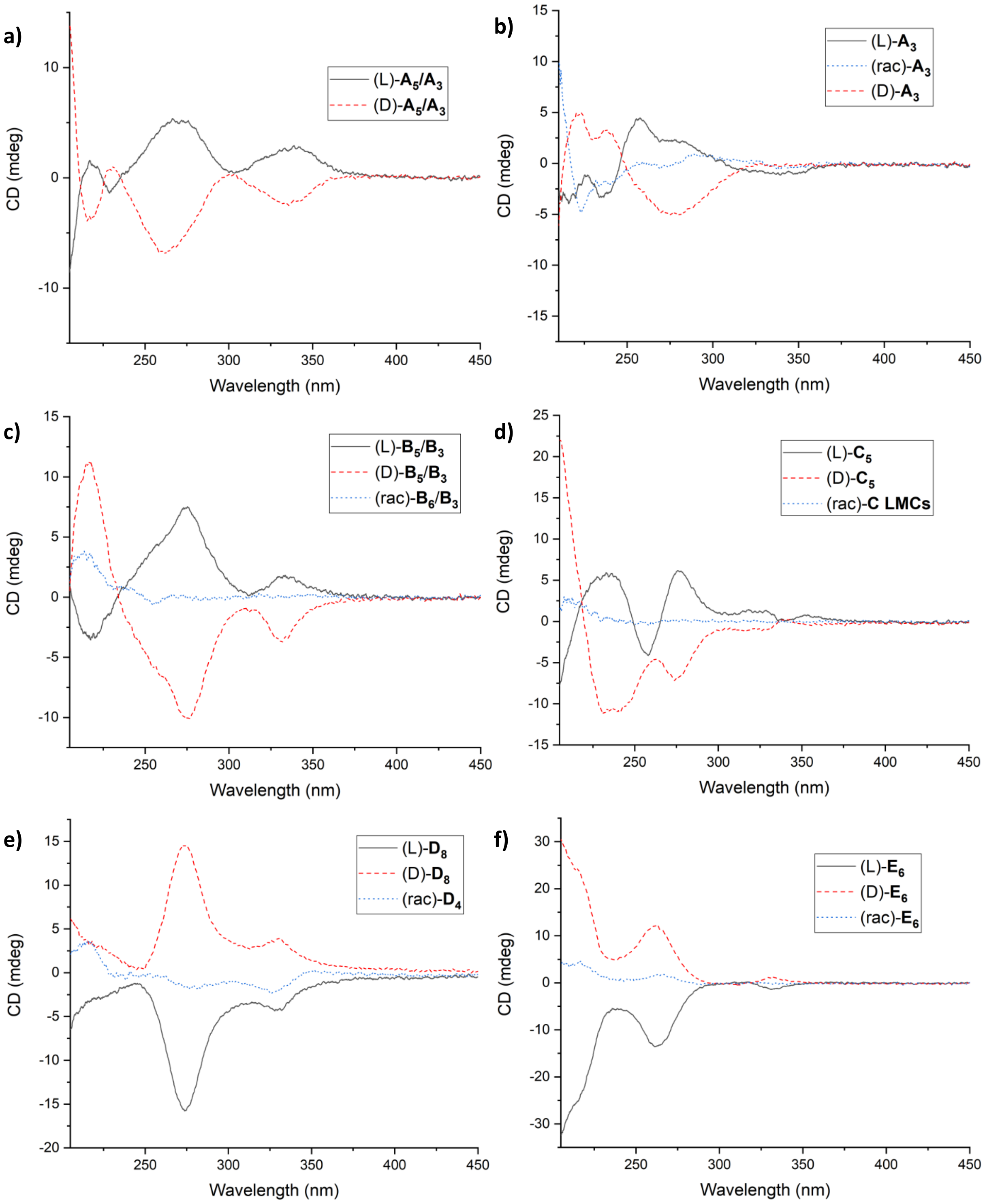

***Supplementary Figure 24**. CD spectra for a) (L)- and (D)-**A_5/A_3**, b) (L)-, (D)- and (rac)-**A_3**, c) (L)- and (D)-**B_5/B_3** and (rac)-**B_6/B_3**, d) (L)- and (D)-**C_5** and (rac)-**C** large macrocycles (LMC), d) (L)- and (D)-**D_8** and (rac)-**D_4**, e) (L)-, (D)- and (rac)-**E_6**. All spectra were measured at a 0.4 mM concentration of the corresponding building block. For b), the baseline was measured from a 4 M GuHCl solution in 50 mM B atom buffer and the DCLs were diluted (from 3.8 mM to 0.4 mM in building block) using that same 4 M GuHCl solution.*

At 250-350 nm, (L)-libraries give positive CD and (D)-libraries negative CD signals, except for the monopeptide building blocks **D** and **E** where it is the other way round; they also show higher intensities (**E** also below 250 nm).

3.3 Thioflavin T assays

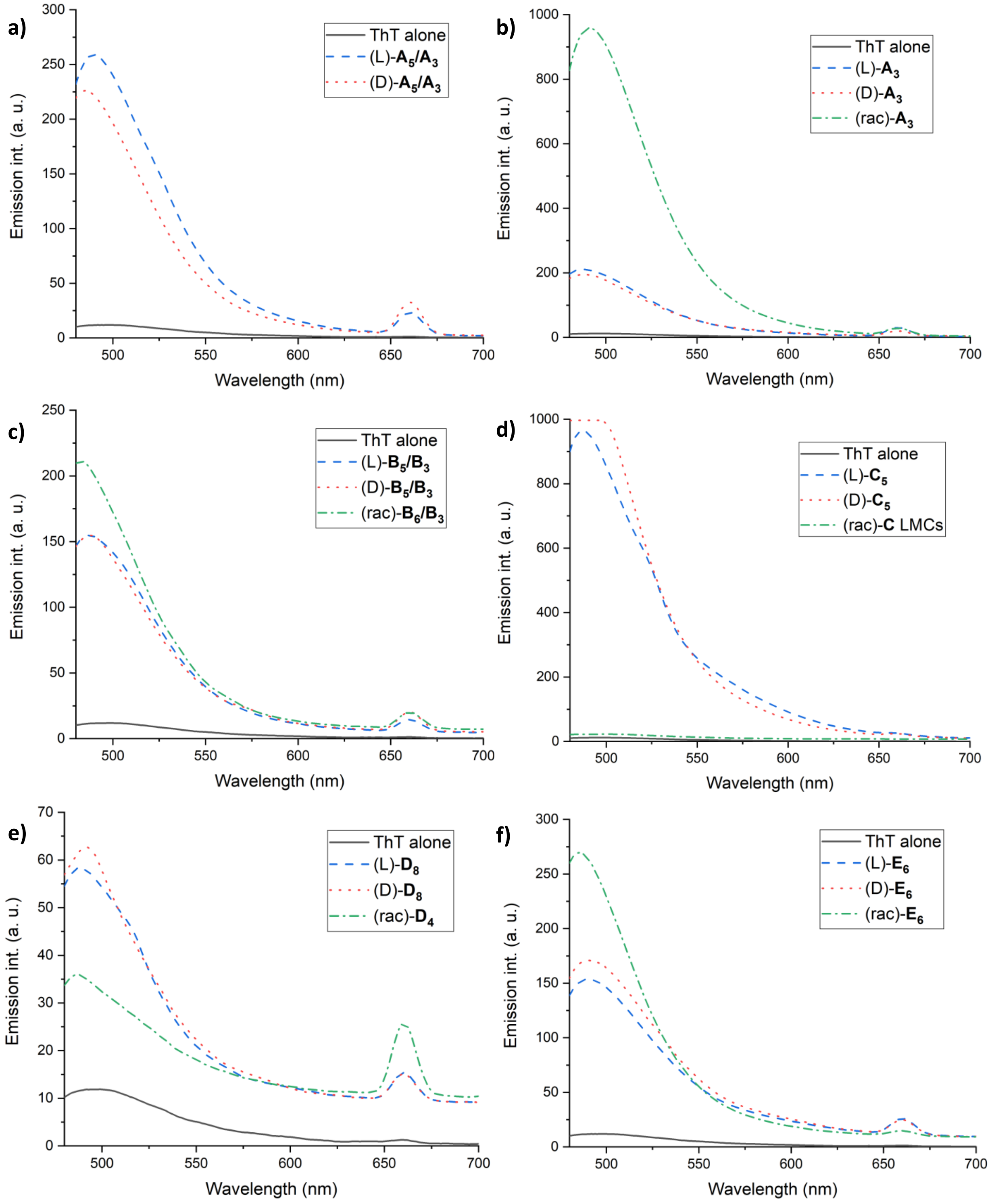

***Supplementary Figure 25**. ThT fluorescence assay spectra for a) (L)- and (D)-$\mathbf{A_5/A_3}$, b) (L)-, (D)- and (rac)-$\mathbf{A_3}$, c) (L)- and (D)-$\mathbf{B_5/B_3}$ and (rac)-$\mathbf{B_6/B_3}$, d) (L)- and (D)-$\mathbf{C_5}$ and (rac)-**C** large macrocycles (LMCs), d) (L)- and (D)-$\mathbf{D_8}$ and (rac)-$\mathbf{D_4}$, e) (L)-, (D)- and (rac)-$\mathbf{E_6}$. All spectra were measured at a 12 µM concentration of the corresponding building block except for **C** in panel d) (6 µM) in 50 mM phosphate buffer.*

All libraries (except (rac)-**C** LMCs) lead to enhanced ThT fluorescence, suggesting the presence of assembled species with β-sheets. Enantiopure libraries mostly give an emission maximum of ca. 150-250 a. u., however their racemic counterparts give a somewhat stronger ($\mathbf{B_6}$/$\mathbf{B_3}$, $\mathbf{E_6}$) or even much stronger signal ($\mathbf{A_3}$). This could be due to mixed chiral replicators having a less ordered structure, their β-sheets thus having more free space to accommodate the ThT dye. An exception is (rac)-$\mathbf{D_4}$ which gives a weaker signal than $\mathbf{D_8}$, however the emission intensity is generally lower (35-65 a. u.) – probably because the building block has only little hydrophobic surface with which the dye could interact. $\mathbf{C_5}$ gives an a much higher fluorescence response than all other systems, partly saturating the detector even when measured at half concentration; on the other hand, the mixture of (rac)-**C** LMCs, which was not found to have any replicating properties, which shows only minimal enhancement of ThT fluorescence (maximum emission of 22 a. u. vs 12 a. u. for the control sample) and thus suggests the absence of assembled species.

3.4 UPLC and UPLC-MS Analyses

3.4.1 Building block **A**

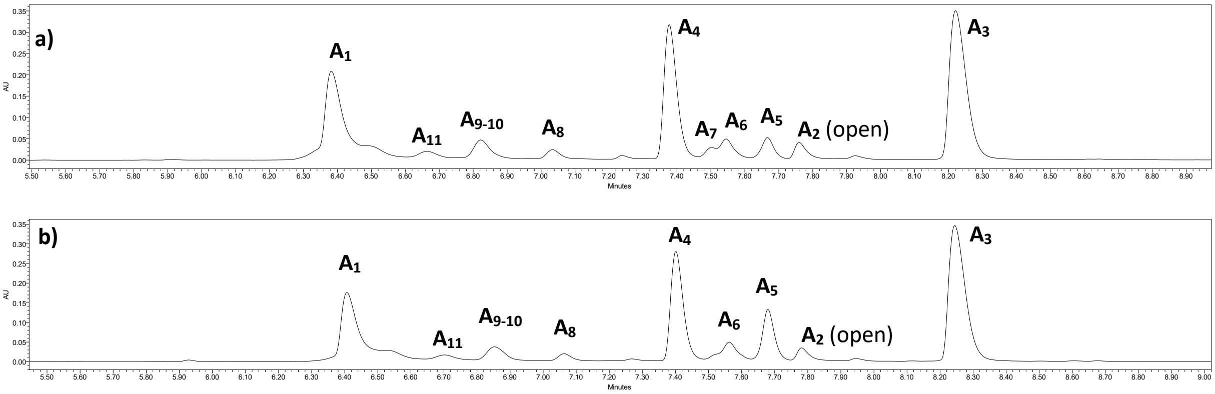

Supplementary Figure 26. *Typical UPLC traces of libraries made from a) enantiopure* ***A*** *and b) racemic* ***A*** *(3.8 mM in borate buffer with 4M GuHCl, pH 8.1) under constant mechanical agitation. Because the same species in these two traces show mostly identical elution time and have the same mass, only one mass spectrum for each species is provided below.*

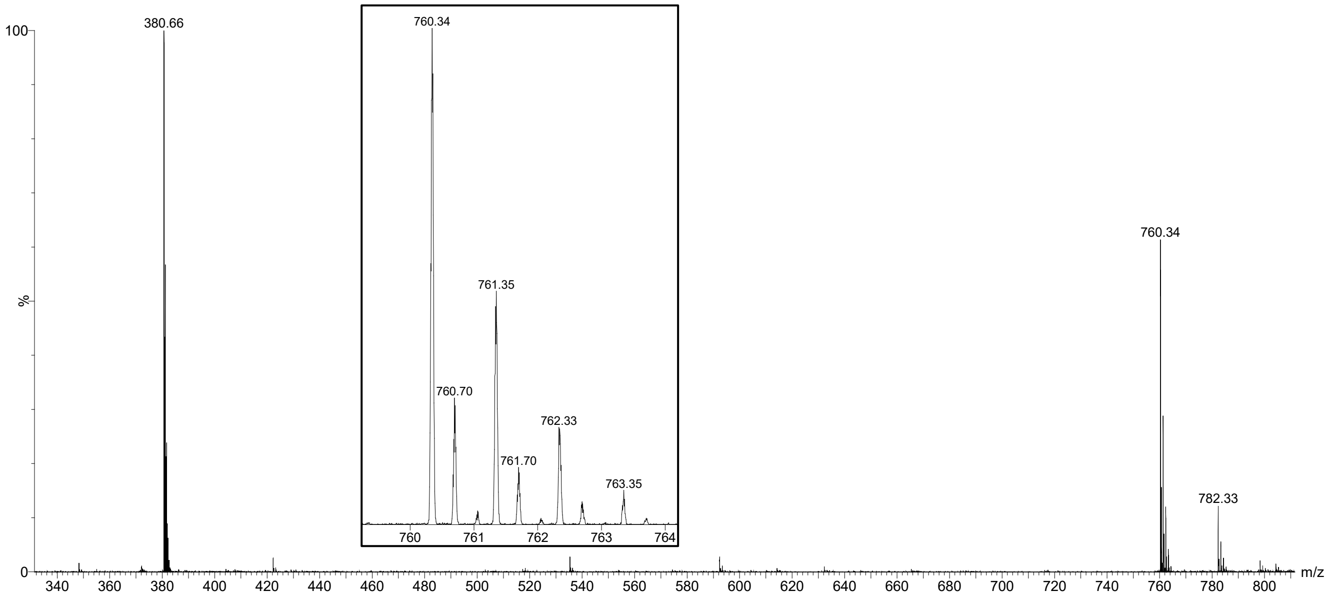

Supplementary Figure 27. *Mass spectrum of the monomer* $\boldsymbol{A_1}$ *from Supplementary Figure 26. m/z calculated: 760.34 [M+H]$^+$, 380.67 [M+2H]$^{2+}$; m/z observed: 760.34 [M+H]$^+$, 380.66 [M+2H]$^{2+}$. Inset: isotopic profile at m/z 760.34.*

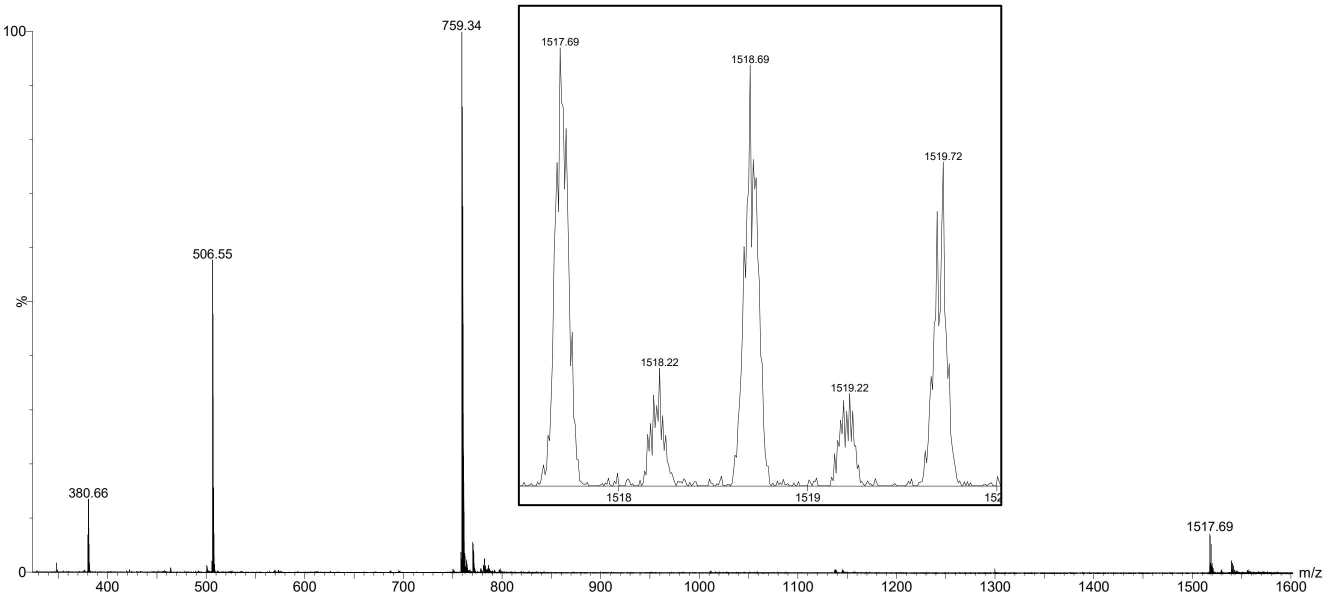

Supplementary Figure 28*. Mass spectrum of the linear dimer* ***A_2*** *from Supplementary Figure 26. m/z calculated: 1517.68 [M+H]$^+$, 759.34 [M+2H]$^{2+}$, 506.56 [M+3H]$^{3+}$, 380.17 [M+4H]$^{4+}$; m/z observed: 1517.69 [M+H]$^+$, 759.34 [M+2H]$^{2+}$, 506.55 [M+3H]$^{3+}$, 380.66 [M+4H]$^{4+}$. Inset: isotopic profile at m/z 1517.69.*

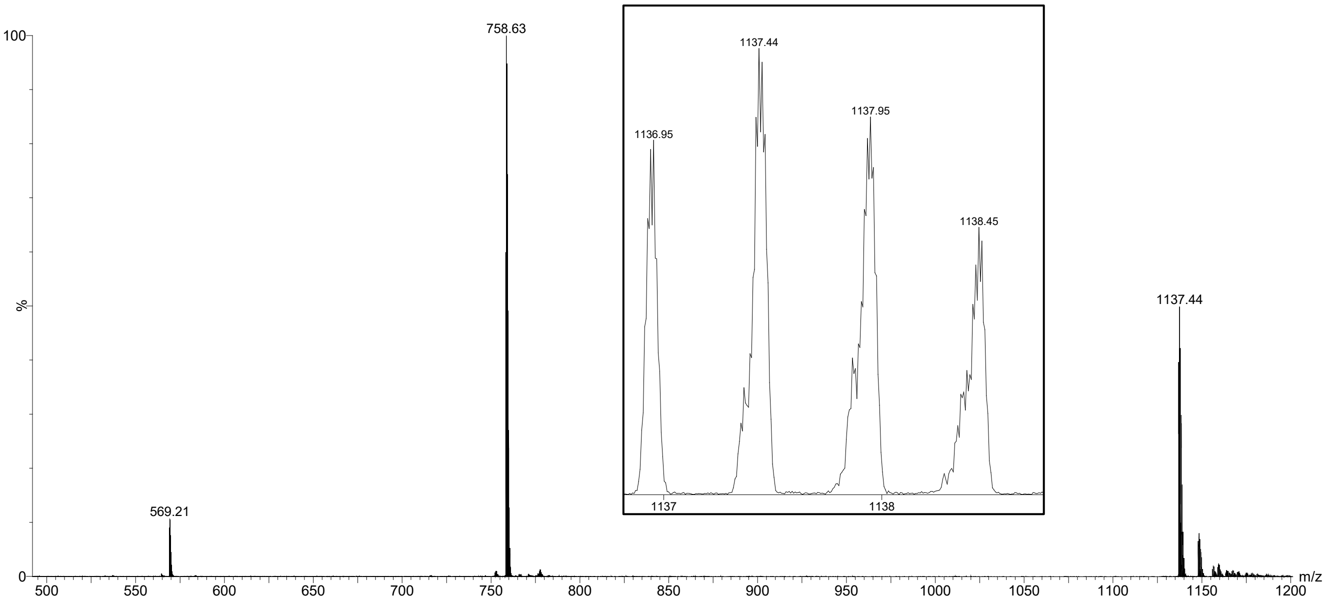

Supplementary Figure 29*. Mass spectrum of the cyclic* ***A_3*** *from Supplementary Figure 26. m/z calculated: 1137.01 [M+2H]$^{2+}$, 758.34 [M+3H]$^{3+}$, 569.00 [M+4H]$^{4+}$; m/z observed: 1136.95 [M+2H]$^{2+}$, 758.63 [M+3H]$^{3+}$, 569.21 [M+4H]$^{4+}$. Inset: isotopic profile at m/z 1136.95.*

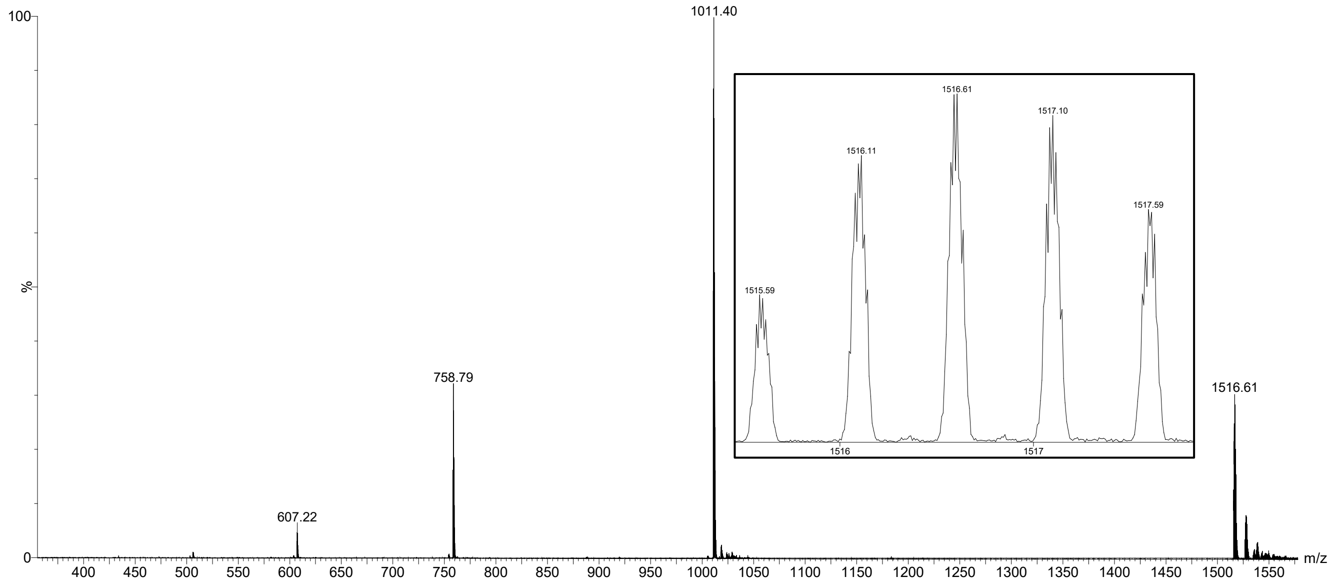

***Supplementary Figure 30**. Mass spectrum of the cyclic **A_4** from Supplementary Figure 26. m/z calculated: 1515.68 $[M+2H]^{2+}$, 1010.79 $[M+3H]^{3+}$, 758.34 $[M+4H]^{4+}$, 606.87 $[M+5H]^{5+}$. m/z observed: 1515.59 $[M+2H]^{2+}$, 1010.72 $[M+3H]^{3+}$, 758.29 $[M+4H]^{4+}$, 606.84 $[M+5H]^{5+}$. Inset: isotopic profile at m/z 1515.59.*

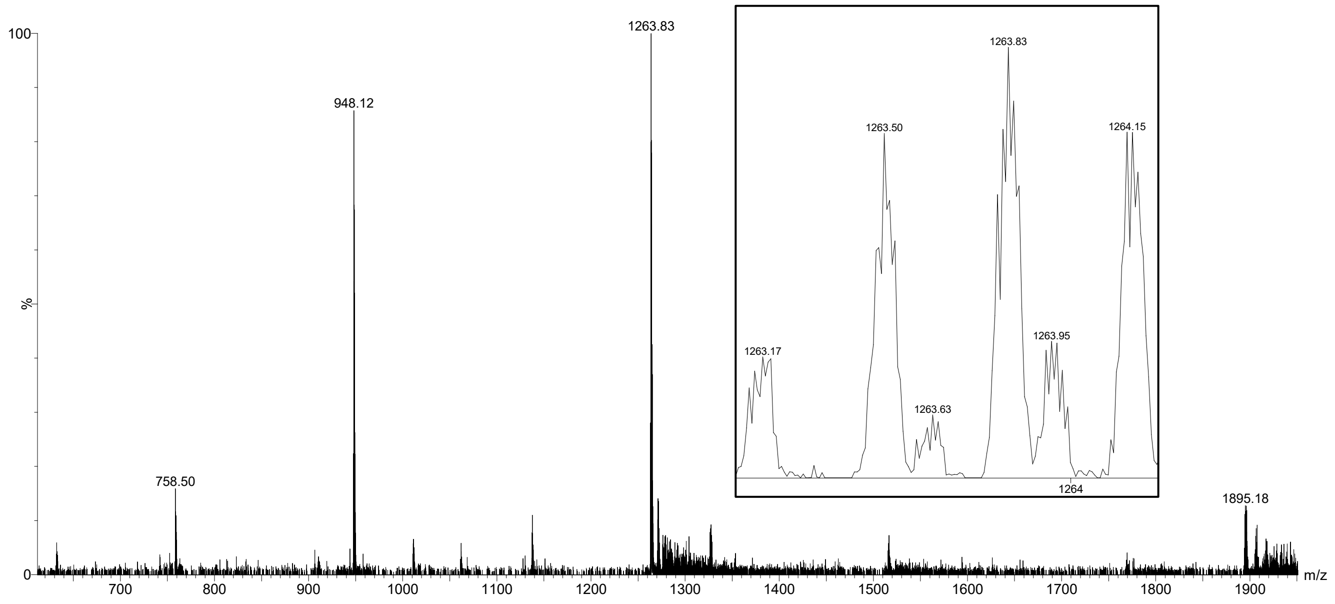

***Supplementary Figure 31**. Mass spectrum of the cyclic **A_5** from Supplementary Figure 26. m/z calculated: 1894.35 $[M+2H]^{2+}$, 1263.23 $[M+3H]^{3+}$, 947.67 $[M+4H]^{4+}$, 758.34 $[M+5H]^{5+}$; m/z observed: 1894.24 $[M+2H]^{2+}$, 1263.17 $[M+3H]^{3+}$, 947.61 $[M+4H]^{4+}$, 758.27 $[M+5H]^{5+}$. Inset: isotopic profile at m/z 1263.17.*

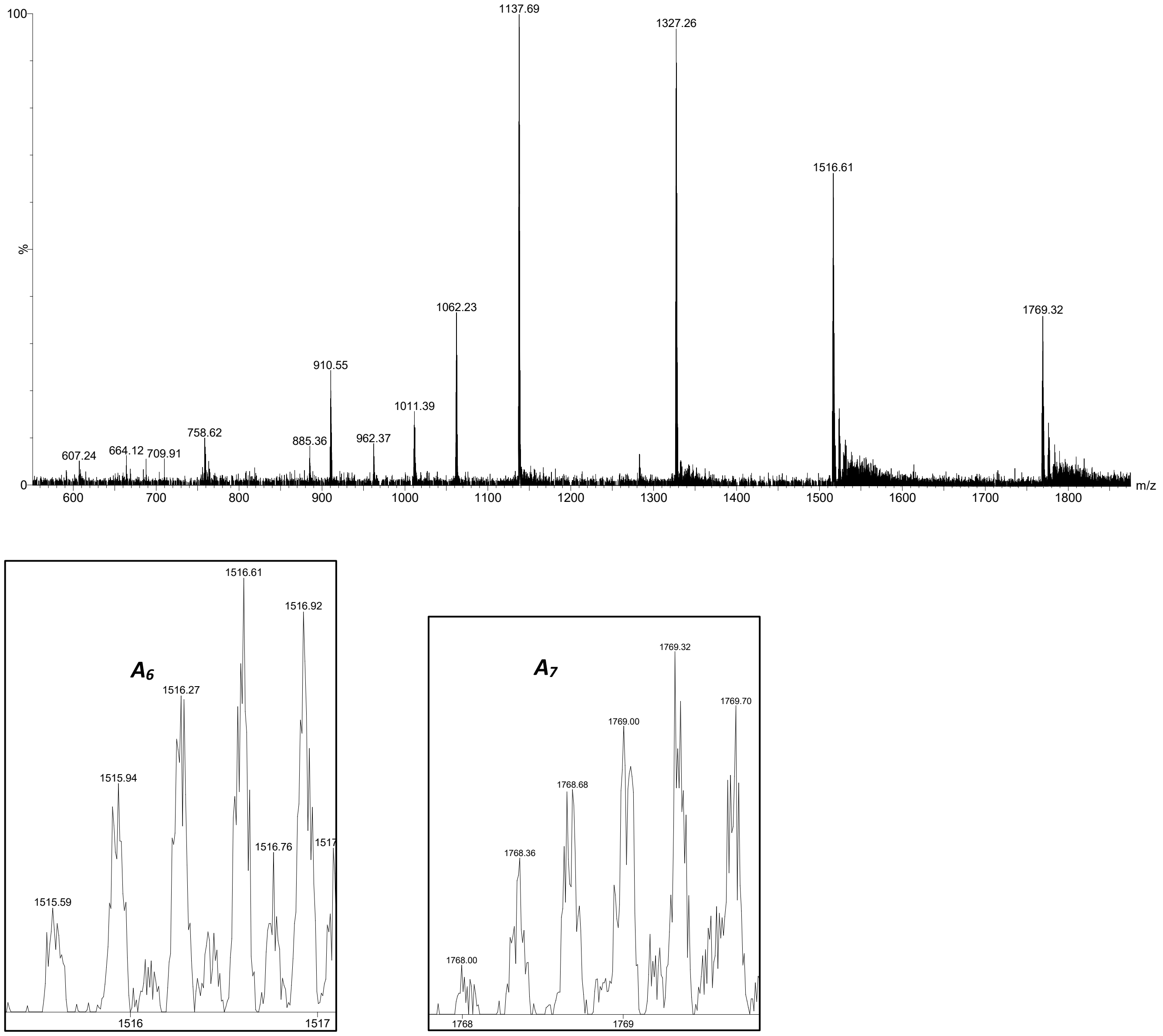

Supplementary Figure 32. *Mass spectrum of the cyclic **A_6** and **A_7** from Supplementary Figure 26. **A_6** m/z calculated: 1515.68 $[M+3H]^{3+}$, 1137.01 $[M+4H]^{4+}$, 909.80 $[M+5H]^{5+}$; **A_6** m/z observed: 1515.59 $[M+3H]^{3+}$, 1136.95 $[M+4H]^{4+}$, 909.73 $[M+5H]^{5+}$. Inset: isotopic profile at m/z 1515.59. **A_7** m/z calculated: 1768.13 $[M+3H]^{3+}$, 1326.34 $[M+4H]^{4+}$, 1061.27 $[M+5H]^{5+}$; **A_7** m/z observed: 1768.00 $[M+3H]^{3+}$, 1326.26 $[M+4H]^{4+}$, 1061.20 $[M+5H]^{5+}$. Inset: isotopic profile at m/z 1768.00.*

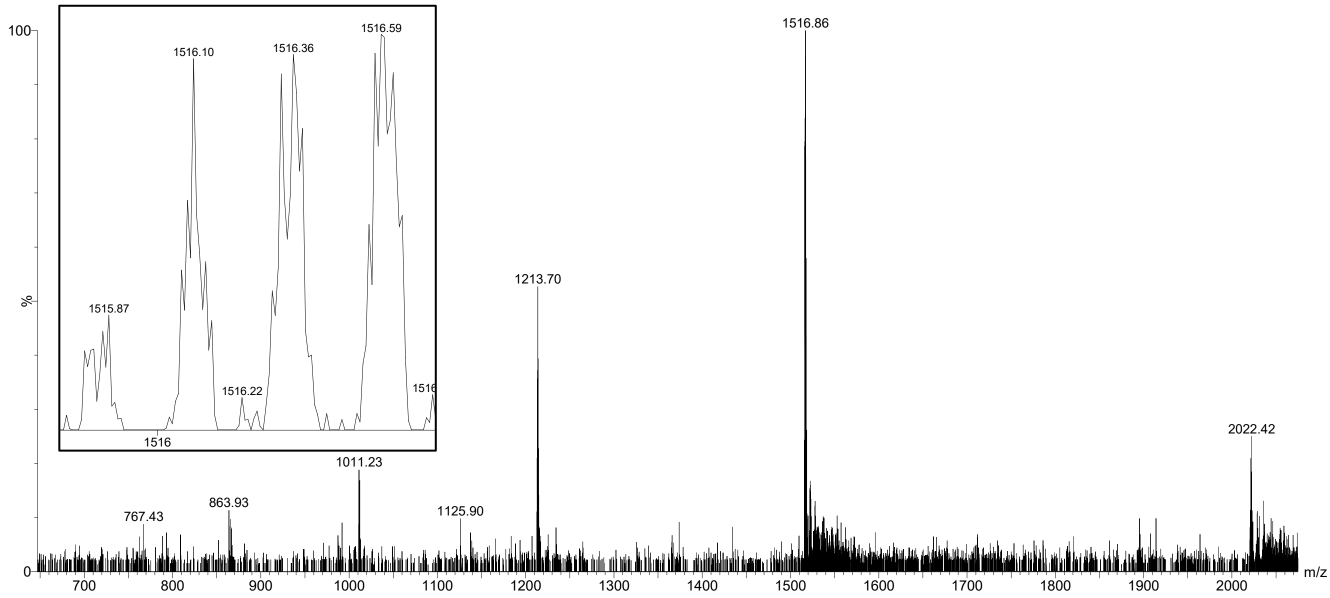

Supplementary Figure 33. *Mass spectrum of the cyclic* $\boldsymbol{A_8}$ *from Supplementary Figure 26. m/z calculated: 2020.57 [M+3H]*$^{3+}$*, 1515.68 [M+4H]*$^{4+}$*, 1212.74 [M+5H]*$^{5+}$*, 1010.79 [M+6H]*$^{6+}$*; m/z observed: 2020.42 [(M+3H]*$^{3+}$*, 1515.87 [(M+4H]*$^{4+}$*, 1212.89 [(M+5H]*$^{5+}$*, 1011.06 [M+6H]*$^{6+}$*. Inset: isotopic profile at m/z 1515.87.*

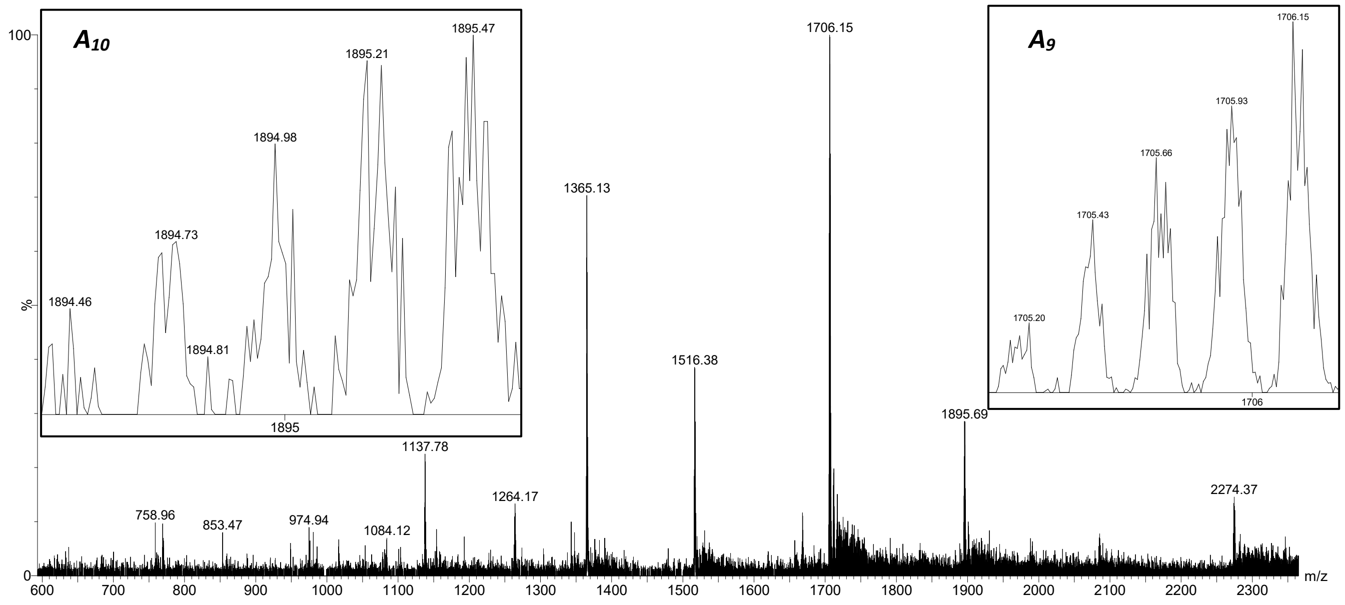

Supplementary Figure 34. *Mass spectrum of the cyclic* $\boldsymbol{A_9}$ *and* $\boldsymbol{A_{10}}$ *from Supplementary Figure 26.* $\boldsymbol{A_9}$ *m/z calculated: 2273.02 [M+3H]*$^{3+}$*, 1705.01 [M+4H]*$^{4+}$*, 1364.21 [M+5H]*$^{5+}$*, 1137.01 [M+6H]*$^{6+}$*;* $\boldsymbol{A_9}$ *m/z observed: 2273.34 [M+3H]*$^{3+}$*, 1705.20 [M+4H]*$^{4+}$*, 1364.11 [M+5H]*$^{5+}$*, 1137.12 [M+6H]*$^{6+}$*. Inset: isotopic profile at m/z 1705.20.* $\boldsymbol{A_{10}}$ *m/z calculated: 1894.35 [M+4H]*$^{4+}$*, 1515.68 [M+5H]*$^{5+}$*, 1263.23 [M+6H]*$^{6+}$*;* $\boldsymbol{A_{10}}$ *m/z observed: 1894.46 [M+4H]*$^{4+}$*, 1515.80 [M+5H]*$^{5+}$*, 1263.53 [M+6H]*$^{6+}$*. Inset: isotopic profile at m/z 1894.46.*

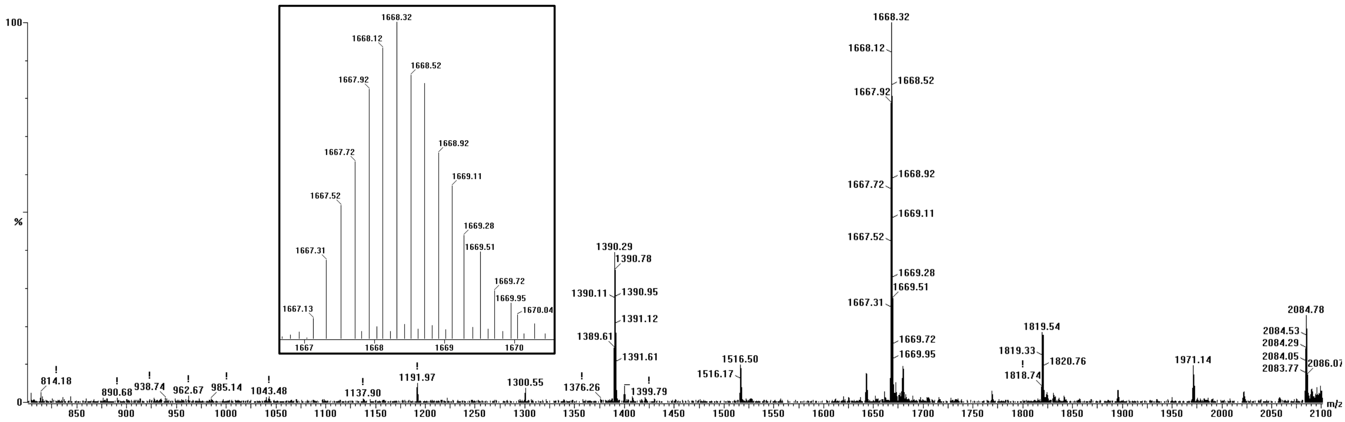

Supplementary Figure 35. Mass spectrum of the cyclic $\mathbf{A_{11}}$ from Supplementary Figure 26. $\mathbf{A_{11}}$ m/z calculated: 2083.69 $[M+3H]^{4+}$, 1667.15 $[M+5H]^{5+}$, 1389.46 $[M+6H]^{6+}$; $\mathbf{A_{11}}$ m/z observed: 2083.77 $[M+4H]^{4+}$, 1667.31 $[M+5H]^{5+}$, 1389.46 $[M+6H]^{6+}$. Inset: isotopic profile at m/z 1667.13.

3.4.2 Building block B

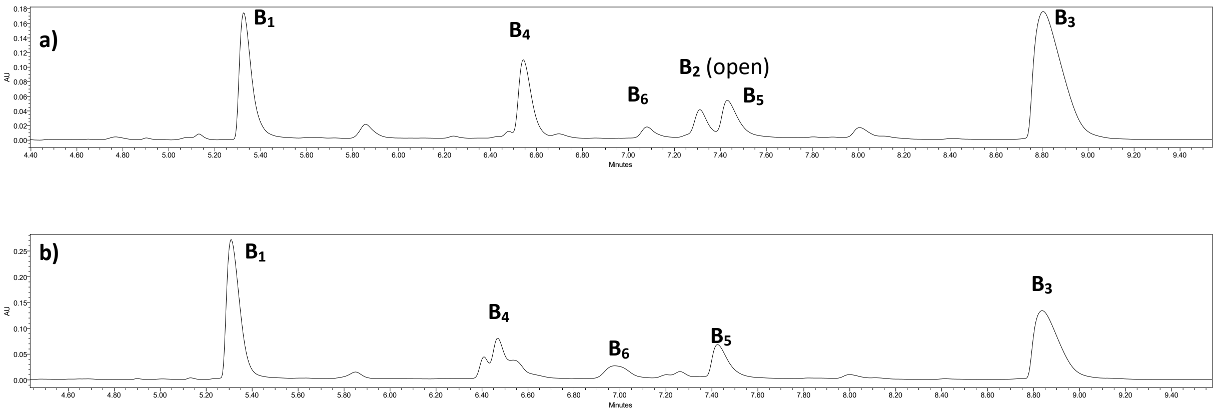

Supplementary Figure 36. Typical UPLC traces of libraries made from a) enantiopure **B** and b) racemic **B** (3.8 mM in borate buffer with 4M GuHCl, pH 8.1) under constant mechanical agitation. Because the same species in these two traces show mostly identical elution time and have the same mass, only one mass spectrum for each species is provided below.

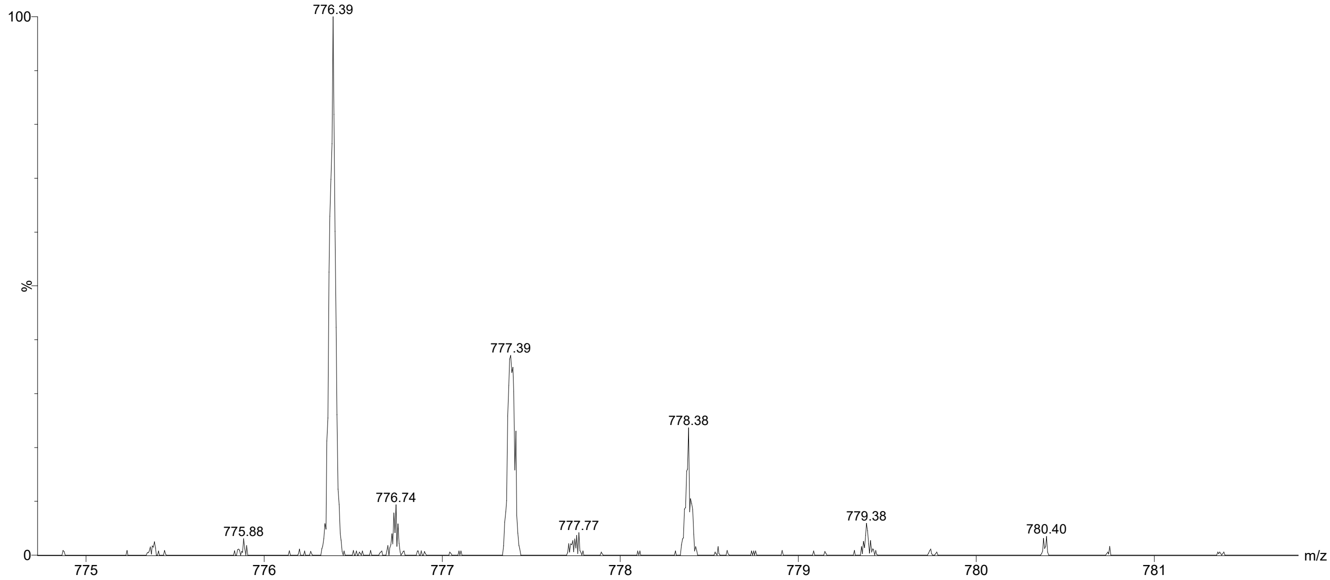

Supplementary Figure 37. *Mass spectrum of the monomer* ***B_1*** *from Supplementary Figure 36. m/z calculated: 776.34 [M+1H]$^+$; m/z observed: 776.39 [M+1H]$^+$.*

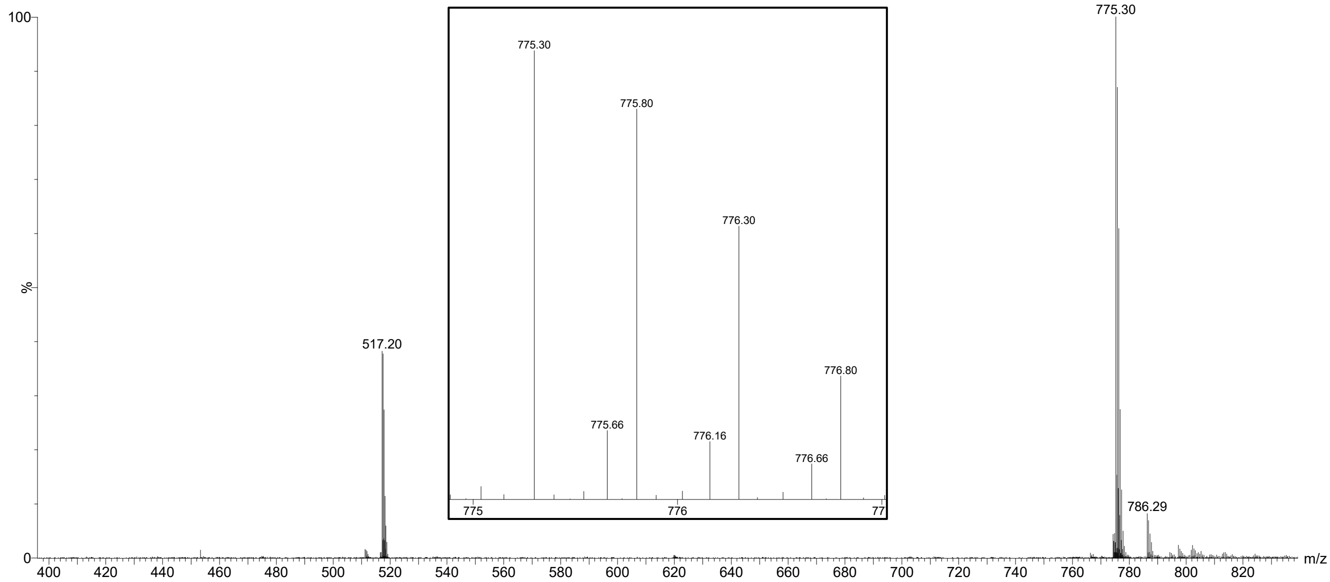

Supplementary Figure 38. *Mass spectrum of the dimer* ***B_2*** *from Supplementary Figure 36. m/z calculated: 775.34 [M+2H]$^{2+}$, 517.22 [M+3H]$^{3+}$; m/z observed: 775.30 [M+3H]$^{3+}$, 517.20 [M+4H]$^{4+}$. Inset: isotopic profile at m/z 775.30.*

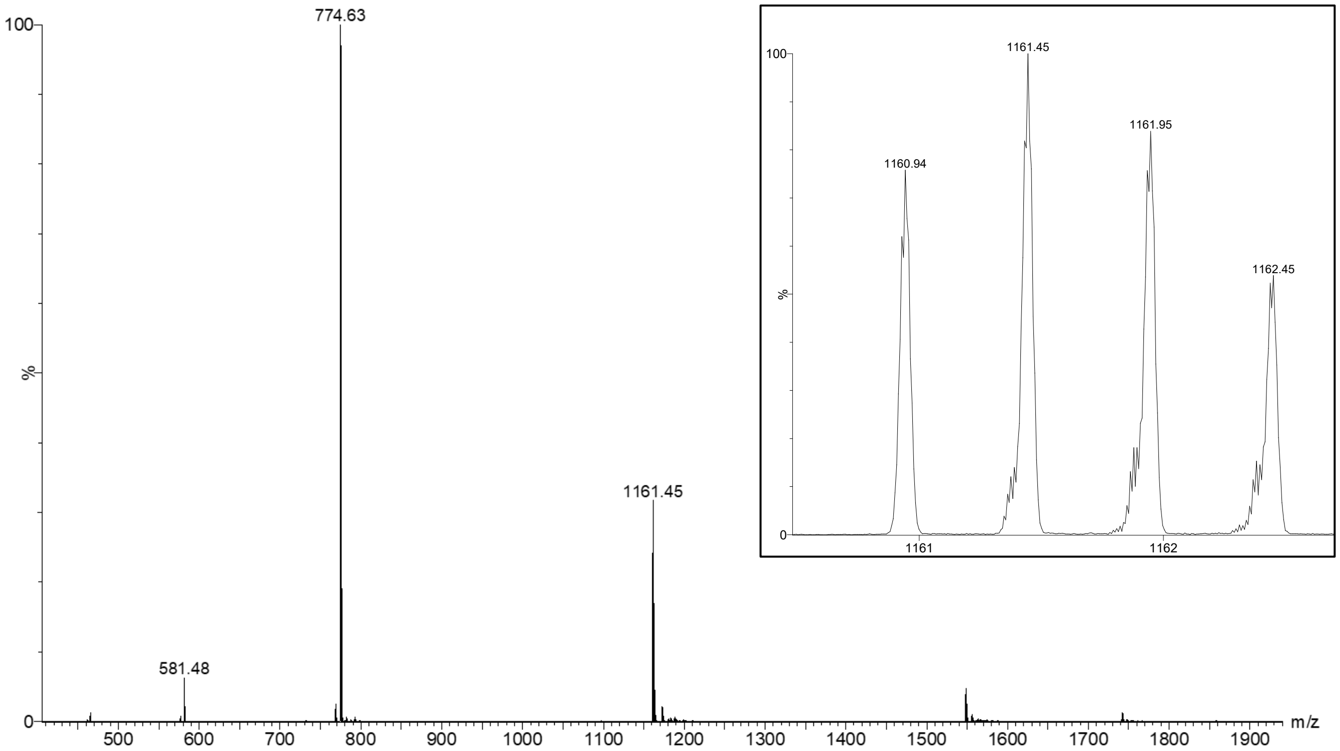

Supplementary Figure 39. *Mass spectrum of the cyclic **B_3** from Supplementary Figure 36. m/z calculated: 1161.01 [M+2H]$^{2+}$, 774.34 [M+3H]$^{3+}$, 581.00 [M+4H]$^{4+}$; m/z observed: 1160.94 [M+2H]$^{2+}$, 774.30 [M+3H]$^{3+}$, 580.98 [M+4H]$^{4+}$. Inset: isotopic profile at m/z 1160.94.*

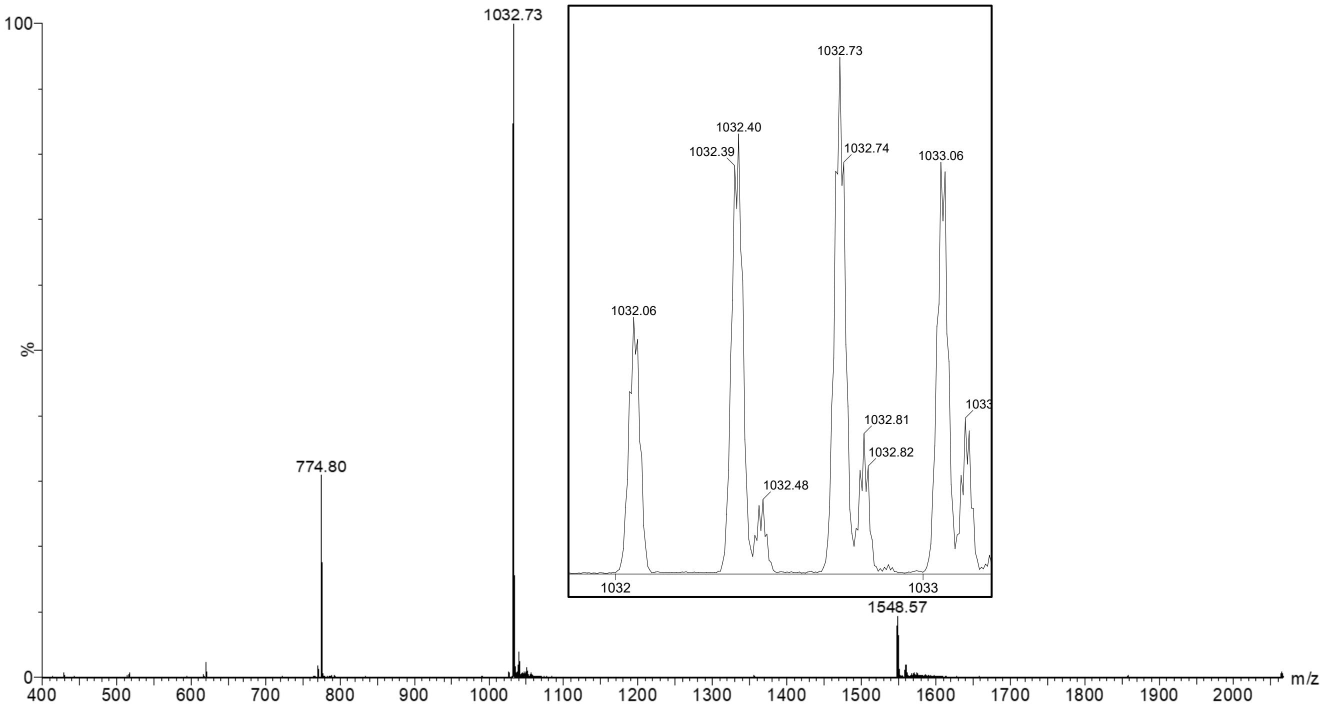

Supplementary Figure 40. *Mass spectrum of the cyclic **B_4** from Supplementary Figure 36. m/z calculated: 1547.68 [M+2H]$^{2+}$, 1032.12[M+3H]$^{3+}$, 774.34 [M+4H]$^{4+}$; m/z observed: 1547.57 [M+2H]$^{2+}$, 1032.06 [M+3H]$^{3+}$, 774.30 [M+4H]$^{4+}$. Inset: isotopic profile at m/z 1032.06.*

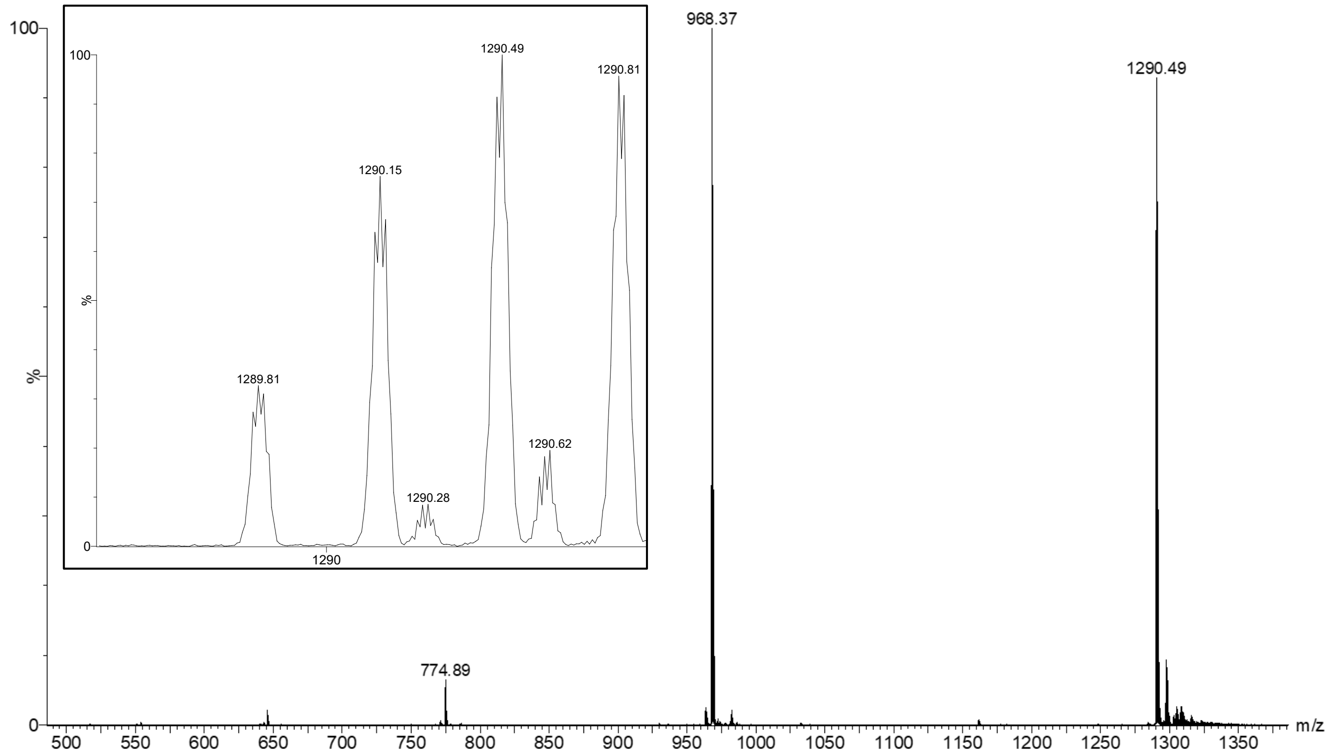

Supplementary Figure 41. *Mass spectrum of the cyclic* $\boldsymbol{B_5}$ *from Supplementary Figure 36. m/z calculated: 1289.90 [M+3H]*$^{3+}$*, 967.67 [M+4H]*$^{4+}$*, 774.34 [M+5H]*$^{5+}$*; m/z observed: 1289.91 [M+3H]*$^{3+}$*, 967.62 [M+4H]*$^{4+}$*, 774.30 [M+5H]*$^{5+}$*. Inset: isotopic profile at m/z 1289.91.*

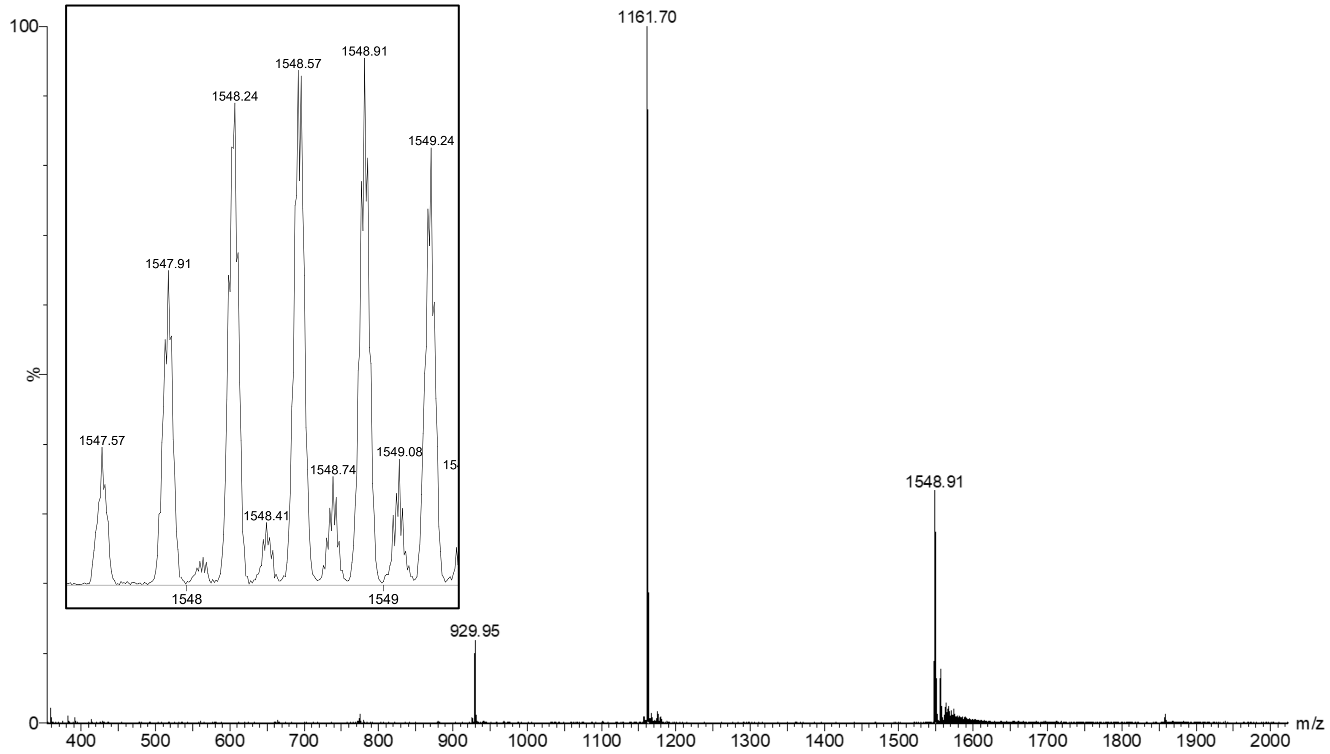

Supplementary Figure 42. *Mass spectrum of the cyclic* $\boldsymbol{B_6}$ *from Supplementary Figure 36. m/z calculated: 1547.68 [M+3H]*$^{3+}$*, 1161.01 [M+4H]*$^{4+}$*, 929.00 [M+5H]*$^{5+}$*; m/z observed: 1547.57 [M+3H]*$^{3+}$*, 1160.94 [M+4H]*$^{4+}$*, 928.96 [M+5H]*$^{5+}$*. Inset: isotopic profile at m/z 1547.57.*

3.4.3 Building block C

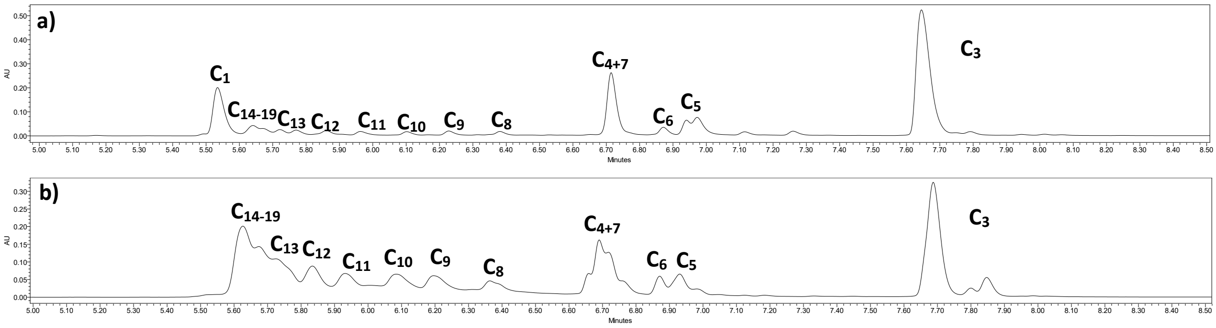

***Supplementary Figure 43**. Typical UPLC traces of libraries made from a) enantiopure **C** and b) racemic **C** (3.8 mM in borate buffer with 4M GuHCl, pH 8.1) under constant mechanical agitation. Because the same species in these two traces show mostly identical elution time and have the same mass, only one mass spectrum for each species is provided below.*

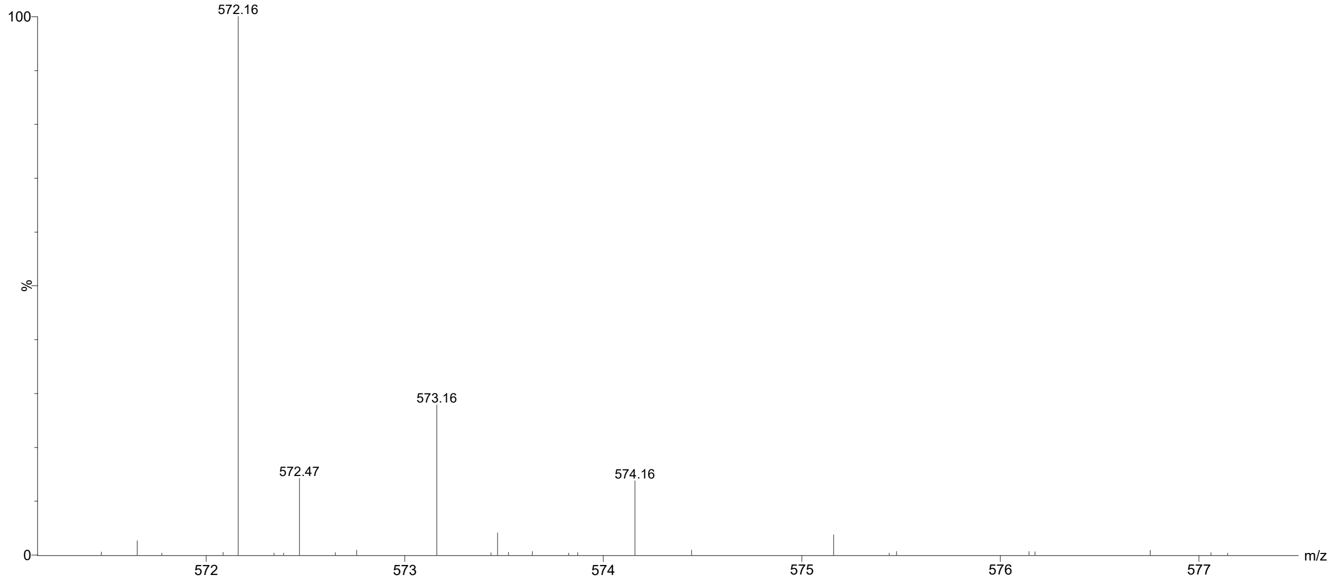

***Supplementary Figure 44**. Mass spectrum of the monomer $\boldsymbol{C_1}$ from Supplementary Figure 43. m/z calculated: 572.21 $[M+1H]^+$; m/z observed: 572.16 $[M+1H]^+$.*

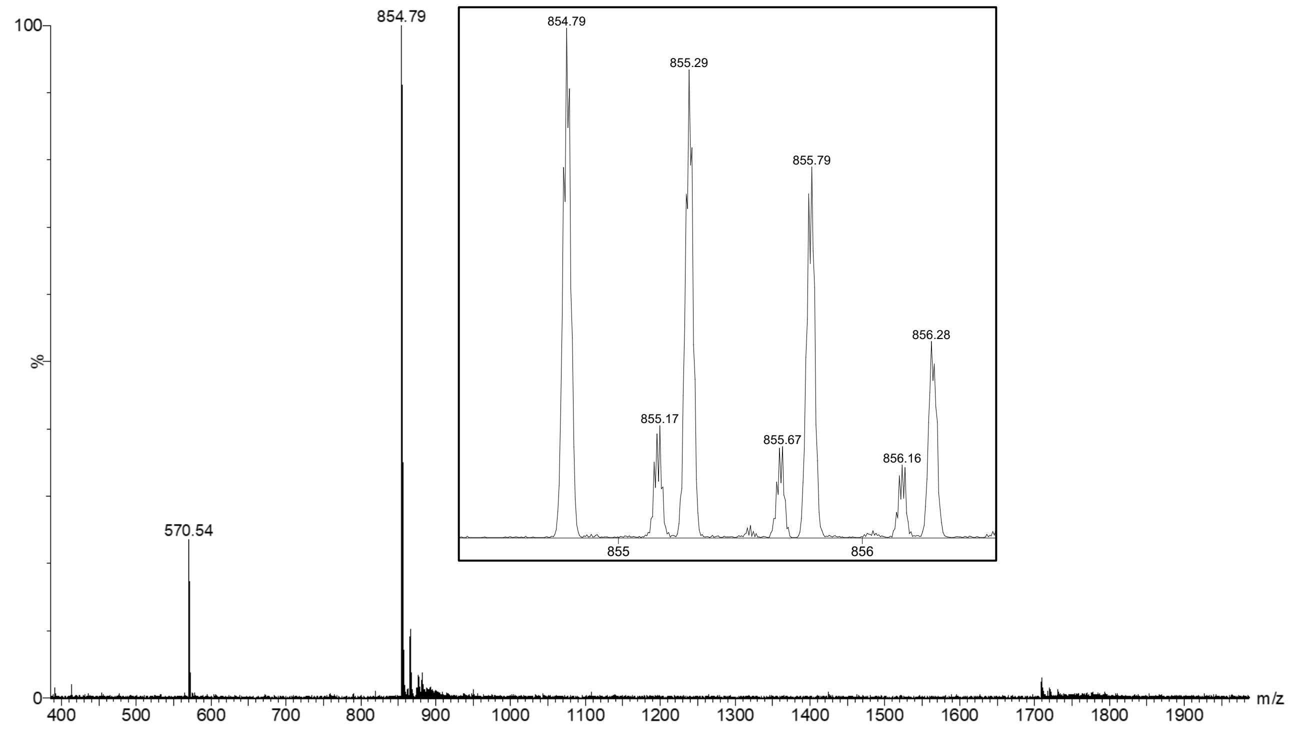

Supplementary Figure 45. *Mass spectrum of the cyclic **C_3** from Supplementary Figure 43. m/z calculated: 854.81 $[M+2H]^{2+}$, 570.21 $[M+3H]^{3+}$; m/z observed: 854.79 $[M+2H]^{2+}$, 570.19 $[M+3H]^{3+}$. Inset: isotopic profile at m/z 854.79.*

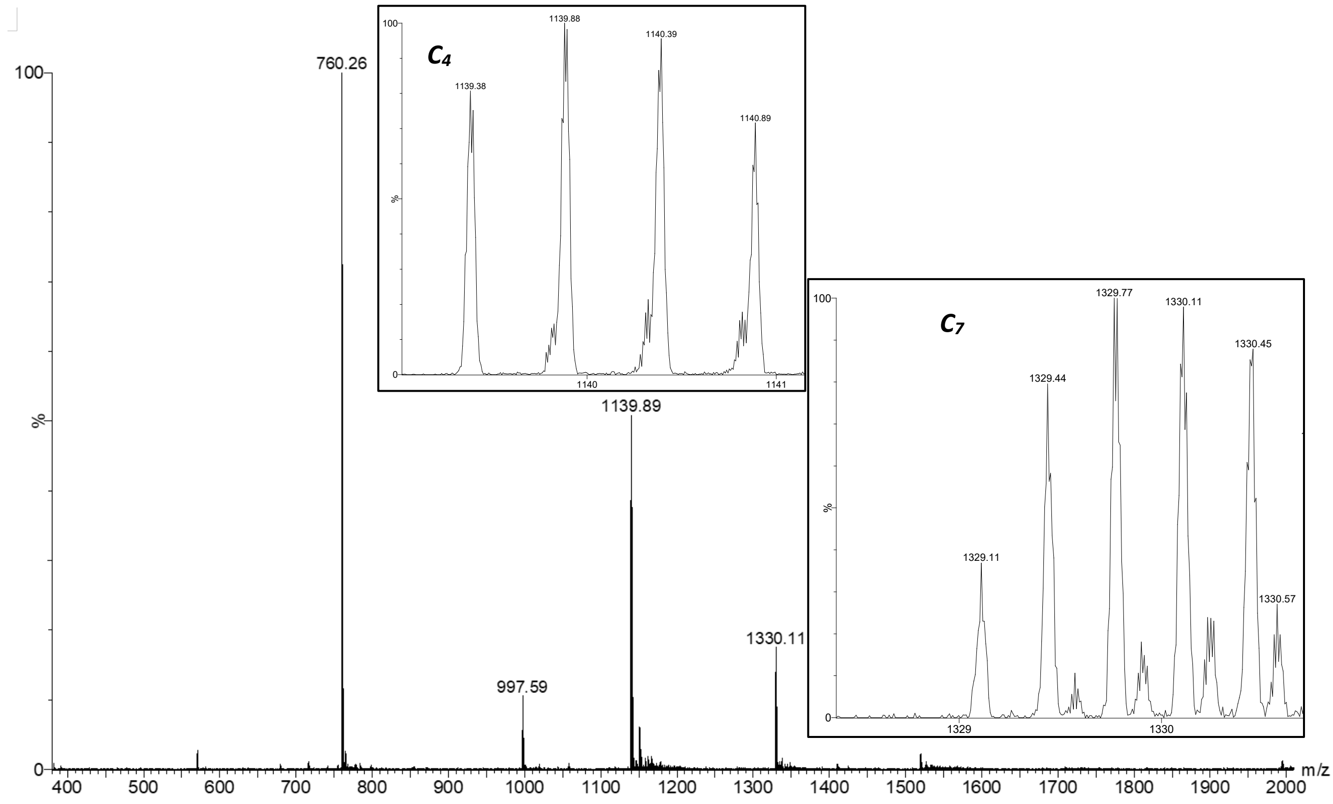

Supplementary Figure 46. *Mass spectrum of the cyclic **C_4** and **C_7** from Supplementary Figure 43. **C_4** m/z calculated: 1139.42 $[M+2H]^{2+}$, 759.94 $[M+3H]^{3+}$; m/z observed: 1139.38 $[M+2H]^{2+}$, 759.92 $[M+3H]^{3+}$. Inset: isotopic profile at m/z 1139.38. **C_7** m/z calculated: 1329.16 $[M+3H]^{3+}$, 997.11 $[M+4H]^{4+}$; m/z observed: 1329.11 $[M+3H]^{3+}$, 997.09 $[M+4H]^{4+}$. Inset: isotopic profile at m/z 1329.11.*

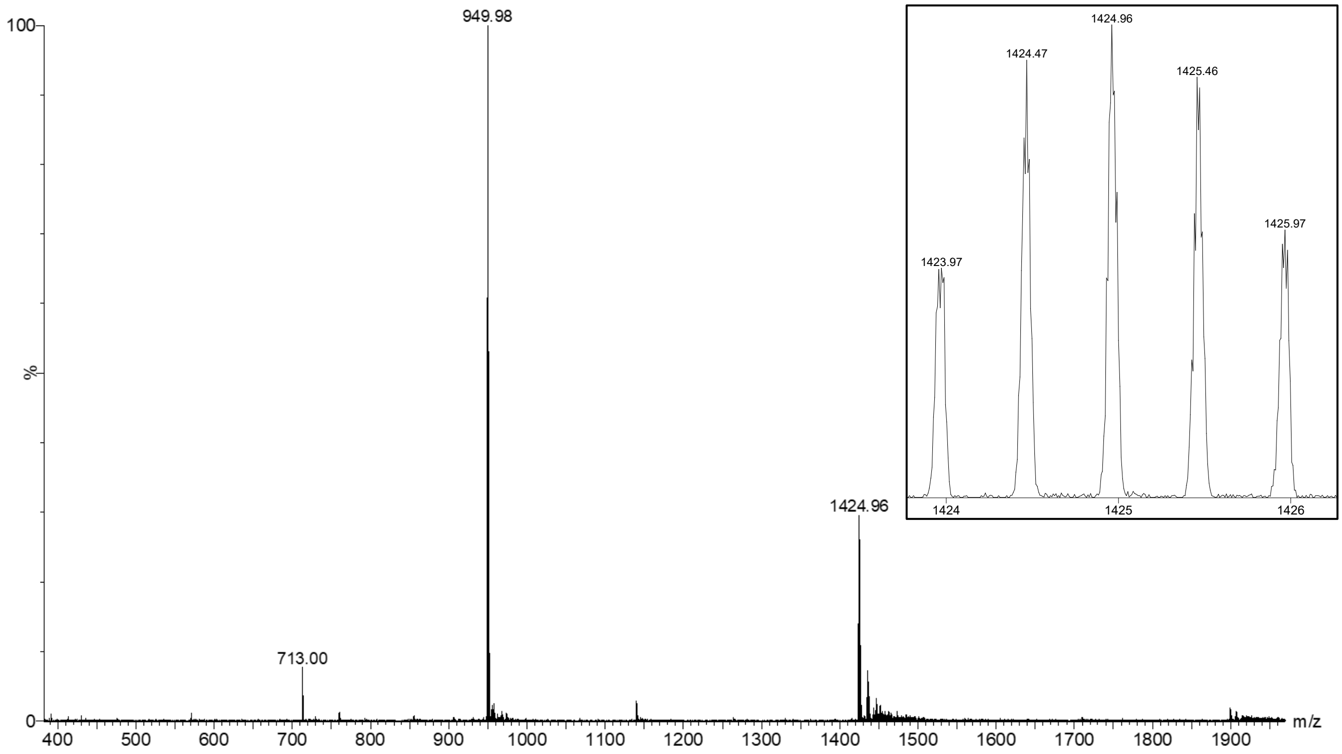

Supplementary Figure 47. *Mass spectrum of the cyclic* $\boldsymbol{C_5}$ *from Supplementary Figure 43. m/z calculated: 1424.03 [M+2H]*$^{2+}$*, 949.68 [M+3H]*$^{3+}$*, 712.51 [M+4H]*$^{4+}$*; m/z observed: 1423.97 [M+2H]*$^{2+}$*, 949.66 [M+3H]*$^{3+}$*, 712.49 [M+4H]*$^{4+}$*. Inset: isotopic profile at m/z 1423.97.*

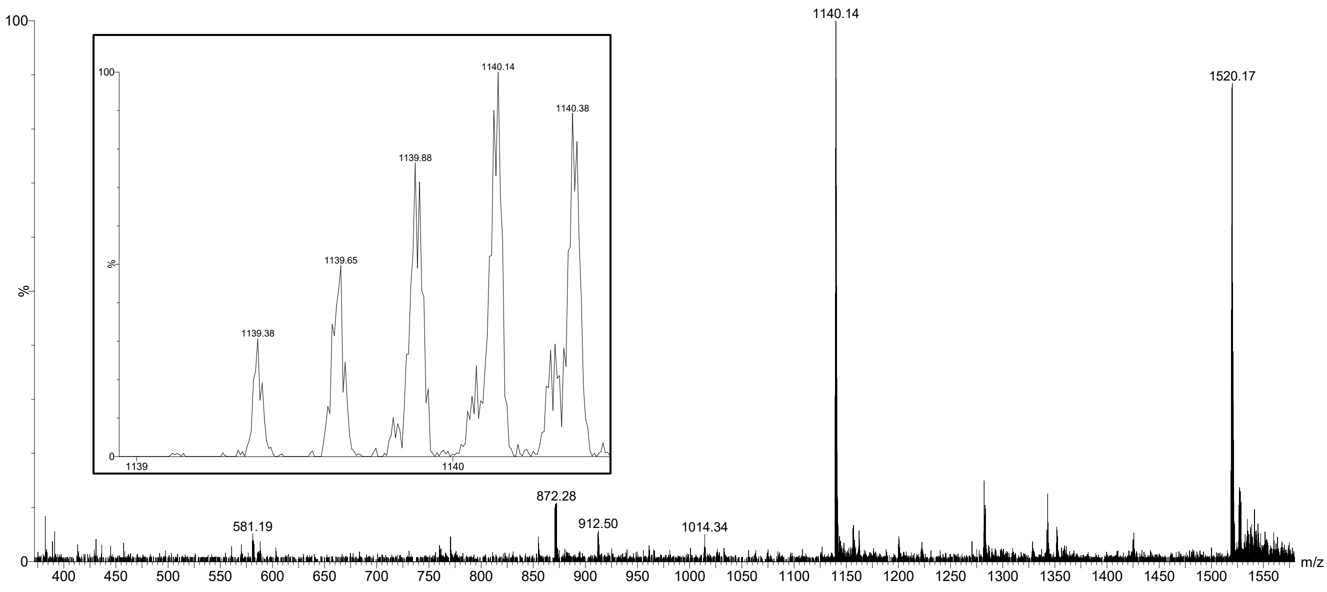

Supplementary Figure 48. *Mass spectrum of the cyclic* $\boldsymbol{C_8}$ *from Supplementary Figure 43. m/z calculated: 1518.89 [M+3H]*$^{3+}$*, 1139.42 [M+4H]*$^{4+}$*; m/z observed: 1518.82 [M+3H]*$^{3+}$*, 1139.38 [M+4H]*$^{4+}$*. Inset: isotopic profile at m/z 1139.38.*

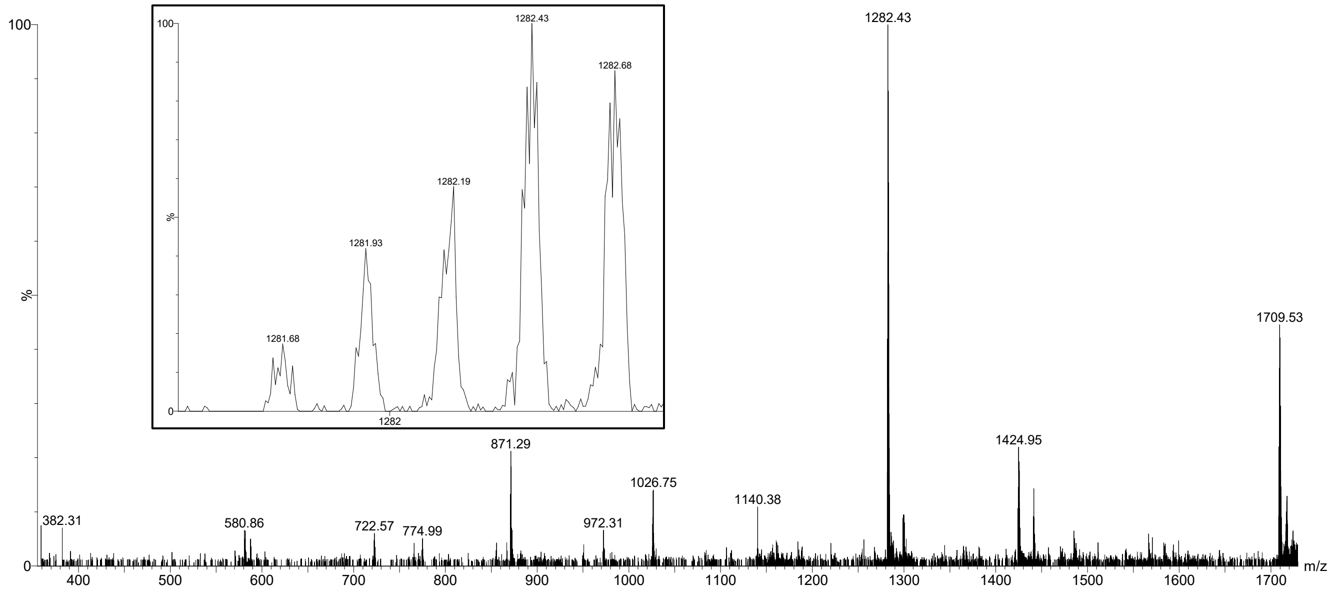

Supplementary Figure 49. *Mass spectrum of the cyclic* $\boldsymbol{C_9}$ *from Supplementary Figure 43. m/z calculated: 1708.63 [M+3H]*$^{3+}$*, 1281.72 [M+4H]*$^{4+}$*; m/z observed: 1708.55 [M+3H]*$^{3+}$*, 1281.68 [M+4H]*$^{4+}$*. Inset: isotopic profile at m/z 1281.68.*

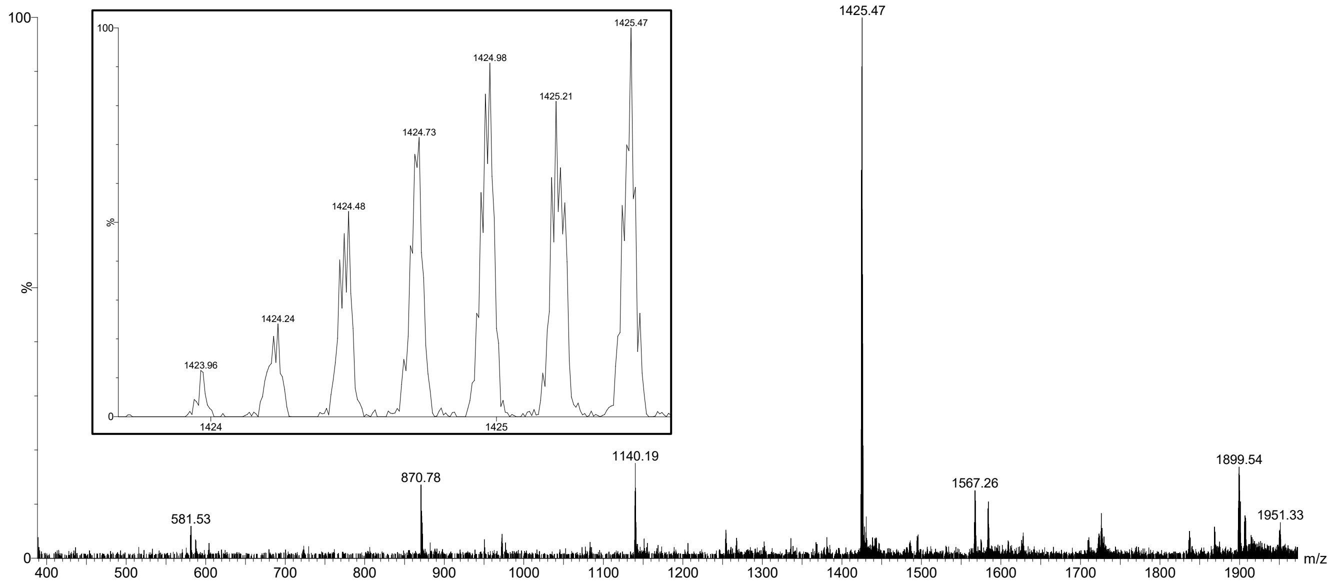

Supplementary Figure 50. *Mass spectrum of the cyclic* $\boldsymbol{C_{10}}$ *from Supplementary Figure 43. m/z calculated: 1898.37 [M+3H]*$^{3+}$*, 1424.02 [M+4H]*$^{4+}$*; m/z observed: 1898.20 [M+3H]*$^{3+}$*, 1423.96 [M+4H]*$^{4+}$*. Inset: isotopic profile at m/z 1423.96.*

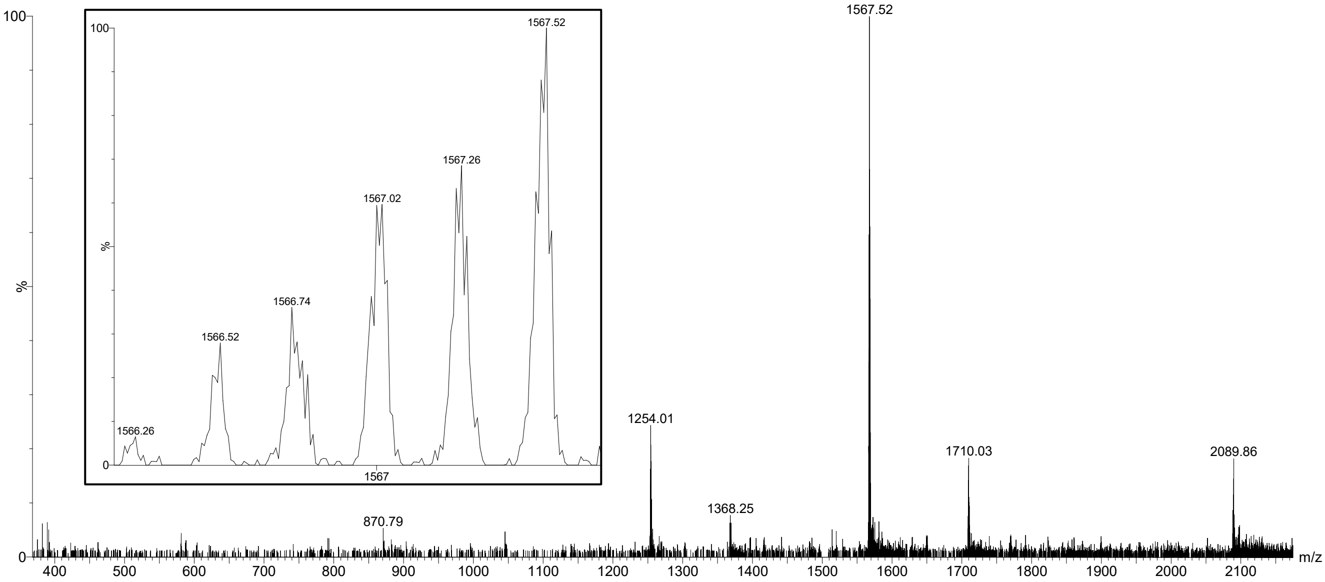

Supplementary Figure 51*. Mass spectrum of the cyclic* ***C_{11}*** *from Supplementary Figure 43. m/z calculated: 2088.10 $[M+3H]^{3+}$, 1566.32 $[M+4H]^{4+}$, 1253.26 $[M+5H]^{5+}$; m/z observed: 2088.22 $[M+3H]^{3+}$, 1566.26 $[M+4H]^{4+}$, 1253.43 $[M+5H]^{5+}$. Inset: isotopic profile at m/z 1566.26.*

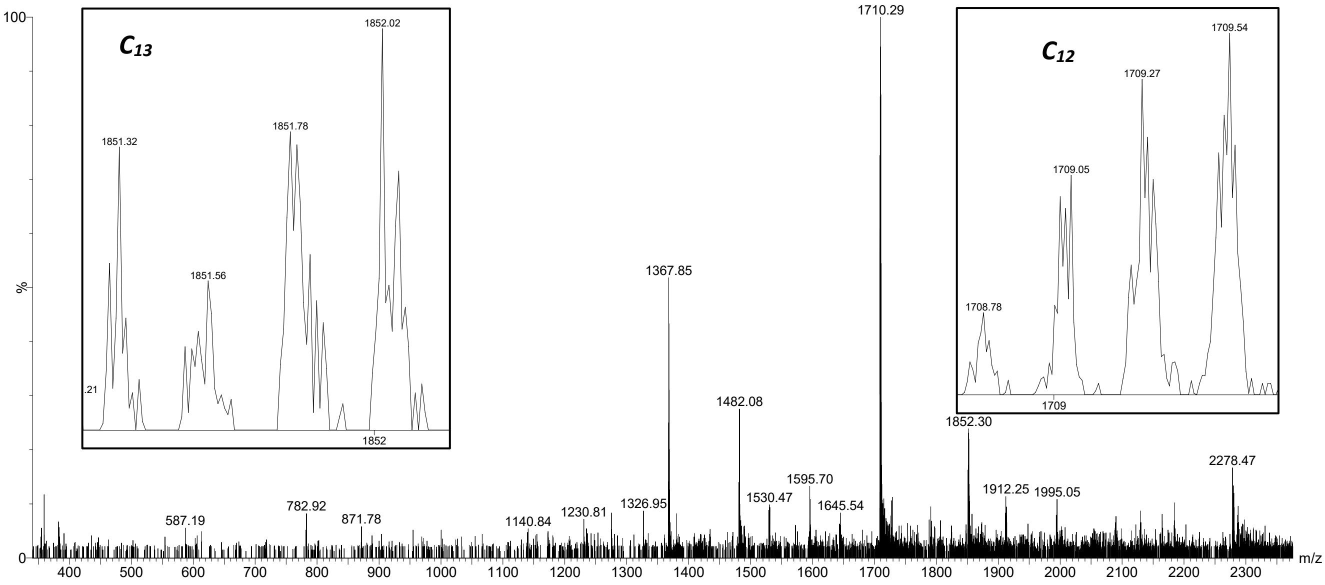

Supplementary Figure 52*. Mass spectrum of the cyclic* ***C_{12}*** *and* ***C_{13}*** *from Supplementary Figure 43.* ***C_{12}*** *m/z calculated: 2277.84 $[M+3H]^{3+}$, 1708.63 $[M+4H]^{4+}$, 1367.10 $[M+5H]^{5+}$;* ***C_{12}*** *m/z observed: 2277.69 $[M+3H]^{3+}$, 1708.78 $[M+4H]^{4+}$, 1367.04 $[M+5H]^{5+}$. Inset: isotopic profile at m/z 1708.78.* ***C_{13}*** *m/z calculated: 1850.93 $[M+3H]^{3+}$, 1480.94 $[M+4H]^{4+}$;* ***C_{13}*** *m/z observed: 1851.32 $[M+3H]^{3+}$, 1480.89 $[M+4H]^{4+}$. Inset: isotopic profile at m/z 1851.32.*

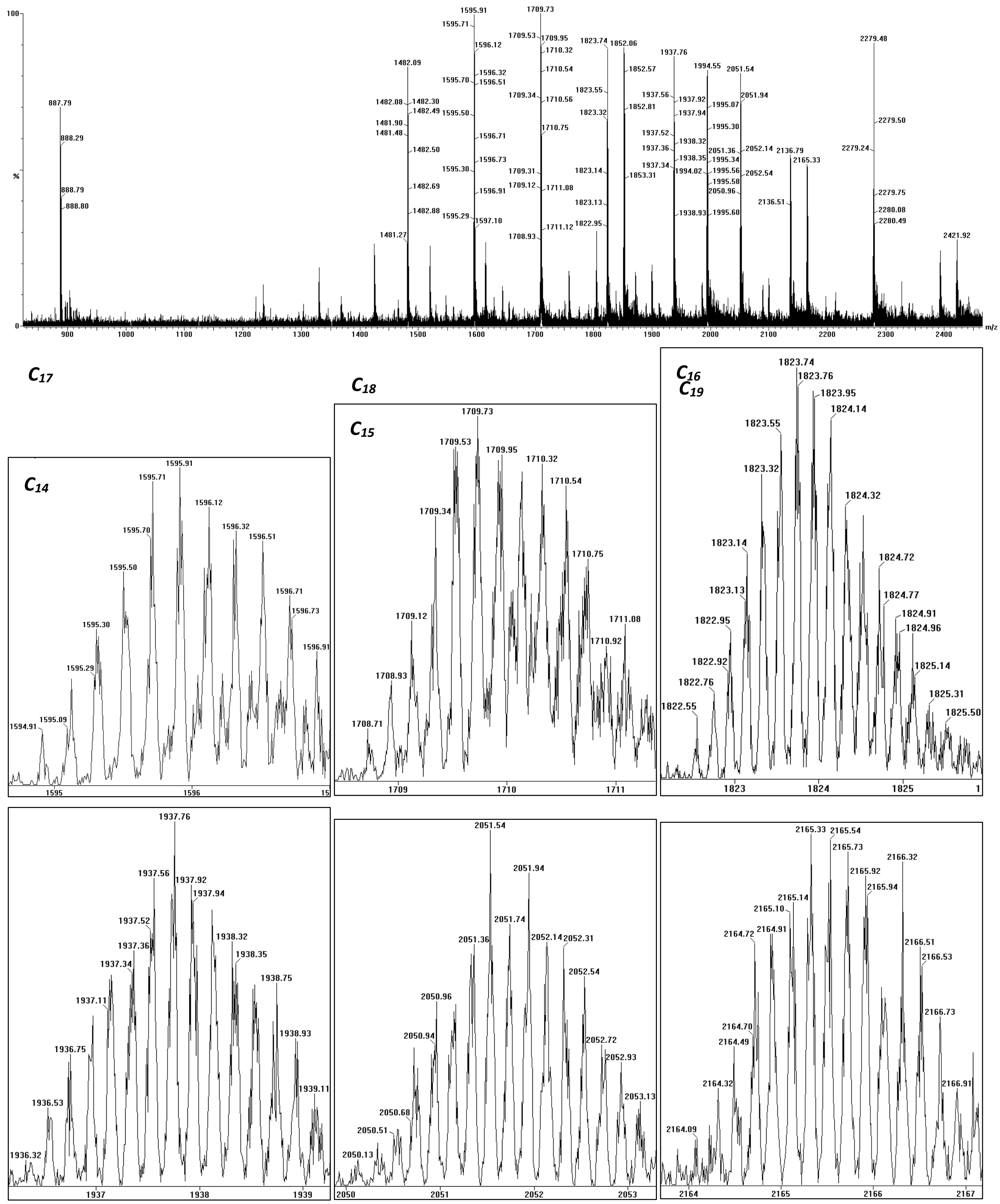

*Supplementary Figure 53. Mass spectrum of the cyclic **C_{14}** to **C_{19}** from Supplementary Figure 43. **C_{14}** m/z calculated: 1594.79 [M+5H]$^{5+}$, 1329.16 [M+6H]$^{6+}$; **C_{14}** m/z observed: 1594.91 [M+5H]$^{5+}$, 1329.29 [M+6H]$^{6+}$. Inset: isotopic profile at m/z 1594.91. **C_{15}** m/z calculated: 1708.63 [M+5H]$^{5+}$, 1424.03 [M+6H]$^{6+}$; **C_{15}** m/z observed: 1708.71 [M+5H]$^{5+}$, 1424.11 [M+6H]$^{6+}$. Inset: isotopic profile at m/z 1708.71. **C_{16}** m/z calculated: 1822.47 [M+5H]$^{5+}$, 1518.89 [M+6H]$^{6+}$; **C_{16}** m/z observed: 1822.55 [M+5H]$^{5+}$, 1518.96 [M+6H]$^{6+}$. Inset: isotopic profile at m/z 1822.55. **C_{17}** m/z calculated: 1936.31 [M+5H]$^{5+}$, 1613.76 [M+6H]$^{6+}$; **C_{17}** m/z observed: 1936.32 [M+5H]$^{5+}$, 1613.83 [M+6H]$^{6+}$. Inset:*

*isotopic profile at m/z 1936.32. **C_{18}** m/z calculated: 2050.16 [M+5H]$^{5+}$, 1708.63 [M+6H]$^{6+}$; **C_{18}** m/z observed: 2050.13 [M+5H]$^{5+}$, 1708.71 [M+6H]$^{6+}$. Inset: isotopic profile at m/z 2050.13. **C_{19}** m/z calculated: 2164.00 [M+5H]$^{5+}$, 1803.50 [M+6H]$^{6+}$; **C_{19}** m/z observed: 2164.09 [M+5H]$^{5+}$, 1803.56 [M+6H]$^{6+}$. Inset: isotopic profile at m/z 2164.09.*

3.4.4 Building block **D**

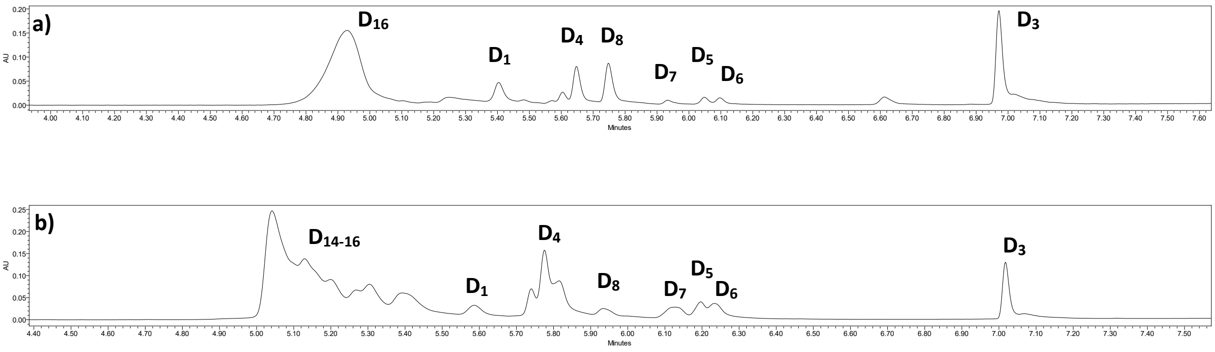

***Supplementary Figure 54**. Typical UPLC traces of libraries made from a) enantiopure **D** and b) racemic **D** (3.8 mM in borate buffer with 4M GuHCl, pH 8.1) under constant mechanical agitation. Because the same species in these two traces show mostly identical elution time and have the same mass, only one mass spectrum for each species is provided below.*

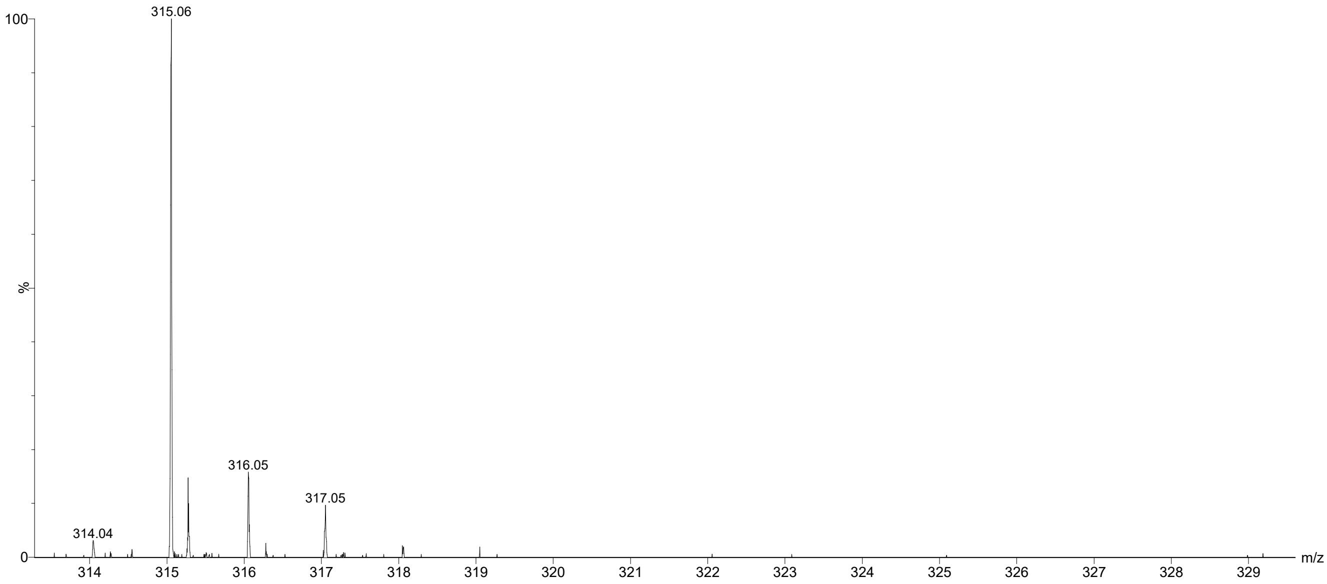

***Supplementary Figure 55**. Mass spectrum of the monomer **D_1** from Supplementary Figure 54. m/z calculated: 315.08 [M+1H]$^{+}$; m/z observed: 315.06 [M+1H]$^{+}$.*

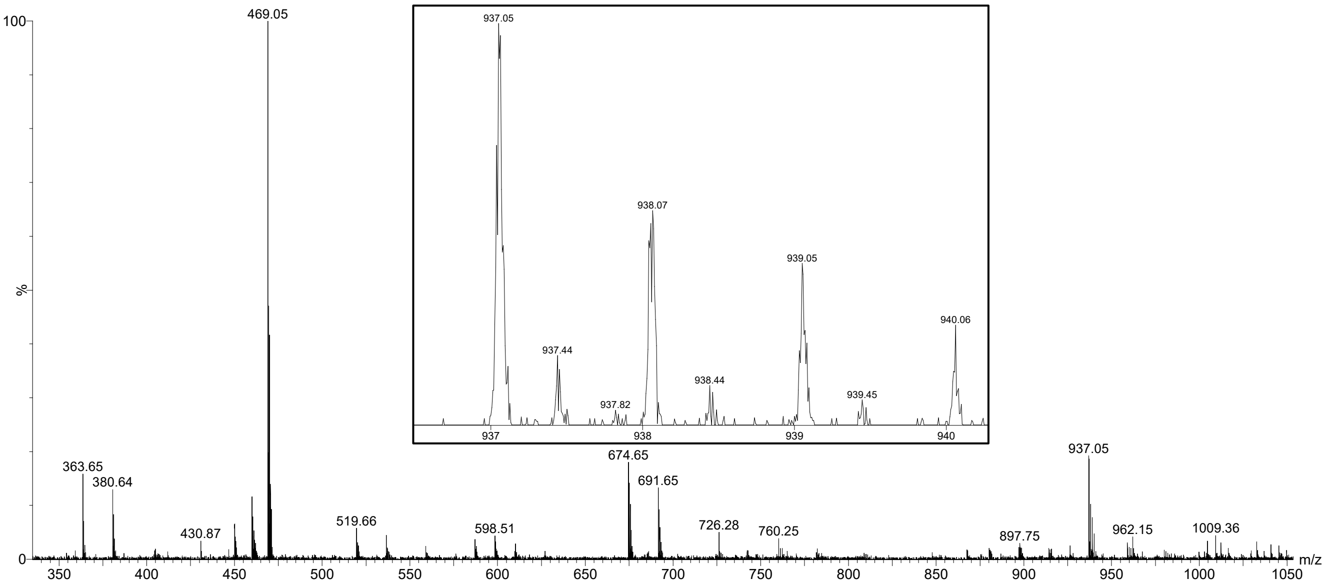

Supplementary Figure 56. *Mass spectrum of the cyclic* ***D₃*** *from Supplementary Figure 54. m/z calculated: 937.24 [M+1H]$^{+}$, 469.12 [M+2H]$^{2+}$; m/z observed: 937.05 [M+1H]$^{+}$, 469.05 [M+2H]$^{2+}$. Inset: isotopic profile at m/z 937.05.*

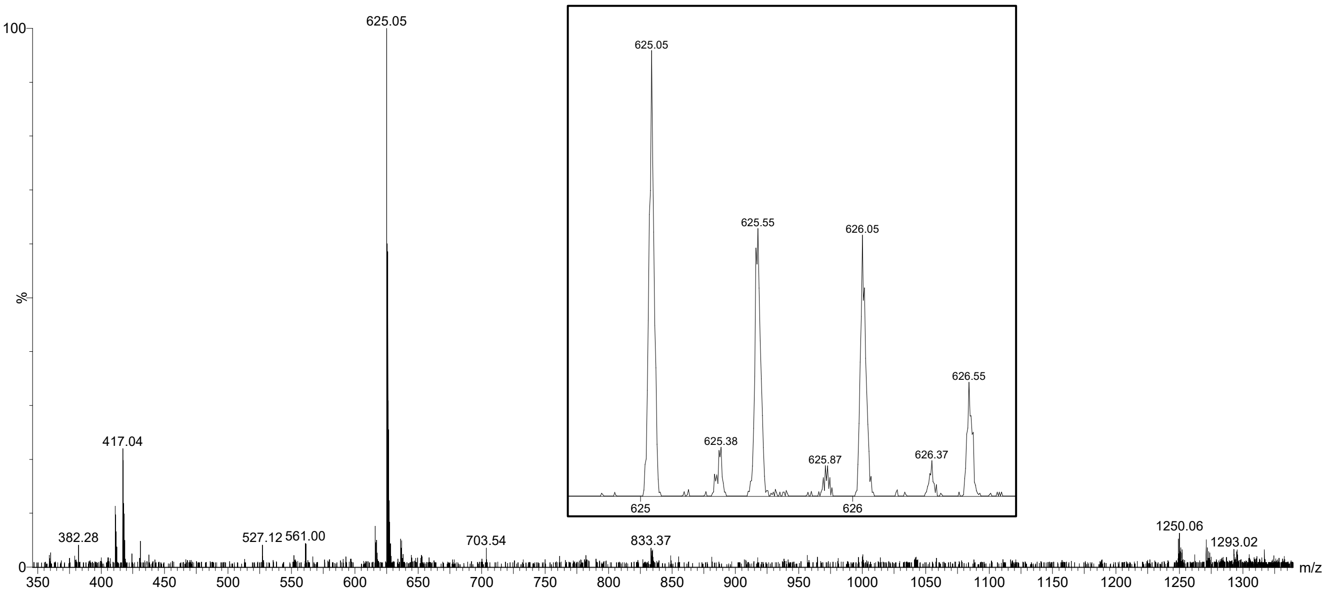

Supplementary Figure 57. *Mass spectrum of the cyclic* ***D₄*** *from Supplementary Figure 54. m/z calculated: 1249.32 [M+1H]$^{+}$, 625.16 [M+2H]$^{2+}$, 417.10 [M+3H]$^{3+}$; m/z observed: 1249.04 [M+1H]$^{+}$, 625.05 [M+2H]$^{2+}$, 417.04 [M+3H]$^{3+}$. Inset: isotopic profile at m/z 625.05.*

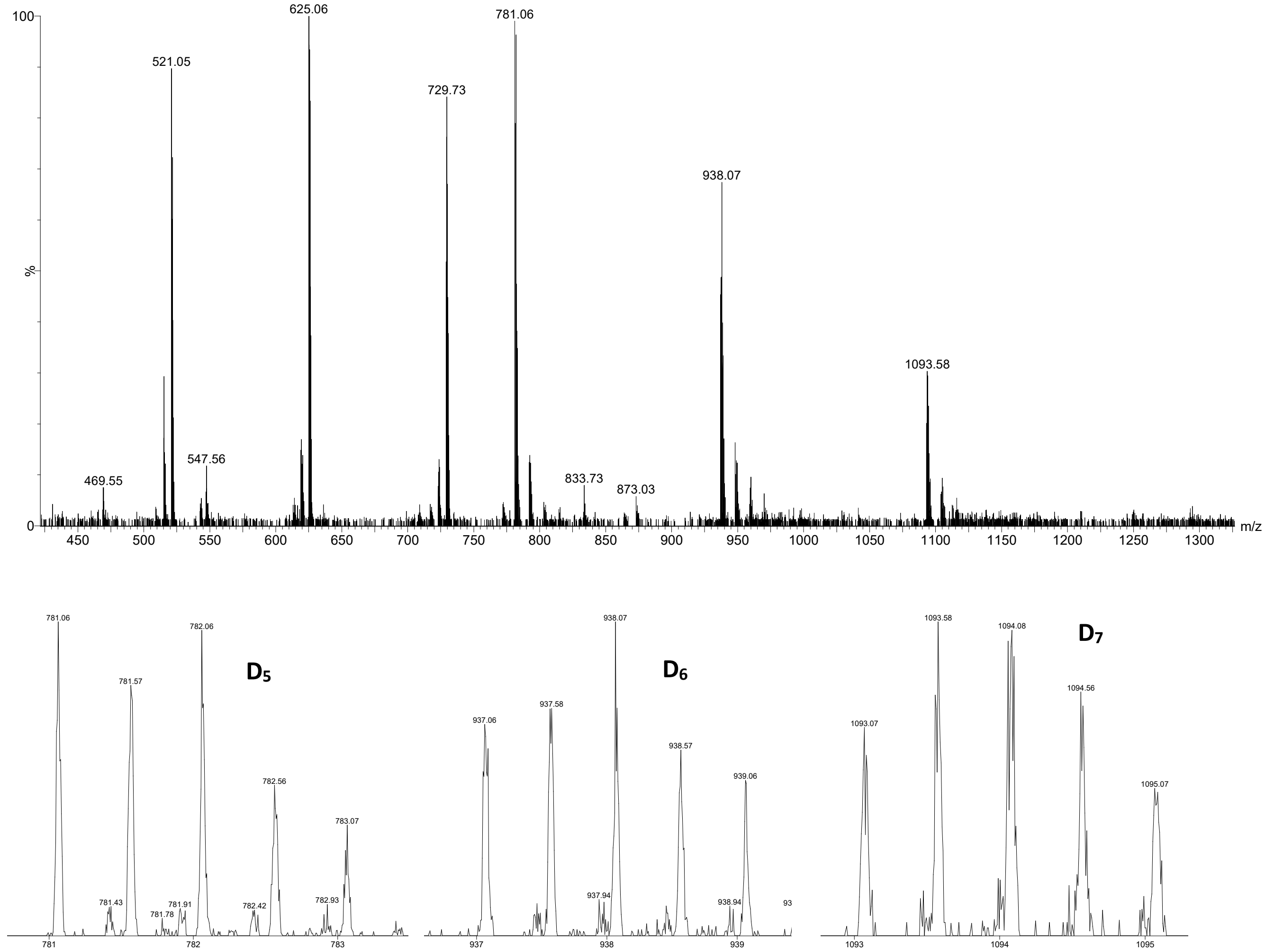

Supplementary Figure 58. *Mass spectrum of the cyclic* $\boldsymbol{D_5}$*,* $\boldsymbol{D_6}$ *and* $\boldsymbol{D_7}$ *from Supplementary Figure 54.* $\boldsymbol{D_5}$ *m/z calculated: 781.20* $[M+2H]^{2+}$*, 521.13* $[M+3H]^{3+}$*; m/z observed: 781.06* $[M+2H]^{2+}$*, 521.05* $[M+3H]^{3+}$*. Inset: isotopic profile at m/z 781.06.* $\boldsymbol{D_6}$ *m/z calculated: 937.24* $[M+2H]^{2+}$*, 625.14* $[M+3H]^{3+}$*; m/z observed: 937.06* $[M+2H]^{2+}$*, 625.06* $[M+3H]^{3+}$*. Inset: isotopic profile at m/z 937.06.* $\boldsymbol{D_7}$ *m/z calculated: 1093.28* $[M+2H]^{2+}$*, 729.18* $[M+3H]^{3+}$*; m/z observed: 1093.07* $[M+2H]^{2+}$*, 729.07* $[M+3H]^{3+}$*. Inset: isotopic profile at m/z 1093.07.*

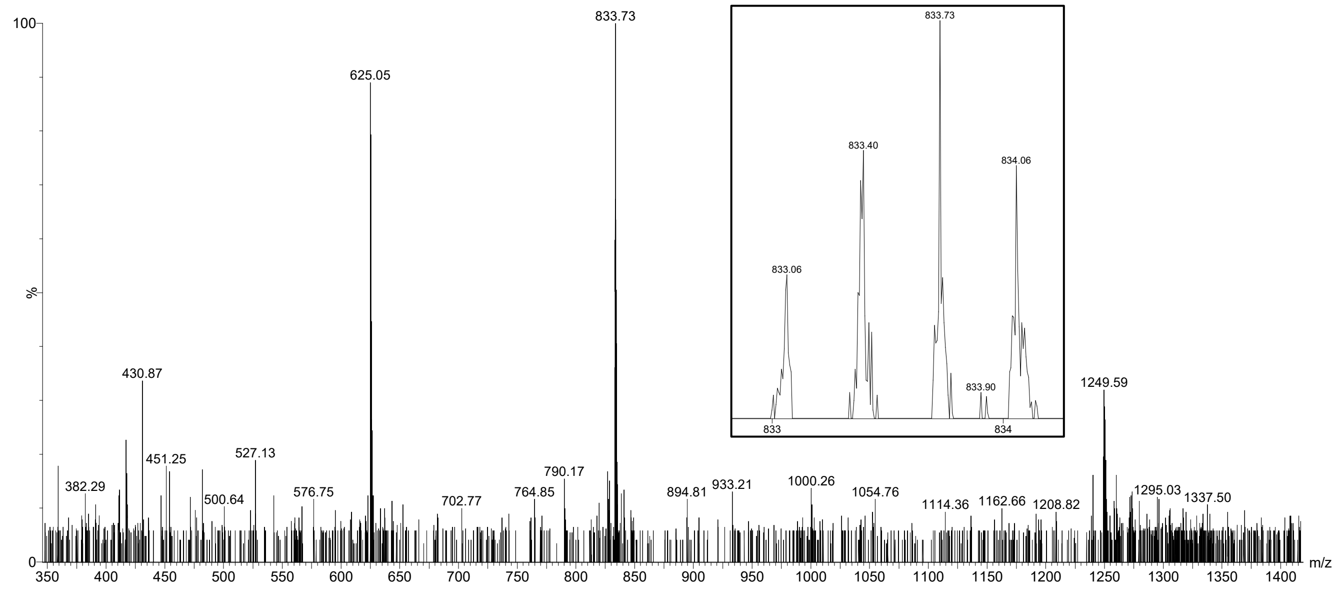

Supplementary Figure 59. *Mass spectrum of the cyclic* $\boldsymbol{D_8}$ *from Supplementary Figure 54. m/z calculated: 1249.32* $[M+2H]^{2+}$*, 833.21*

$[M+3H]^{3+}$, 625.16 $[M+4H]^{4+}$; m/z observed: 1249.04 $[M+2H]^{2+}$, 833.06 $[M+3H]^{3+}$, 625.05 $[M+4H]^{4+}$. Inset: isotopic profile at m/z 833.06.

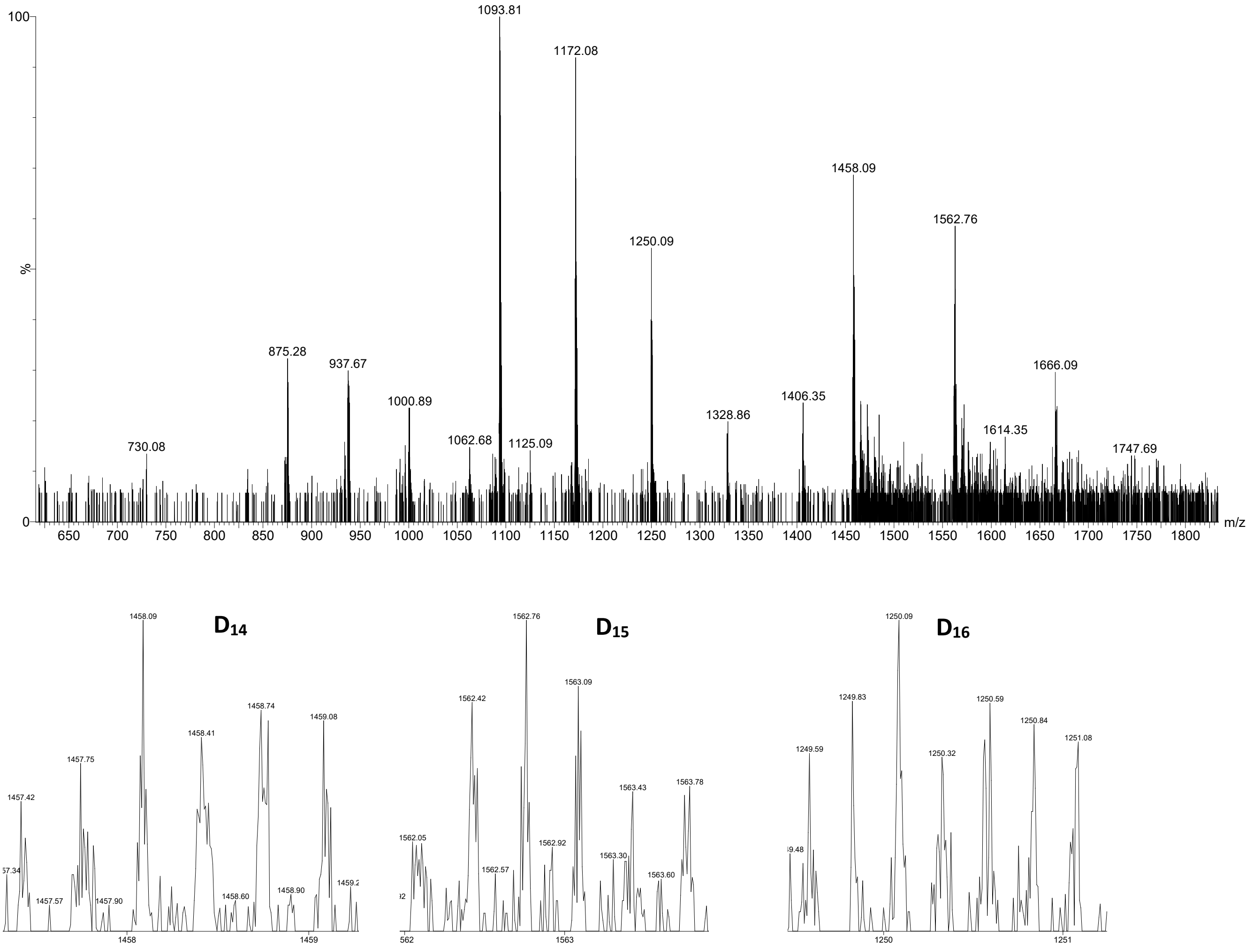

***Supplementary Figure 60**. Mass spectrum of the cyclic **D_{14}**, **D_{15}** and **D_{16}** from Supplementary Figure 54. **D_{14}** m/z calculated: 1457.37 $[M+3H]^{3+}$, 1093.28 $[M+4H]^{4+}$, 874.82 $[M+5H]^{5+}$; m/z observed: 1457.42 $[M+3H]^{3+}$, 1093.10 $[M+4H]^{4+}$, 874.66 $[M+5H]^{5+}$. Inset: isotopic profile at m/z 1457.42. **D_{15}** m/z calculated: 1561.40 $[M+3H]^{3+}$, 1173.30 $[M+4H]^{4+}$, 937.24 $[M+5H]^{5+}$; m/z observed: 1562.05 $[M+3H]^{3+}$, 1172.80 $[M+4H]^{4+}$, 937.08 $[M+5H]^{5+}$. Inset: isotopic profile at m/z 1562.05. **D_{16}** m/z calculated: 1665.43 $[M+3H]^{3+}$, 1249.32 $[M+4H]^{4+}$, 999.65 $[M+5H]^{5+}$; m/z observed: 1665.09 $[M+3H]^{3+}$, 1249.59 $[M+4H]^{4+}$, 999.47 $[M+5H]^{5+}$. Inset: isotopic profile at m/z 1249.59.*

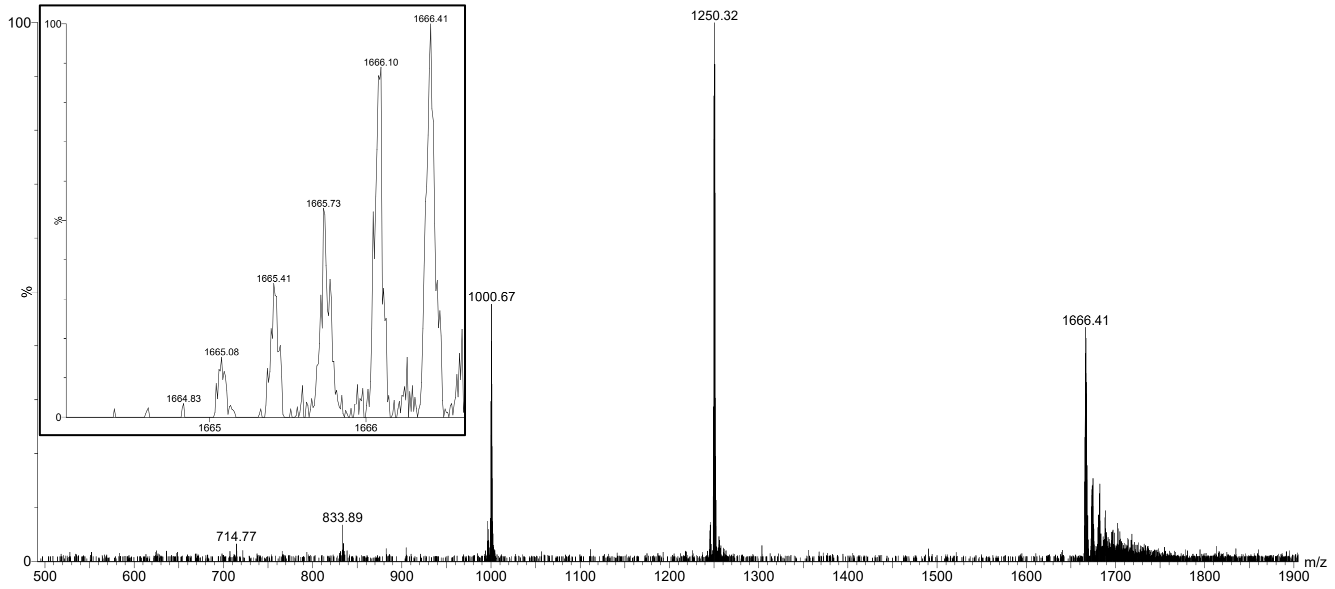

Supplementary Figure 61. *Mass spectrum of the cyclic* $\mathbf{D_{16}}$ *from Supplementary Figure 54. m/z calculated: 1665.43 [M+3H]$^{3+}$, 1249.32 [M+4H]$^{4+}$, 999.65 [M+5H]$^{5+}$; m/z observed: 1665.08 [M+3H]$^{3+}$, 1249.07 [M+4H]$^{4+}$, 999.45 [M+5H]$^{5+}$. Inset: isotopic profile at m/z 1665.08.*

3.4.5 Building block **E**

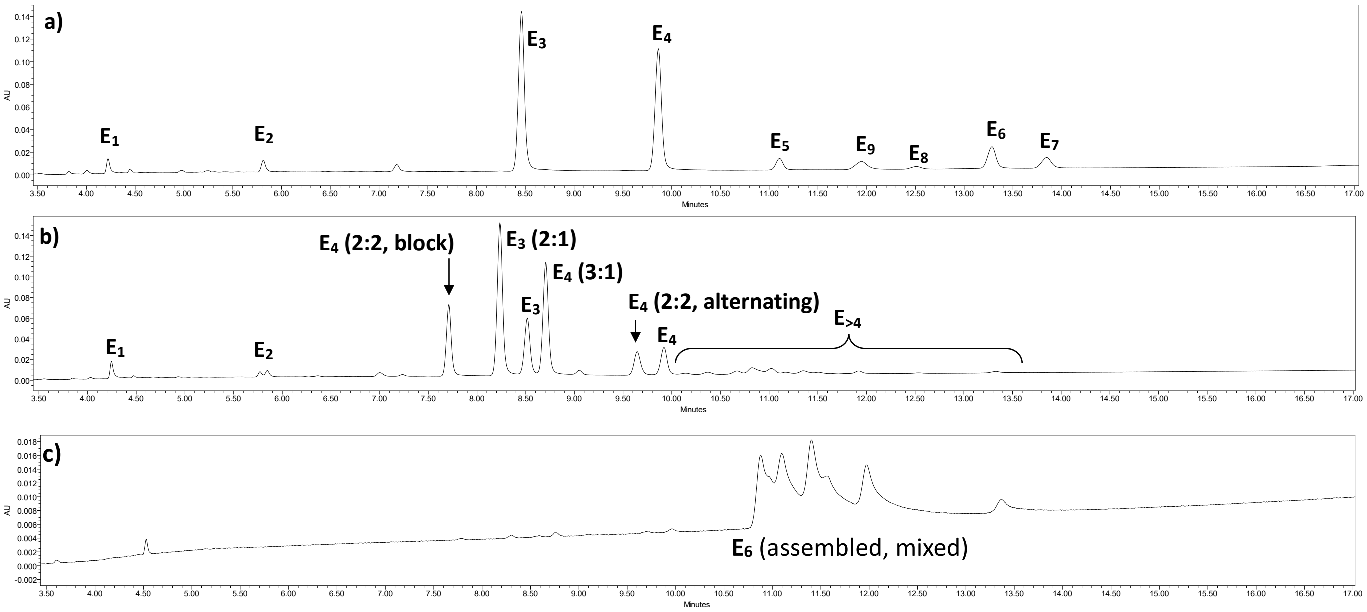

Supplementary Figure 62. *Typical UPLC traces of libraries made from a) enantiopure* **E** *and b) racemic* **E** *(3.8 mM in borate buffer with 4M GuHCl, pH 8.1) under constant mechanical agitation; diastereomers of (rac)-***E** *macrocycles separate on UPLC. Because the same species in these two traces show mostly identical elution time and have the same mass, only one mass spectrum for each species is provided below. Species with the same ring sizes but have different compositions are also provided with only one mass spectrum. Trace c) shows the final chromatogram of assembled (rac)-*$\mathbf{E_6}$ *(only* $\mathbf{E_6}$ *is found in the corresponding mass spectrum). At timepoints where the DCL is not yet fully converted to (rac)-*$\mathbf{E_6}$ *the peaks overlap with other macrocycles from* $\mathbf{E_4}$ *to* $\mathbf{E_9}$*, which is why these are taken together as* $\mathbf{E_{>4}}$ *in b) and in the kinetic traces in the main text (Figure 7f).*

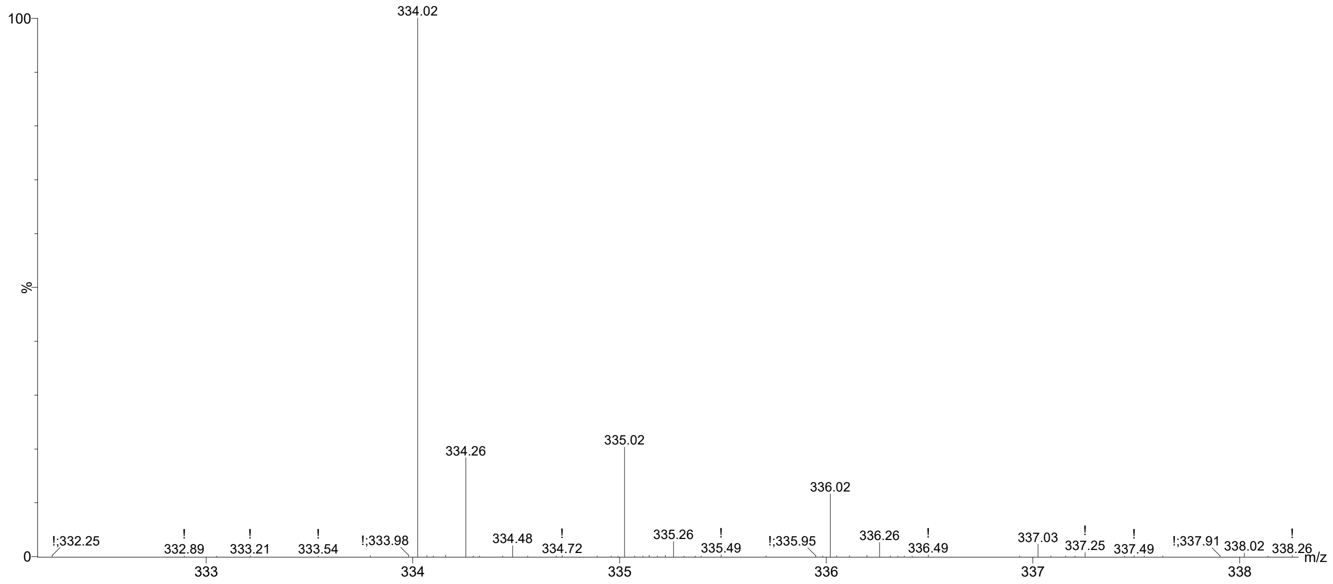

***Supplementary Figure 63**. Mass spectrum of the monomer **E_1** from Supplementary Figure 62. m/z calculated: 334.05 $[M+1H]^+$; m/z observed: 334.02 $[M+1H]^+$.*

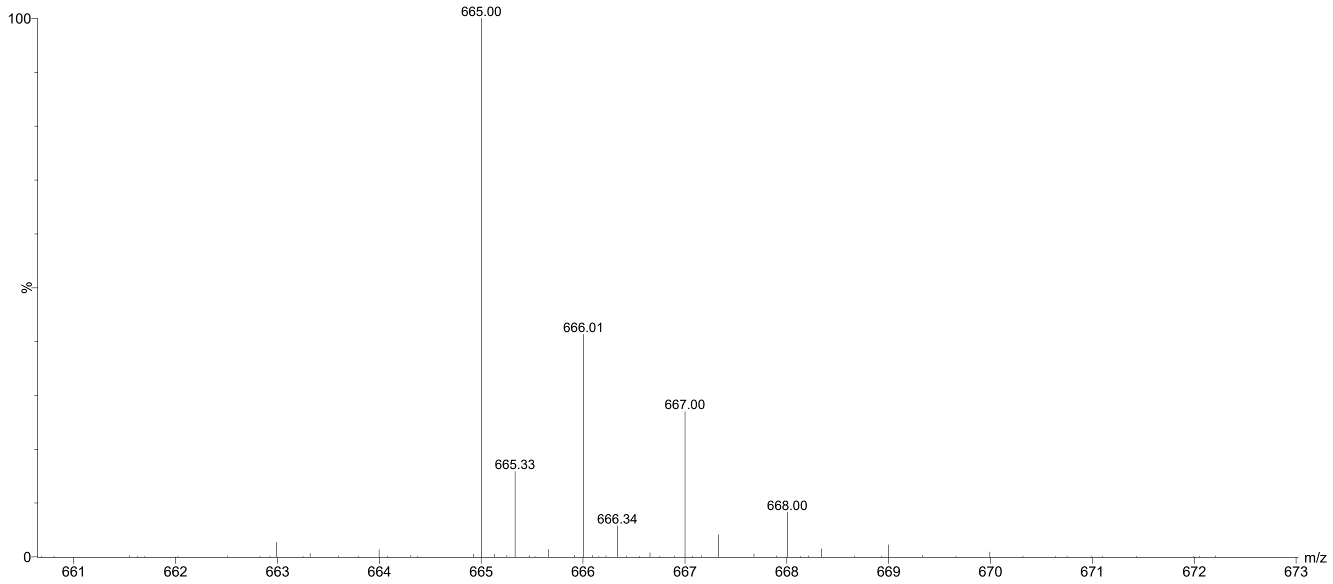

***Supplementary Figure 64**. Mass spectrum of the dimer **E_2** from Supplementary Figure 62. m/z calculated: 665.10 $[M+1H]^+$; m/z observed: 665.00 $[M+1H]^+$.*

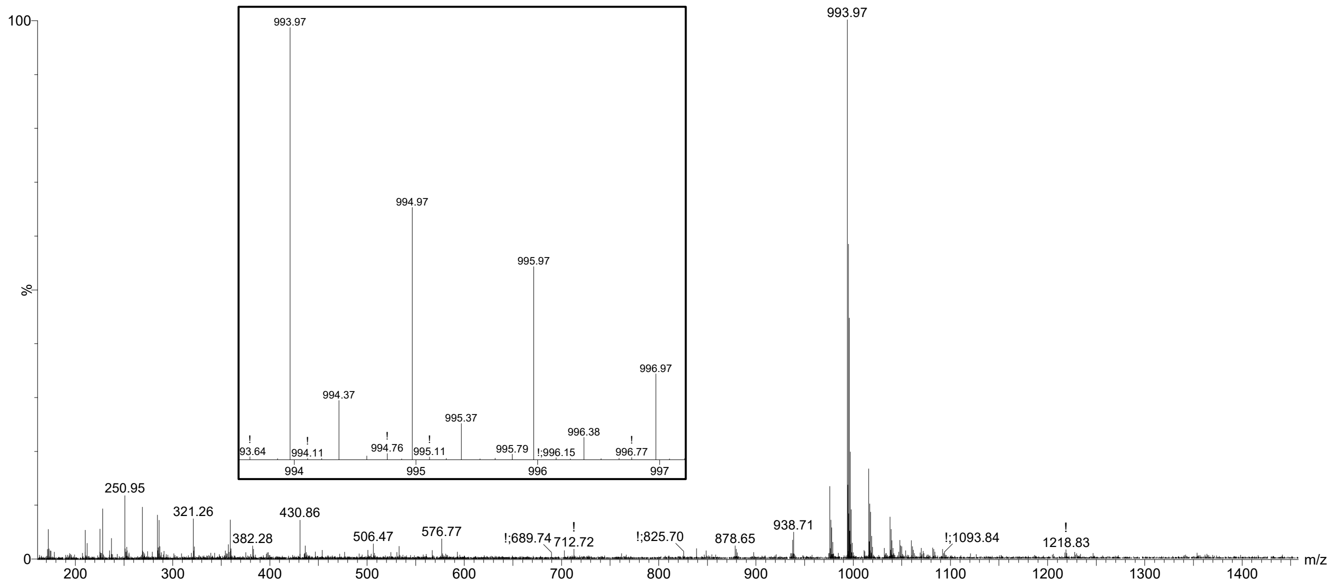

***Supplementary Figure 65**. Mass spectrum of the cyclic **E_3** from Supplementary Figure 62. m/z calculated: 994.15 $[M+1H]^+$; m/z observed: 993.97 $[M+1H]^+$. Inset: isotopic profile at m/z 993.97.*

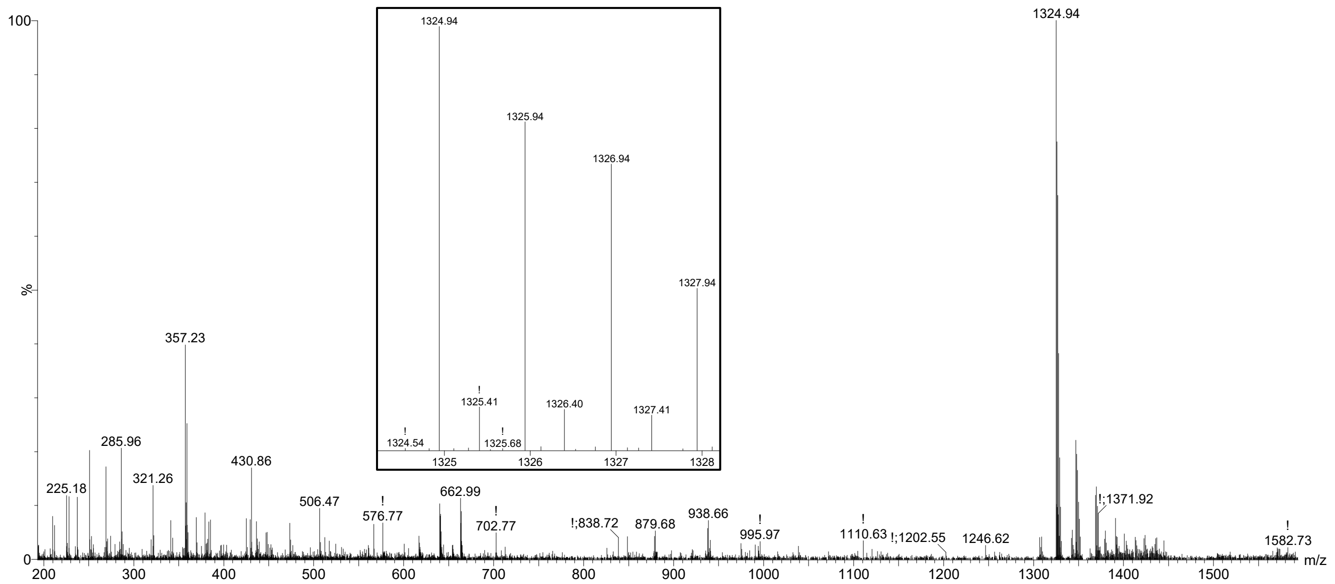

***Supplementary Figure 66**. Mass spectrum of the cyclic **E_4** from Supplementary Figure 62. m/z calculated: 1324.94 $[M+1H]^+$; m/z observed: 1324.94 $[M+1H]^+$. Inset: isotopic profile at m/z 1324.94.*

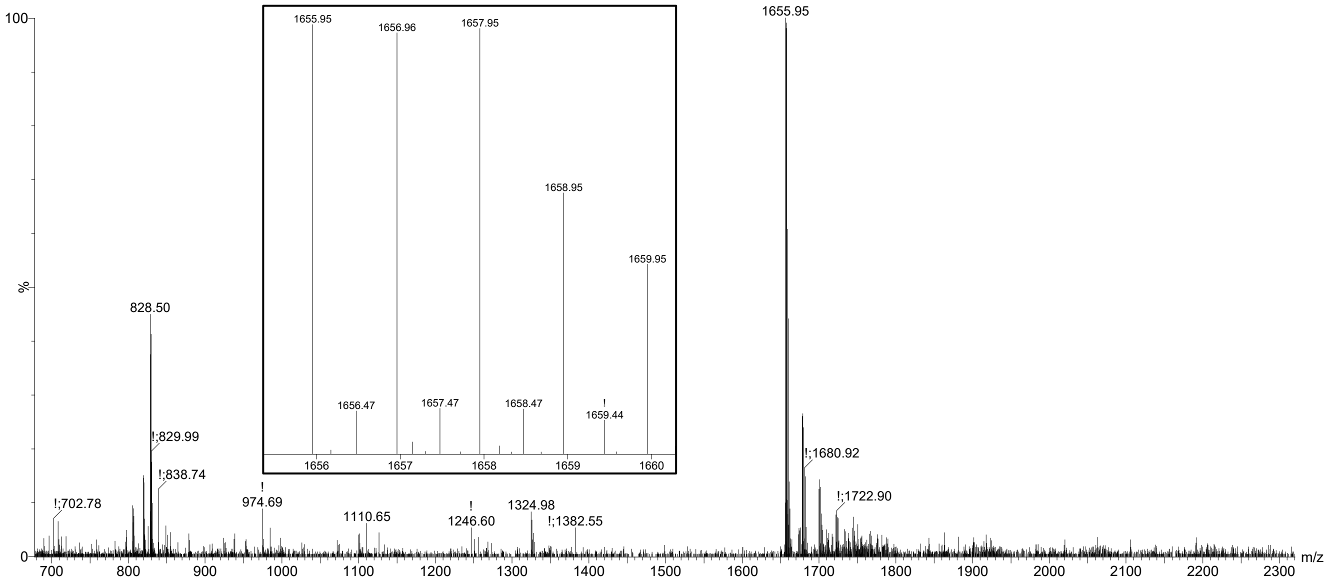

Supplementary Figure 67. *Mass spectrum of the cyclic* $\boldsymbol{E_5}$ *from Supplementary Figure 62. m/z calculated: 1656.25 [M+1H]*$^+$*, 828.62 [M+2H]*$^{2+}$*; m/z observed: 1655.95 [M+1H]*$^+$*, 828.50 [M+2H]*$^{2+}$*. Inset: isotopic profile at m/z 1655.95.*

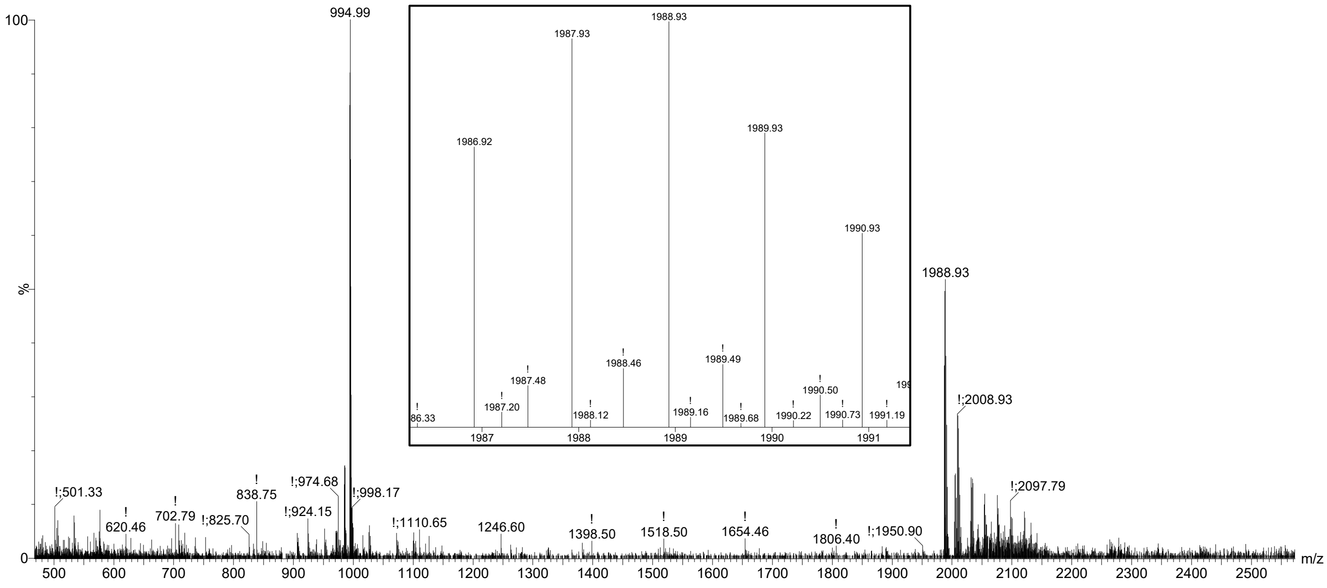

Supplementary Figure 68. *Mass spectrum of the cyclic* $\boldsymbol{E_6}$ *from Supplementary Figure 62. m/z calculated: 1987.3 [M+1H]*$^+$*, 994.15 [M+2H]*$^{2+}$*; m/z observed: 1986.92 [M+1H]*$^+$*, 993.98 [M+2H]*$^{2+}$*. Inset: isotopic profile at m/z 1986.92.*

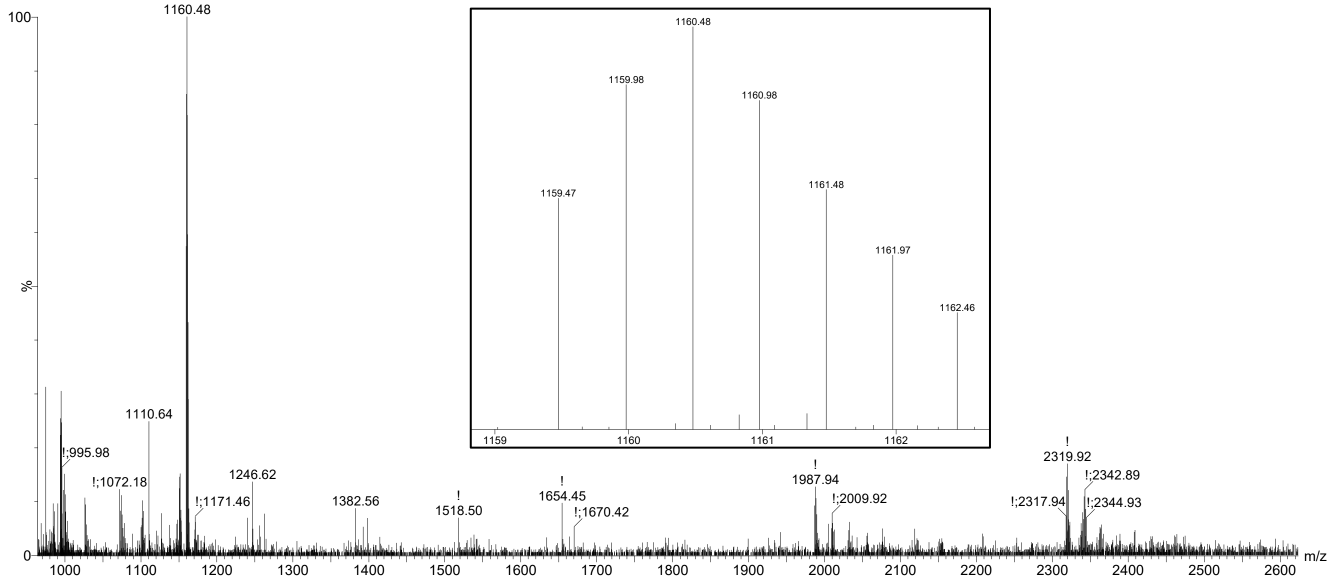

***Supplementary Figure 69**. Mass spectrum of the cyclic **E_7** from Supplementary Figure 62. m/z calculated: 2318.35 [M+1H]$^{1+}$, 1159.68 [M+2H]$^{2+}$; m/z observed: 2317.95 [M+1H]$^{1+}$, 1159.47 [M+2H]$^{2+}$. Inset: isotopic profile at m/z 1159.47.*

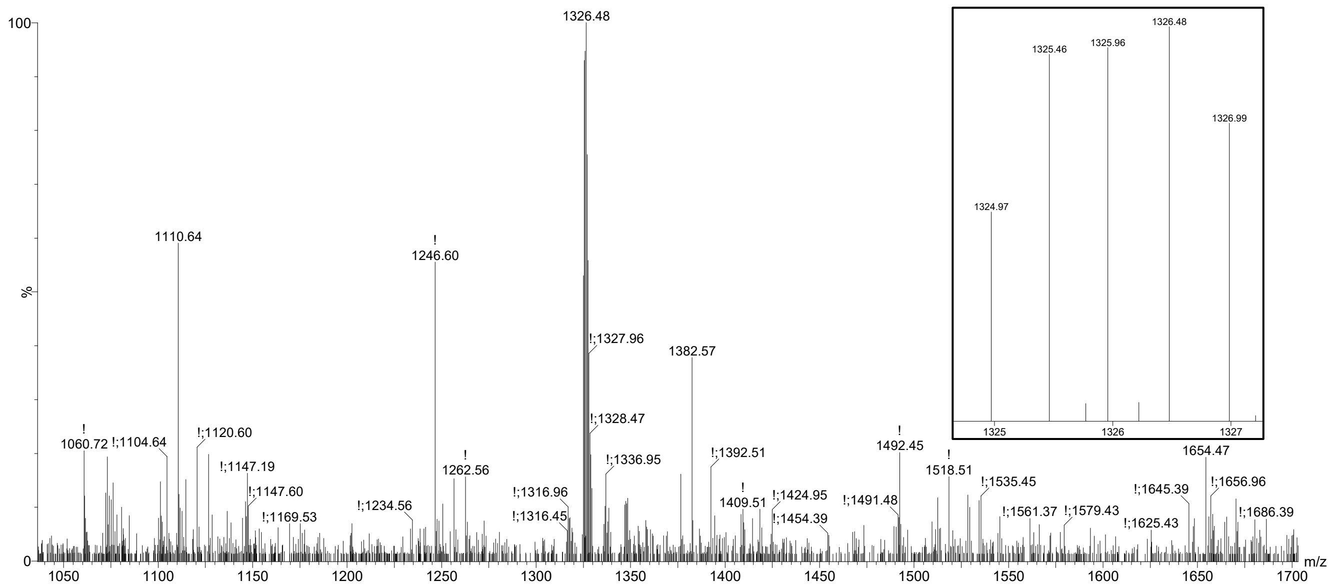

***Supplementary Figure 70**. Mass spectrum of the cyclic **E_8** from Supplementary Figure 62. m/z calculated: 1325.2 [M+2H]$^{2+}$; m/z observed: 1324.97 [M+2H]$^{2+}$. Inset: isotopic profile at m/z 1324.97.*

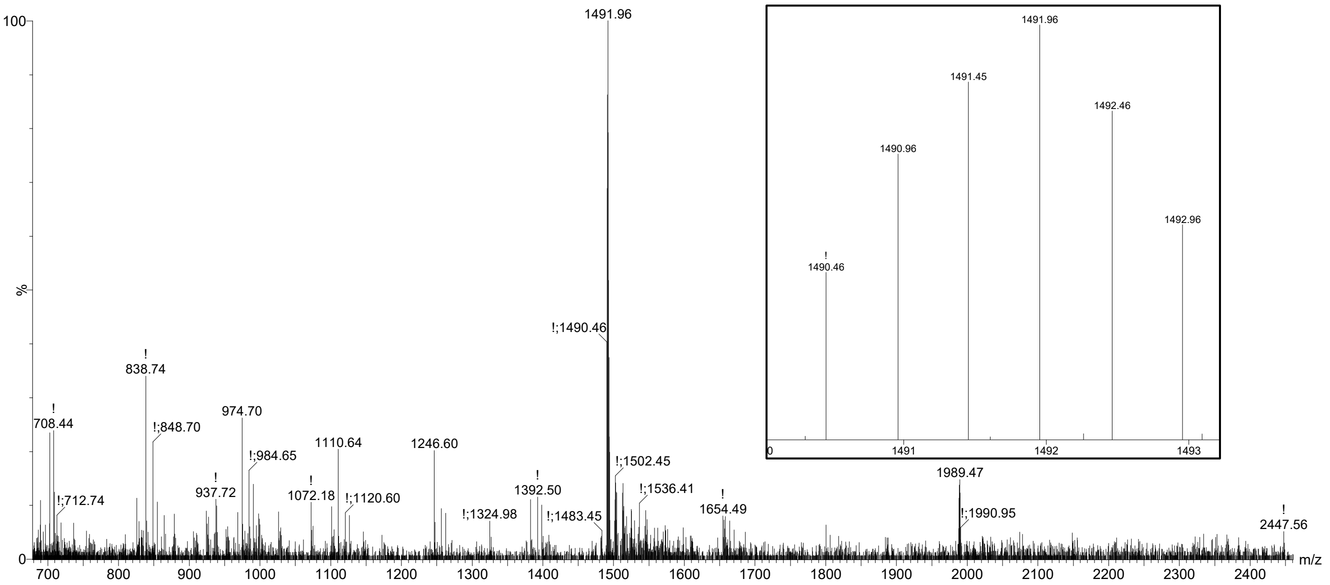

Supplementary Figure 71. *Mass spectrum of the cyclic* ***E₉*** *from Supplementary Figure 62. m/z calculated: 1490.73 [M+2H]*$^{2+}$*; m/z observed: 1490.46 [M+2H]*$^{2+}$*. Inset: isotopic profile at m/z 1490.46.*

3.5 MALDI-TOF

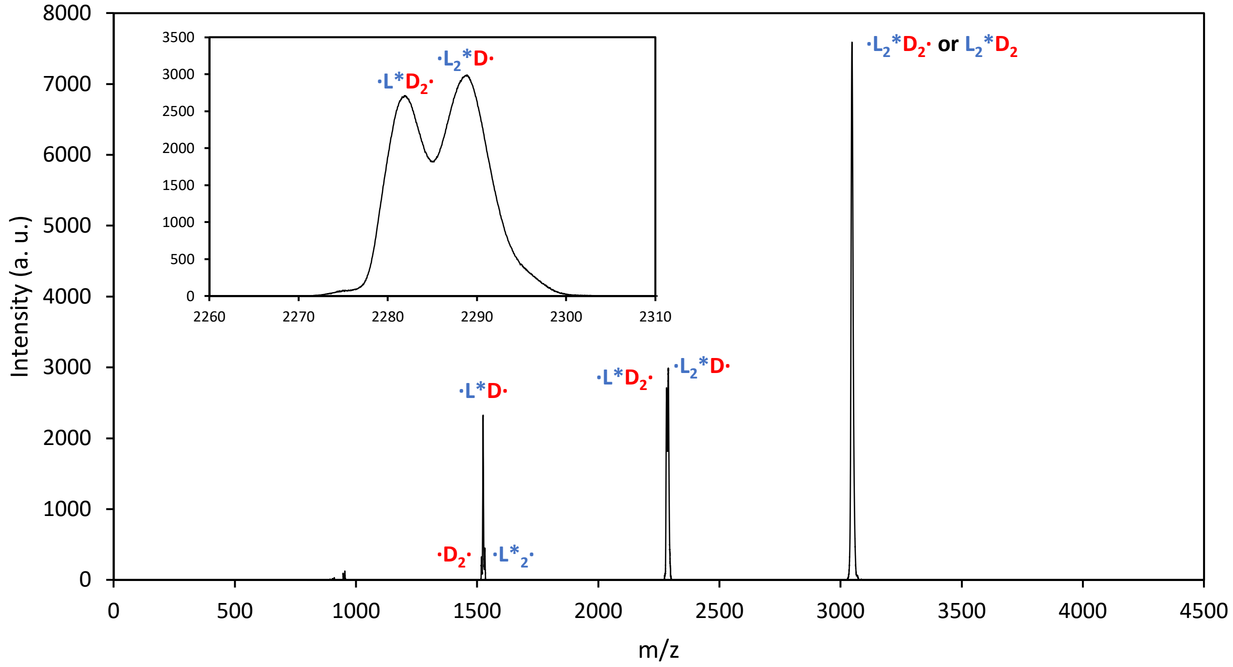

Supplementary Figure 72. *Full MALDI-TOF spectrum of the isolated $(L^*{}_2D_2)$-**A_4**. It shows the full tetramer (calc.: 3044.36, found: 3047.07 m/z), the peak for the two trimer fragments ·L^*D_2· (calc.: 2280.02, found: 2281.98 m/z) and ·$L^*{}_2D$· (calc.: 2287.02, found: 2288.89 m/z) and the peak for the three dimer fragments ·D_2· (calc.: 1515.68, found: 1517.79), ·L*D· (calc.: 1522.68, found: 1524.76), and ·$L^*{}_2$ (calc.: 1529.68, found: 1531.89) (cf. Supplementary Figure 7d for a zoom on the dimers and further discussion). All m/z values are $[M+H]^+$; the "calc." values are monoisotopic masses while the "found" values are peak maxima, as the instrument's resolution is too low to distinguish isotope profiles. Both are not directly comparable but serve as an indication that the observed peaks correspond to the respective **A/A*** species. Inset: zoom on the trimer fragment peaks.*

4 References

[1] B. Bartolec, M. Altay, S. Otto, *Chem. Commun.* **2018**, *54*, 13096–13098.

[2] O. Markovitch, J. Ottelé, O. Veldman, S. Otto, *Commun. Chem.* **2020**, *3*, 1–4.

[3] B. Liu, J. Wu, M. Geerts, O. Markovitch, C. G. Pappas, K. Liu, S. Otto, *Angew. Chem. Int. Ed.* **2022**, *61*, e202117605.

[4] G. Schaeffer, M. J. Eleveld, J. Ottelé, P. C. Kroon, P. W. J. M. Frederix, S. Yang, S. Otto, *J. Am. Chem. Soc.* **2022**, *144*, 6291–6297.
